# Regio- and Diastereoselective Vicinal Aminobromination of Electron Deficient Olefins via Phosphorus-Based GAP Protocol

**DOI:** 10.3389/fchem.2021.742399

**Published:** 2021-09-08

**Authors:** Anis Ur Rahman, Nighat Zarshad, Iltaf Khan, Faisal Faiz, Guigen Li, Asad Ali

**Affiliations:** ^1^ Institute of Chemistry and BioMedical Sciences, School of Chemistry and Chemical Engineering, Nanjing University, Nanjing, China; ^2^ School of Chemistry and Chemical Engineering, Southeast University, Nanjing, China; ^3^ Key Laboratory of Functional Inorganic Material Chemistry, Ministry of Education, Heilongjiang University, China; ^4^ State Key Laboratory of Analytical Chemistry for Life Science, School of Chemistry & Chemical Engineering and Center of Materials Analysis, Nanjing University, Nanjing, China; ^5^ Department of Chemistry and Biochemistry, Texas Tech University, Lubbock, TX, United States; ^6^ Department of Chemistry, Faculty of Chemical and Life Sciences, Abdul Wali Khan University, Mardan, Pakistan

**Keywords:** aziridinium, diastereoselectivity, iodobenzene diacetate, nitrogen/halogen source, protecting groups

## Abstract

Chemical synthesis based on Group-Assisted Purification chemistry (GAP) has been prolifically used as a powerful, greener and ecofriendly tool so far. Herein, we report hypervalent iodine (III) mediated regio- and diastereoselective aminobromination of electron-deficient olefins using group-assisted purification (GAP) method. By simply mixing the GAP auxiliary-anchored substrates with TsNH_2_–NBS as nitrogen/bromine sources and PhI(OAc)_2_ as a catalyst, a series of vicinal bromoamines with multifunctionalities were obtained in moderate to excellent yields (53–94%). The vicinal bromoamines were obtained without column chromatography and/or recrystallization simply by washing the crude mixtures with cosolvents and thus avoiding wastage of silica, solvents, time, and labor. The GAP auxiliary is recyclable and reusable.

## Introduction

Aminohalogenation of olefins, an important difunctionalization reaction, allows the direct construction to C−N and C−halogen double bonds which are versatile synthetic intermediates for pharmaceutically and biologically important molecules (Gao et al., 2005; Yeung et al., 2006b). The intramolecular and/or intermolecular replacement of labile halogen moieties with multifarious nucleophiles leads to precursors like vicinal diamines, lactams, amino alcohols α,β-dehydroamino acids, amino aldehydes and aziridines (Ling et al., 1996; Van and De Kimpe, 2000; Klepacz and Zwierzak, 2001; Chen et al., 2005; Li et al., 2007; Ghorai et al., 2011; Schröder et al., 2017; Thakur et al., 2017).

Since the aminohalogenation reaction was discovered several decades ago, a variety of synthetic techniques have been created to provide this capability. Various reagent systems (halogen/nitrogen sources) such as TsNH_2_–NBS (Thakur et al., 2003; Chen et al., 2009a; Chen et al., 2009b; Shaikh et al., 2009; Wei et al., 2009a; Wei et al., 2009b; Cai et al., 2011; Yu et al., 2017), cyanamide–NBS (Ponsold and Ihn, 1970), N-bromoacetamide (Yeung et al., 2006a; Yeung et al., 2006b), N,N-dihalosulfonamides (Kharasch and Priestley, 1939), S,S-dimethyl-N-(p-toluenesulfonyl)sulfilimine–NBS (Raghavan et al., 2001), BocNH_2_/BocNBr_2_ (Chen et al., 2013), N,N-dihalocarbamates (Śliwińska and Zwierzak, 2003) and N-halocarbamates (Driguez et al., 1978) have been designed to carry out this transformation. To achieve high yields, excellent regioselectivities and diastereoselectivities, our group as well as others have developed efficient catalytic systems which comprise: metal and nonmetal powders (Chen et al., 2009b; Wei et al., 2009a), metal oxides (Thakur et al., 2003; Shaikh et al., 2009) and metal salts (Albone et al., 1998; Ando et al., 1998; Li et al., 1999; Li et al., 2000; Wei et al., 2001; Yeung et al., 2006a; Wang et al., 2008; Chen et al., 2009a; Wei et al., 2009b; Yadav et al., 2009), organic catalysts like hypervalent iodines (Fan et al., 2007; Wang and Wu, 2007; Wu et al., 2008), phosphoric acid or phosphate (Chen et al., 2010; Huang et al., 2011; Alix et al., 2012; Xie et al., 2013), noncatalytic routes which utilize Bronsted acids (Wu and Wang, 2007) such as H_2_SO_4_ or ionic liquid media [Bmim][BF_4_](Xu et al., 2004). Though considerable progress has been made in this area, drastic reaction conditions, procedural complexities, the use of metal catalysts and contamination of materials by metal traces (Chen et al., 2003a; Garrett and Prasad, 2004; Huang and Shaughnessy, 2006) limit their application. Besides, the study of efficient highly regio- and stereoselective methods which could reduce the formation of side products remains challenging. Purification techniques such as column chromatography and recrystallization are commonly used in the above mentioned syntheses.

The development of environmentally benign and eco-friendly greener reaction protocol is ubiquitous both in academia and the pharmaceutical industry (Shi et al., 2008). GAP chemistry, recently introduced by our group, fulfills the afford-mentioned criteria of greener chemistry by avoidance of separation, workup, recrystallization, and column chromatography. The product is obtained by merely washing the reaction mixture with a combination of more polar and less polar solvents (Wang et al., 2013; Chennapuram et al., 2014; Dommaraju and Prajapati, 2015; Seifert et al., 2016; Patel et al., 2019; Li et al., 2020a; Li et al., 2020b; Li et al., 2020c). Polarity difference between the solvents plays a key role in the isolation of products, i.e., the impurities get dissolved in washing solvents and the GAP-coupled product remains insoluble clustered together. Keeping in view the greener aspect of GAP chemistry, here we report for the first time hypervalent iodine (III) mediated regio- and diastereoselective vicinal aminobromination of GAP-tailored electron-deficient olefins via GAP protocol.

## Results and Discussion

Based on our prior research, we were interested in aminobromination of α,β-unsaturated cinnamic acids, which are challenging due to the formation of regio- and diastereomeric products. To develop conditions for regio- and diastereoselective transformation, we began to prepare the GAP coupled intermediate 1a-k and 2a-k in our laboratory according to the literature procedure (Rahman et al., 2020) given in supporting information. To optimize the reaction conditions, we initiated the study with the GAP anchored intermediate 1a as the test substrate, p-toluenesulfonamide (4-TsNH_2_) and N-bromosuccinimide (NBS) as the nitrogen and bromine source respectively. To our delight, product 3a was isolated in 60% yield after 24 h with a dr value 7:1 when 1a was treated with NBS (1.5 eq) and 4-TsNH_2_ (1.5 eq) in dichloromethane at room temperature without any catalyst. Lower yields were obtained with other bromine sources (Table 1, entries 2–4). With NBS as the bromine source, a series of hypervalent iodine and transition metal catalysts were subsequently employed. The yield was significantly improved with iodobenzene diacetate (PhI(OAc)_2_), and aminobromine product was isolated in a chemical yield of 78% with diastereoselective ratio of 7:1 (Table 1, entry 8). Refluxing this reaction mixture further enhanced the yield up to 82% (Table 1, entry 13). An even more increase in yield was observed when 2 eq of each NBS and 4-TsNH_2_ was added to the reaction medium (Table 1, entry 14). The yield was further improved to 90% with a longer reaction time (48 h) (Table 1, entry 15). We then utilized the catalytic activity of other iodine catalysts like PIFA (PhI(OCOCF_3_)_2_) and Koser’s reagent (PhI(OH) (4-TsOH)) in this transformation; only PhI(OAc)_2_ could give the terminal product in higher yield (Table 1, entry 15). Except for CHCl_3_ and CH_3_CN, poorer results were obtained at reflux temperature with other solvents examined when the reaction was performed with 20 mol% of PhI(OAc)_2_ as the catalyst and 2 equiv. of NBS and 4-TsNH_2_ (Table 1, entries 18–23). A yield of 67% was obtained when the catalyst loading was decreased to 10 mol%. Control experiments showed that both NBS and the 4-TsNH_2_ were important for the reaction and that using activated molecular sieves 4Å generally increased the yield and selectivity.

**TABLE 1:** Optimization of the reaction conditions.^a^ 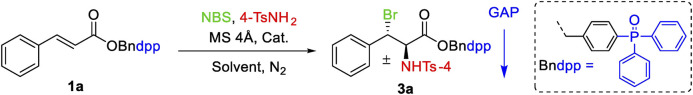

Entry	Catalyst	Br source (equiv.)	Time (h)	Solvent	Yield^b^ (%)	dr^c^
1		NBS (1.5)	24	CH_2_Cl_2_	60	7:1
2		TBCO (1.5)	24	CH_2_Cl_2_	31	5:1
3		PhCONHBr (1.5)	24	CH_2_Cl_2_	47	4:1
4		DBDMH (1.5)	24	CH_2_Cl_2_	53	4:1
5	Pd(OAc)_2_	NBS (1.5)	24	CH_2_Cl_2_	69	10:1
6	Mn(OAc)_2_	NBS (1.5)	24	CH_2_Cl_2_	60	4:1
7	FeCl_3_	NBS (1.5)	24	CH_2_Cl_2_	54	4:1
8	PhI(OAc)_2_	NBS (1.5)	24	CH_2_Cl_2_	78	7:1
9	ZnCl_2_	NBS (1.5)	24	CH_2_Cl_2_	35	6:1
10	CuI	NBS (1.5)	24	CH_2_Cl_2_	52	10:1
11	Cu(Otf)_2_	NBS (1.5)	24	CH_2_Cl_2_	63	7:1
12	Sc(Otf)_3_	NBS (1.5)	24	CH_2_Cl_2_	51	4:1
13^e^	PhI(OAc)_2_	NBS (1.5)	24	CH_2_Cl_2_	82	7:1
14^e^	PhI(OAc)_2_	NBS (2.0)	24	CH_2_Cl_2_	85	7:1
15^e^	PhI(OAc)_2_	NBS (2.0)	48	CH_2_Cl_2_	90	7:1
16^e^	PhI(OH) (4-TsOH)	NBS (2.0)	48	CH_2_Cl_2_	70	8:1
17^e^	PhI(OCOCF_3_)_2_	NBS (2.0)	48	CH_2_Cl_2_	74	8:1
18^e^	PhI(OAc)_2_	NBS (2.0)	48	CH_3_CN	77	7:1
19^e^	PhI(OAc)_2_	NBS (2.0)	48	CHCl_3_	94	7:1
20^e^	PhI(OAc)_2_	NBS (2.0)	48	PhMe	28	
21^e^	PhI(OAc)_2_	NBS (2.0)	48	THF	--	--
22^e^	PhI(OAc)_2_	NBS (2.0)	48	Et_2_O	--	--
23^e^	PhI(OAc)_2_	NBS (2.0)	48	EtOAc	--	--
24^e^ ^,^ ^f^	PhI(OAc)_2_	NBS (2.0)	48	CHCl_3_	61	7:1

a Unless otherwise specified, all reactions were performed with 0.15 mmol of 11a, 20 mol% of the catalyst, 4-TsNH_2_ and Br source (1:1), 75 mg of MS 4 Å in 1.5 ml of solvent at room temperature under N_2_.

b Isolated yields with GAP washing (for entries 2, 3, 9 and 20 GAP washing was not conducted).

c The dr values were determined by the analysis of.

d H NMR spectra.

e For entries 13–23, the reactions were performed at reflux.

f The reaction was carried out at 10 mol% of the catalyst.

After optimizing the conditions for aminobromination reaction, the substrate scope was subsequently explored. The results are shown in Table 2. A wide range of N-(4-(diphenylphosphoryl)benzyl) cinnamates 1a-k bearing different aryl groups with a variety of electron-donating (EDG) (such as methyl and methoxy) and electron-withdrawing groups (EWG) (floro, chloro bromo, nitro) were investigated which provided moderate to high yields (53–94%). As shown in Table 2, with regards to the EDG on the aromatic ring of cinnamic substrates 1b-1f, the addition reactions were well tolerated to produce the relevant adducts in good yields (Table 2, 2b-2f). Both the substrates 1b and 1c with an ortho-MeC_6_H_4_ and a para-MeC_6_H_4_ group delivered the corresponding products 3b and 3c smoothly in 85 and 89% yields respectively. Similarly, the product 3 days with ortho-OMeC_6_H_4_ was isolated in a high yield of 80%. The di-OMe and tri-OMe substituted substrates were even more effective for the reaction (Table 2, 3e, 3f). On the other hand, substrates bearing EWG on the aromatic rings generally decreased the yield under the same conditions (Table 2, 3g-3j). Importantly, halogen (Br or F) groups were almost consistent with the conditions, offering 3g, 3h and 3i in moderate yields. The lowest yield of 53% was obtained for 3j, which had a Cl group at the ortho-position and an NO_2_ group at para-position. The substrate with a naphthyl group reduced the yield to 81% under the same conditions but enhanced the diastereoselectivity (Table 2, 3k).

**TABLE 2:** Substrate scope of aminobromination of N-(4-(diphenylphosphoryl)benzyl) cinnamates 1a-k.

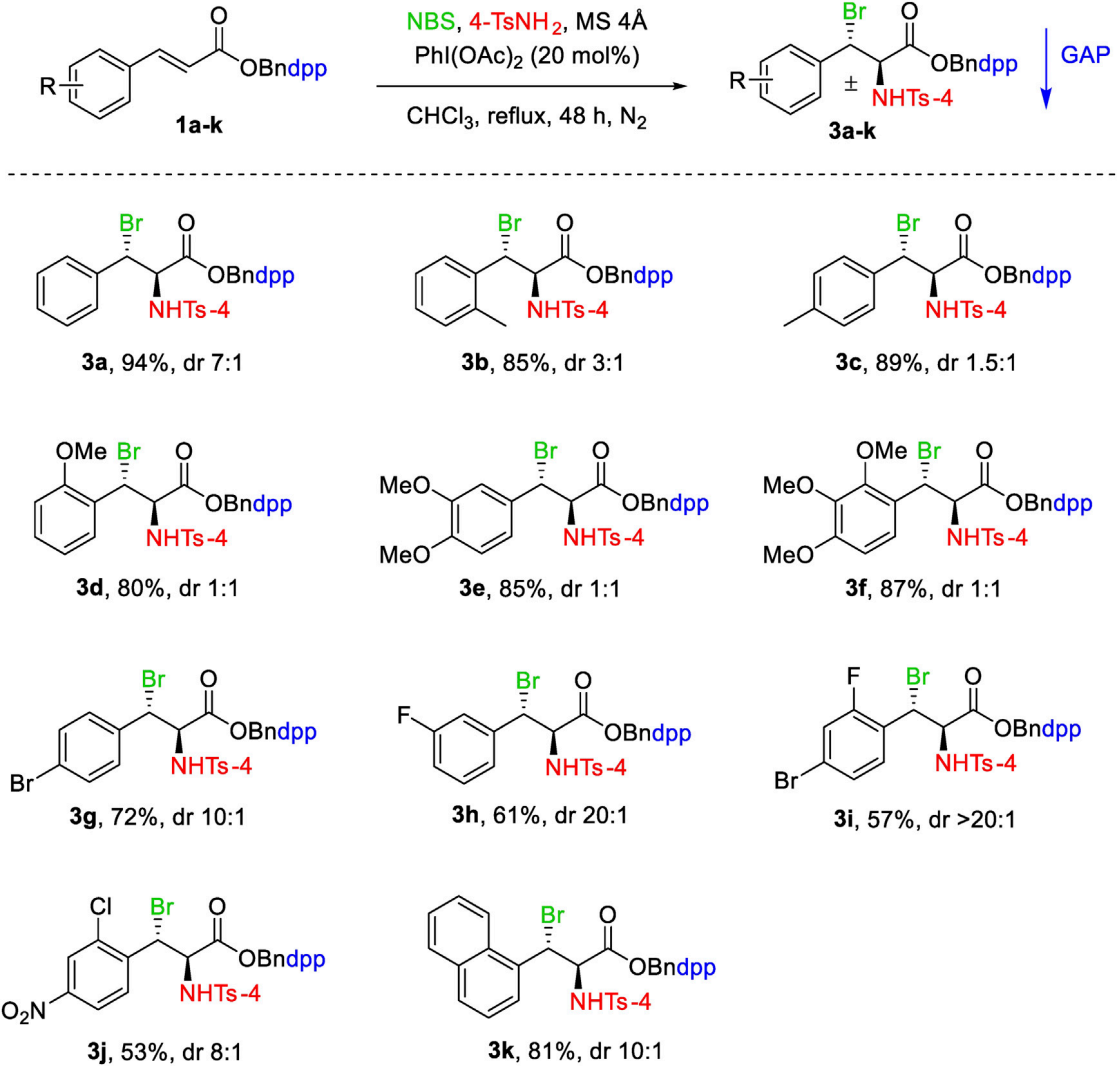

Unless otherwise specified, all reactions were performed with 0.3 mmol of 1a-k, 0.6 mmol of 4-TsNH_2_, 0.6 mmol of NBS, 150 mg of MS 4Å in 3 ml of chloroform at reflux under N_2_. The dr values were determined by the analysis of ^1^H NMR spectra. Isolated yields with GAP washing.

In addition to N-(4-(diphenylphosphoryl) benzyl) cinnamates, N-(4-(diphenylphosphoryl) benzyl) cinnamamides 2a-k were then exposed to aminobromination under the optimized reaction conditions for 1a-k. The reaction was applicable in the presence of 20 mol% of PhI(OAc)_2_ in chloroform, substrate 2a was successfully converted in 48 h at reflux temperature to haloamine product 4a in 78% yield with a diastereoselective ratio of 18:1.

As shown in Table 3, this transformation can be extended to a variety of N-(4-(diphenylphosphoryl)benzyl) cinnamamides 2a-k to provide moderate to high yields (56–81%). The substrates with EWG and EDG display substantial variations in reaction reactivity and regioselectivity. Aminobromination was greatly facilitated by the presence of a strong EDG on the benzene ring, affording products in high yields and good to excellent diastereoselectivity (Table 3, 4b–4e). The substrate with EWG on the aromatic ring, as expected, resulted in a lower yield (Table 3, 4f–4j). The substrate with a naphthyl group, however, had no significant effect on the yield under the same conditions and lowered the diastereoselectivity (Table 3, 4k).

**TABLE 3:** Substrate scope of aminobromination of N-(4-(diphenylphosphoryl)benzyl) cinnamamides 2a-k.

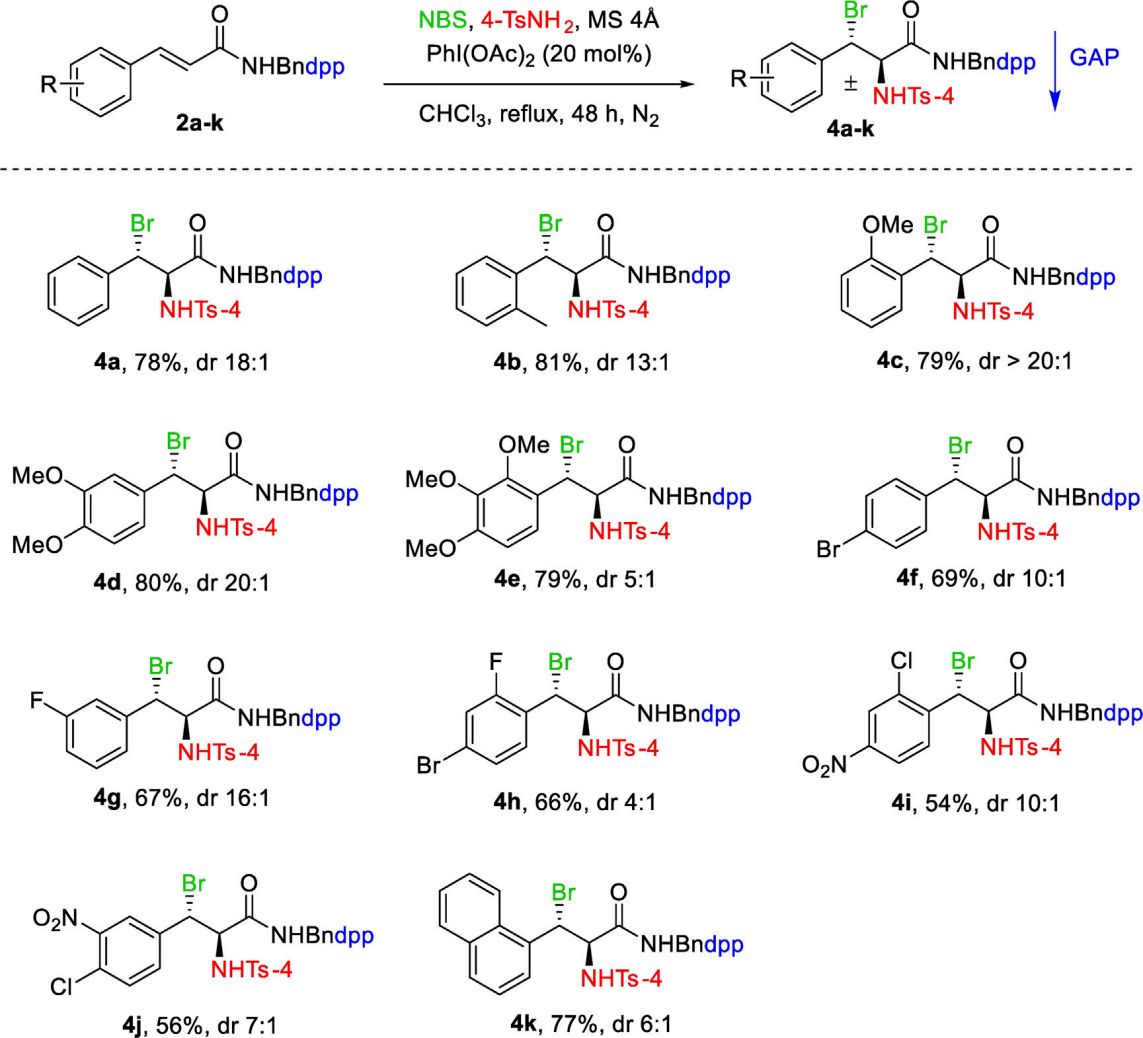

Unless otherwise specified, all reactions were performed with 0.3 mmol of 2a-k, 0.6 mmol of 4-TsNH_2_, 0.6 mmol of NBS, 150 mg of MS 4Å in 3 ml of chloroform at reflux under N_2_. The dr values were determined by the analysis of ^1^H NMR spectra. Isolated yields with GAP washing.

From Table 2, 3, we further observed that EWG and EDG on the benzene ring had a significant impact on the diastereoselectivity of cinnamates and cinnamamides which is generally governed by the GAP auxiliaries. In the case of cinnamates, EDG resulted in low diastereoselectivity than EWG. For cinnamamides, however, EDG had higher diastereoselectivity than EWG. This variation in diastereoselectivity of both derivatives could be attributed to stereoelectronic factors.

The feasibility of this procedure was studied by conducting the reaction on a gram scale for the starting materials 1a and 2a, which resulted in 85 and 73% yields for the products 3a and 4a, respectively.

**SCHEME 1:**
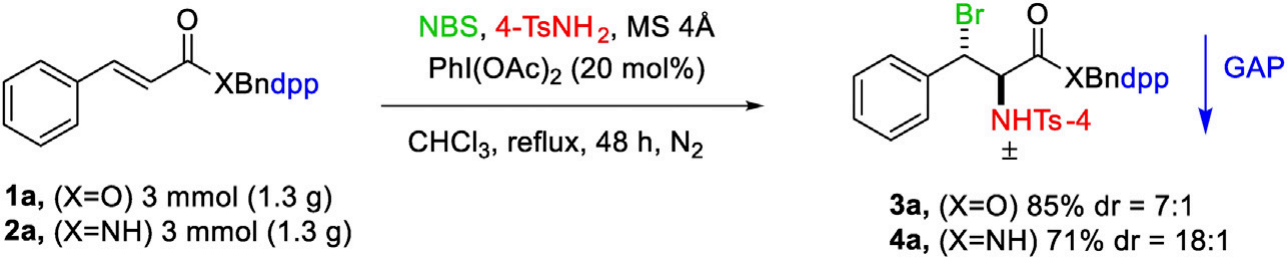
Gram scale reactions.

**SCHEME 2:**
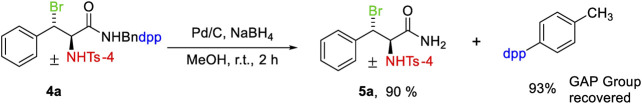
GAP deprotection.

In the presence of Pd/C and NaBH_4_, the GAP-tailored vicinal aminobromine was deprotected which afforded Bndpp in 93% yield (Schemes 1,2). The mixture is dissolved in a small volume of a solvent, such as ethyl acetate or DCM, and then petroleum ether is used to purify the products. The GAP auxiliary precipitates as a white solid that is filtered and treated with petroleum ether. To achieve the desired β-bromoamine as a white substance, the filtrate is evaporated under a vacuum.

### Mechanism

**SCHEME 3:**
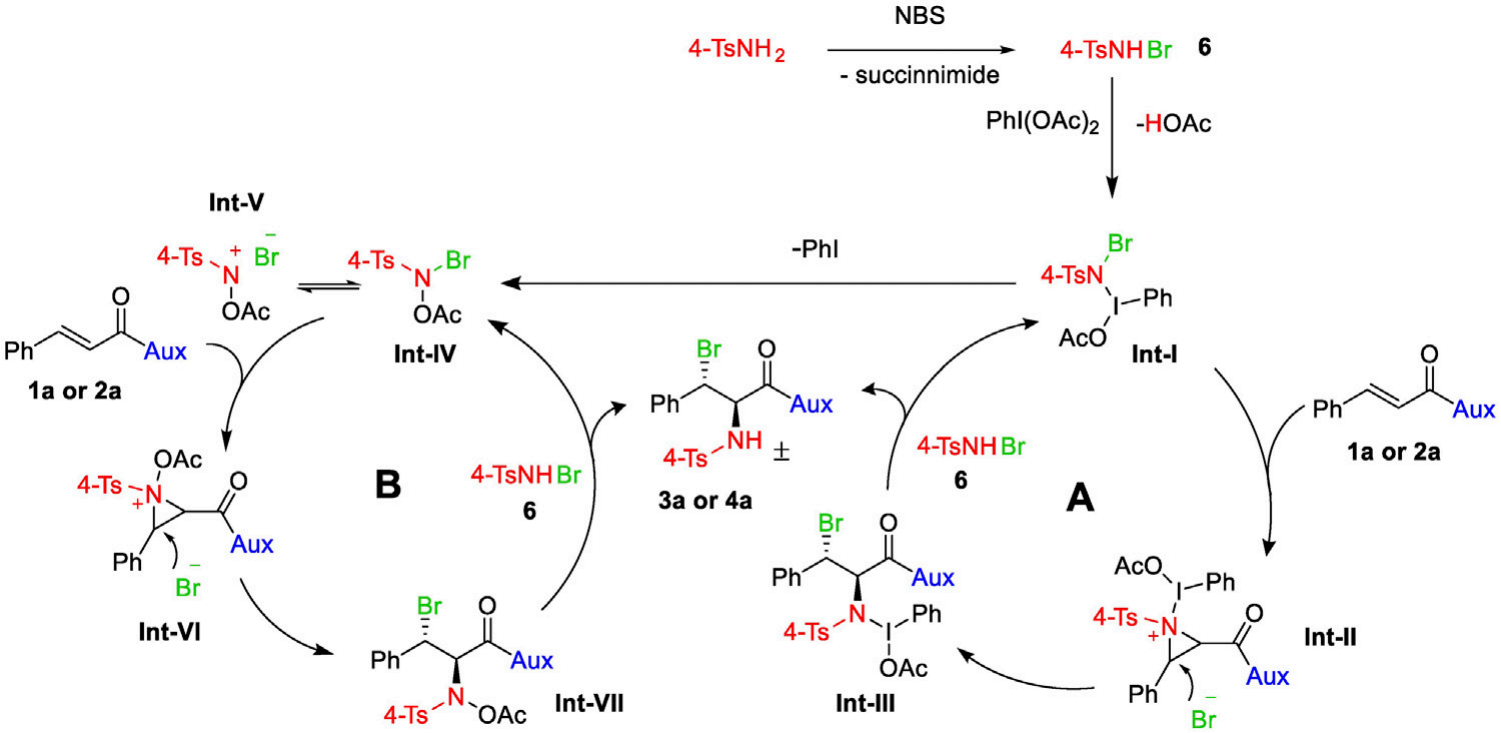
A possible pathway for the synthesis of vicinal bromoamines.

The outcomes of various experimentation within our research team, as well as other (Li et al., 2001; Wei et al., 2001; Wang and Wu, 2007; Wu and Wang, 2008; Chen et al., 2009a), lead to the conclusion that NBS may react with 4-TsNH_2_ to generate N-bromo-p-toluenesulfonamide (4-TsNHBr) 6 (Scheme 3), which would be oxidized by PhI(OAc)_2_ to generate intermediate Int-I that may either follow cycle A or cycle B. In cycle A, the Int-I will form aziridinium Int-II with a double bond of 1a or 2a, which is then stereoselectively attacked by the dissociated bromide from the Int-I at the more electrophilic carbon (beta to carbonyl carbon) to yield compound Int-III. Int-III and 16 eventually provide the ultimate bromoamine substance 3a or 4a and restore Int-I. When the fragile N–I bond of Int-I is broken, N-acetoxy-N-halo-p-toluenesulfonamide Int-IV can form, which could then be the active intermediate for cycle B. Int-IV that forms an equilibrium with nitrenium ion Int-V (Kikugawa et al., 2003; Murata et al., 2008) could react with olefin 1a or 2a to afford aziridinium Int-VI which would lead to Int-VII following an S_N_2 nucleophilic attack by the nearby bromide. Finally, the reaction of the intermediate Int-VII with 6 gives the final product and regenerates Int-IV.

Benefiting from the present methodology and this mechanism analysis, the utilizations of GAP chemistry for aminohalogenation and diamination of a broader scope of substrates (Chen et al., 2003b; Chen et al., 2004), in search for new chirality (Wu et al., 2019a; Wu et al., 2019b; Liu et al., 2020) and on multi-component reactions will be further conducted in our labs (Jiang et al., 2012a; Jiang et al., 2012b).

## Experimental Section

### Aminobromination of 4-(Diphenylphosphoryl) Benzyl Cinnamates 1a-k and N-(4-(Diphenylphosphoryl) Benzyl) Cinnamamides 2a-k

Typical procedure: Into a dry vial was added 1a or 2a (1 mmol, 1 eq), NBS (356 mg, 2 mmol, 2 eq), 4-TsNH_2_ (342 mg, 2 mmol, 2 eq), PhI(OAc)_2_ (64 mg, 20 mol%) and freshly activated 4 Å molecular sieves (500 mg) and capped under nitrogen protection. CHCl_3_ (3 ml) was added via a syringe and the reaction mixture was allowed to reflux for 48 h. After completion (monitored by TLC), the reaction was quenched with dropwise addition of saturated aqueous Na_2_SO_3_ solution (2 ml) and DCM (3 × 10 ml) was added to extract the product. The combined organic layers were washed with brine, dried over anhydrous sodium sulfate, and concentrated under reduced pressure. The mixture was redissolved in the minimal amount of solvents like ethyl acetate or DCM, and then petroleum ether was added. The GAP auxiliary precipitated in the form of a white solid which was filtered and washed with petroleum ether. The filtrate is evaporated under a vacuum to obtain the desired β-aminobromine as a white product.

### General Procedure for Deprotection of Group-Assisted Purification Auxiliary BnDpp.

To a 10 ml round bottom flask was added 4a (0.2 g, 0.32 mmol), 10 wt% Pd/C (20 mg) 2 ml MeOH and NaBH_4_ (15.2 mg, 2 equiv.). To prevent the loss of produced hydrogen and overpressure in the flask, it was sealed with a rubber septum and a deflated balloon. the reaction mixture was drained through a Celite after 2 h and the filtrate was concentrated under reduced pressure before being redissolved in EtOAc. After that, KHSO_4_ was used to neutralize the reaction mixture. The organic layer was separated, dried over anhydrous Na_2_SO_4_ and evaporated to dryness to afford crude GAP auxiliary, which was easily purified using the GAP washing method.

## Conclusion

In conclusion, we have demonstrated a new method for the preparation of vicinal aminobrominated products of electron-deficient olefins coupled with GAP auxiliaries dppBnOH and dppBnNH_2_. Good yields and diastereoselectivities were obtained in a clean and eco-friendly reaction condition comprising the catalyst PhI(OAc)_2_ with NBS and 4-TsNH_2_ as the bromine and nitrogen sources. The Group-Assisted Purification (GAP) chemistry was successfully applied and the compounds were obtained as precipitates without column chromatography and recrystallization by merely adding ethyl acetate and petroleum ether. Besides, the GAP auxiliary can be recovered for reuse.

## Data Availability

The original contributions presented in the study are included in the article/Supplementary Material, further inquiries can be directed to the corresponding authors.

## References

[B1] Albone D. P. Aujla P. S. Paul C. Taylor* S. Challenger S. Derrick A. M. (1998). A simple copper catalyst for both aziridination of alkenes and amination of activated hydrocarbons with chloramine-T trihydrate. J. Org. Chem. 63, 9569–9571. 10.1021/jo981335q

[B2] Alix A. Lalli C. Retailleau P. Masson G. (2012). Highly Enantioselective Electrophilic α-Bromination of Enecarbamates: Chiral Phosphoric Acid and Calcium Phosphate Salt Catalysts. J. Am. Chem. Soc. 134, 10389–10392. 10.1021/ja304095z 22686436

[B3] Ando T. Minakata S. Ryu I. Komatsu M. (1998). Nitrogen atom transfer to alkenes utilizing chloramine-T as a nitrogen source. Tetrahedron Lett. 39, 309–312. 10.1016/s0040-4039(97)10553-6

[B4] Cai Y. Liu X. Jiang J. Chen W. Lin L. Feng X. (2011). Catalytic Asymmetric Chloroamination Reaction of α,β-Unsaturated γ-Keto Esters and Chalcones. J. Am. Chem. Soc. 133, 5636–5639. 10.1021/ja110668c 21443187

[B5] Chen D. Guo L. Liu J. Kirtane S. Cannon J. F. Li G. (2005). Functionalization of α,β-Unsaturated Esters and Ketones: A Facile and Highly Stereoselective One-Pot Approach to N-Protected α,β-Dehydroamino Acid Derivatives. Org. Lett. 7, 921–924. 10.1021/ol050002u 15727475

[B6] Chen D. Kim S. H. Hodges B. Li G. (2003a). The cinnamate-based aminohalogenation provides an easy access to anti methyl 3-aryl-N-p-tosyl- and N-o-nosyl-aziridine-2-carboxylates. Arkivoc 2003, 56–63. 10.3998/ark.5550190.0004.c07

[B7] Chen D. Timmons C. Chao S. Li G. (2004). Regio- and Stereoselective Copper-Catalyzed Synthesis of Vicinal Haloamino Ketones fromα,β-Unsaturated Ketones. Eur. J. Org. Chem. 2004, 3097–3101. 10.1002/ejoc.200400050

[B65] Chen D. Timmons C. Wei H. X. Li G. (2003b). Direct Electrophilic Diamination of Functionalized Alkenes Without the Use of Any Metal Catalysts. J. Org. Chem. 68, 5742–5745. 10.1021/jo030098a 12839474

[B8] Chen S. Han J. Li G. Pan Y. (2013). Highly diastereoselective aminobromination of β-methyl-β-nitrostyrenes with t-butyl N,N-dibromocarbamate/t-butyl carbamate as bromine/nitrogen sources. Tetrahedron Lett. 54, 2781–2784. 10.1016/j.tetlet.2013.02.113

[B9] Chen Z.-G. Wang Y. Wei J.-F. Zhao P.-F. Shi X.-Y. (2010). K3PO4-Catalyzed Regiospecific Aminobromination of β-Nitrostyrene Derivatives with N-Bromoacetamide as Aminobrominating Agent. J. Org. Chem. 75, 2085–2088. 10.1021/jo9026879 20170090

[B10] Chen Z.-G. Wei J.-F. Li R.-T. Shi X.-Y. Zhao P.-F. (2009a). Copper Powder-Catalyzed Regio- and Stereoselective Aminobromination of α,β-Unsaturated Ketones with TsNH2 and NBS as Nitrogen and Halogen Sources. J. Org. Chem. 74, 1371–1373. 10.1021/jo8023768 19108612

[B11] Chen Z.-G. Wei J.-F. Wang M.-Z. Zhou L.-Y. Zhang C.-J. Shi X.-Y. (2009b). Aluminium Powder-Catalyzed Regio- and Stereoselective Aminobromination of α,β-Unsaturated Carbonyl Compounds and Simple Olefins with thep-Toluenesulfonamide/N-Bromosuccinimide (TsNH2-NBS) System. Adv. Synth. Catal. 351, 2358–2368. 10.1002/adsc.200900343

[B12] Chennapuram M. Emmadi N. R. Bingi C. Nanubolu J. B. Atmakur K. (2014). Group-assisted purification (GAP) chemistry for dihydrofurans: water as a medium for catalyst free synthesis in a one pot four component reaction. Green. Chem. 16, 3237–3246. 10.1039/C4GC00388H

[B13] Dommaraju Y. Prajapati D. (2015). A highly efficient group-assisted purification method for the synthesis of poly-functionalized pyrimidin-5-yl-pyrroles via one-pot four-component domino reaction. Mol. Divers. 19, 173–187. 10.1007/s11030-014-9547-1 25173493

[B14] Driguez H. Vermes J.-P. Lessard J. (1978). The chromous chloride promoted addition of N-haloamides to olefins. V. The addition of N-chloroamides to enol ethers: synthesis of acyloxy and acyl derivatives of α-amino acetals and ketals (aldehydes and ketones) and of 2-amino sugars. Can. J. Chem. 56, 119–130. 10.1139/v78-019

[B15] Fan R. Wen F. Qin L. Pu D. Wang B. (2007). PhI(OAc)2 induced intramolecular oxidative bromocyclization of homoallylic sulfonamides with KBr as the bromine source. Tetrahedron Lett. 48, 7444–7447. 10.1016/j.tetlet.2007.08.085

[B16] Fu H. Wang Z. Zhang Y. Jiang Y. Zhao Y. (2008). FeCl2-Catalyzed Aminobromination of Alkenes Using Amides or Sulfonamides and NBS as the Nitrogen and Bromine Sources. Synlett 2008, 2667–2670. 10.1055/s-0028-1083509

[B17] Gao G.-Y. Harden J. D. Zhang X. P. (2005). Cobalt-catalyzed efficient aziridination of alkenes. Org. Lett. 7, 3191–3193. 10.1021/ol050896i 16018618

[B18] Garrett C. E. Prasad K. (2004). The art of meeting palladium specifications in active pharmaceutical ingredients produced by Pd-catalyzed reactions. Adv. Synth. Catal. 346, 889–900. 10.1002/adsc.200404071

[B19] Ghorai M. K. Sahoo A. K. Kumar S. (2011). Synthetic route to chiral tetrahydroquinoxalines via ring-opening of activated aziridines. Org. Lett. 13, 5972–5975. 10.1021/ol2023906 22004011

[B20] Huang D. Wang H. Xue F. Guan H. Li L. Peng X. (2011). Enantioselective bromocyclization of olefins catalyzed by chiral phosphoric acid. Org. Lett. 13, 6350–6353. 10.1021/ol202527g 22092000

[B21] Huang R. Shaughnessy K. H. (2006). Water-soluble palladacycles as precursors to highly recyclable catalysts for the Suzuki coupling of aryl bromides in aqueous solvents. Organometallics 25, 4105–4112. 10.1021/om050940y

[B22] Jiang B. Feng B.-M. Wang S.-L. Tu S.-J. Li G. (2012a). Domino constructions of pentacyclic indeno[2,1-*c*]quinolines and pyrano[4,3-*b*]oxepines by [4+1]/[3+2+1]/[5+1] and [4+3] multiple cyclizations. Chem. Eur. J. 18, 9823–9826. 10.1002/chem.201201109 22767331 PMC3543830

[B23] Jiang B. Li Q.-Y. Tu S.-J. Li G. (2012b). Three-component domino reactions for selective formation of indeno[1,2-*b*]indole derivatives. Org. Lett. 14, 5210–5213. 10.1021/ol3023038 23025431

[B24] Kharasch M. S. Priestley H. M. (1939). The addition of N-haloamides to olefins. J. Am. Chem. Soc. 61, 3425–3432. 10.1021/ja01267a057

[B25] Kikugawa Y. Nagashima A. Sakamoto T. Miyazawa E. Shiiya M. (2003). Intramolecular Cyclization with Nitrenium Ions Generated by Treatment ofN-Acylaminophthalimides with Hypervalent Iodine Compounds: Formation of Lactams and Spiro-Fused Lactams. J. Org. Chem. 68, 6739–6744. 10.1021/jo0347009 12919042

[B26] Klepacz A. Zwierzak A. (2001). t-Butyl N,N-dibromocarbamate (BBC)-new reagent for aminobromination of terminal alkenes. Tetrahedron Lett. 42, 4539–4540. 10.1016/s0040-4039(01)00662-1

[B27] Li G. Kotti S. R. S. S. Timmons C. (2007). Recent Development of Regio‐ and Stereoselective Aminohalogenation Reaction of Alkenes. Eur. J. Org. Chem. 2007, 2745–2758. 10.1002/ejoc.200600990

[B28] Li G. Wei H.-X. Kim S. H. (2000). Copper-Catalyzed Aminohalogenation Using the 2-NsNCl2/2-NsNHNa Combination as the Nitrogen and Halogen Sources for the Synthesis of anti-alkyl 3-Chloro-2-(o-nitrobenzenesulfonamido)-3-arylpropionates. Org. Lett. 2, 2249–2252. 10.1021/ol000120b 10930255

[B29] Li G. Wei H.-X. Kim S. H. Neighbors M. (1999). Transition metal-catalyzed regioselective and stereoselective aminochlorination of cinnamic esters. Org. Lett. 1, 395–398. 10.1021/ol990059e

[B30] Li G. Wei H.-X. Kim S. H. (2001). Unexpected copper-catalyzed aminohalogenation reaction of olefins using *N*-halo-*N*-metallo-sulfonamide as the nitrogen and halogen sources. Tetrahedron 57, 8407–8411. 10.1016/S0040-4020(01)00847-X

[B31] Li H. Chao J. Hasan J. Tian G. Jin Y. Zhang Z. (2020a). Synthesis of tri(4-formylphenyl) phosphonate derivatives as recyclable triple-equivalent supports of peptide synthesis. J. Org. Chem. 85, 6271–6280. 10.1021/acs.joc.9b03023 32320241

[B32] Li H. Chao J. Tian G. Hasan J. Jin Y. Zhang Z. (2020b). Resin-free peptide synthesis mediated by tri(4-benzoylphenyl) phosphate (TBP) derivatives as small-molecule supports. Org. Chem. Front. 7, 689–696. 10.1039/C9QO01480B

[B33] Li H. Ren J. Li J. Zhang Z. Chang N. Qin C. (2020c). Greener liquid-phase synthesis and the ACE inhibitory structure-activity relationship of an anti-SARS octapeptide. Org. Biomol. Chem. 18, 8433–8442. 10.1039/D0OB01948H 33057549

[B34] Ling R. Yoshida M. Mariano P. S. (1996). Exploratory Investigations Probing a Preparatively Versatile, Pyridinium Salt Photoelectrocyclization−Solvolytic Aziridine Ring Opening Sequence. J. Org. Chem. 61, 4439–4449. 10.1021/jo960316i 11667350

[B35] Liu Y. Wu G. Yang Z. Rouh H. Katakam N. Ahmed S. (2020). Multi-layer 3D chirality: New synthesis, AIE and computational studies. Sci. China Chem. 63, 692–698. 10.1007/s11426-019-9711-x

[B36] Patel D. M. Vala R. M. Sharma M. G. Rajani D. P. Patel H. M. (2019). A Practical Green Visit to the Functionalized [1,2,4]Triazolo[5,1-b ]quinazolin-8(4H )one Scaffolds Using the Group-Assisted Purification (GAP) Chemistry and Their Pharmacological Testing. ChemistrySelect 4, 1031–1041. 10.1002/slct.201803605

[B37] Ponsold K. Ihn W. (1970). Die addition von cyanamid und halogen an olefine ein neues verfahren zur darstellung von vic.-halogencyanaminen und aziridinen. Tetrahedron Lett. 11, 1125–1128. 10.1016/s0040-4039(01)97925-0 5439242

[B38] Raghavan S. Ramakrishna Reddy S. Tony K. A. Naveen Kumar C. Nanda S. (2001). Bromosulfonamidation of alkenes using *S,S*-dimethyl-*N*-(*p*-toluenesulfonyl)sulfilimine. Synlett 2001, 0851–0853. 10.1055/s-2001-14910

[B39] Rahman A. U. Zarshad N. Zhou P. Yang W. Li G. Ali A. (2020). Hypervalent iodine (III) catalyzed regio- and diastereoselective aminochlorination of tailored electron deficient olefins via GAP chemistry. Front. Chem. 8, 1–9. 10.3389/fchem.2020.00523 32733847 PMC7358771

[B40] Saito S. Kikugawa Y. Murata K. Tsukamoto M. Sakamoto T. (2008). Hydrazidohydroxylation of styrenes with *N*-acetylaminophthalimide using Phenyliodine(III) bis(trifluoroacetate) (PIFA). Synthesis 2008, 32–38. 10.1055/s-2007-1000819

[B41] Schröder S. P. Van De Sande J. W. Kallemeijn W. W. Kuo C.-L. Artola M. Van Rooden E. J. (2017). Towards broad spectrum activity-based glycosidase probes: synthesis and evaluation of deoxygenated cyclophellitol aziridines. Chem. Commun. 53, 12528–12531. 10.1039/C7CC07730K 29116266

[B42] Seifert C. W. Paniagua A. White G. A. Cai L. Li G. (2016). GAP Peptide Synthesis through the Design of a GAP Protecting Group: An Fmoc/tBu Synthesis of Thymopentin Free from Polymers, Chromatography and Recrystallization. Eur. J. Org. Chem. 2016, 1714–1719. 10.1002/ejoc.201600026 PMC5486986 28663711

[B43] Shaikh T. M. Karabal P. U. Suryavanshi G. Sudalai A. (2009). Titanium superoxide: a heterogeneous catalyst for anti-markovnikov aminobromination of olefins. Tetrahedron Lett. 50, 2815–2817. 10.1016/j.tetlet.2009.03.169

[B44] Shi D. Ji S. Shi D. Ni S. Yang F. (2008). An efficient and green synthesis of 3,3′-benzylidenebis (4-hydroxy-6-methylpyridin-2(1h)-one) derivatives through multi-component reaction in ionic liquid. J. Heterocycl. Chem. 45, 1275–1280. 10.1002/jhet.5570450503

[B45] Śliwińska A. Zwierzak A. (2003). Ionic addition of *t*-butyl *N,N*-dibromocarbamate (BBC) to alkenes and cycloalkenes. Tetrahedron Lett. 44, 9323–9325. 10.1016/j.tetlet.2003.10.040

[B46] Thakur R. Rawal G. K. Vankar Y. D. (2017). Synthesis of Chiral Aziridines from Glycals: Application in the Synthesis of a Piperidine-Azepine Fused Derivative. Eur. J. Org. Chem. 2017, 4235–4241. 10.1002/ejoc.201700624

[B47] Thakur V. V. Talluri S. K. Sudalai A. (2003). Transition Metal-Catalyzed Regio- and Stereoselective Aminobromination of Olefins with TsNH2 and NBS as Nitrogen and Bromine Sources. Org. Lett. 5, 861–864. 10.1021/ol027530f 12633091

[B48] Van T. N. De Kimpe N. (2000). Synthesis of chiral *cis*-1,2,3-trisubstituted aziridines. Tetrahcdron 56, 7299–7304. 10.1016/S0040-4020(00)00627-X

[B49] Wang G.-W. Wu X.-L. (2007). Mechanochemical aminochlorination of electron-deficient olefins with chloramine-T promoted by (diacetoxyiodo)benzene. Adv. Synth. Catal. 349, 1977–1982. 10.1002/adsc.200700020

[B50] Wang H. Liu X. Feng X. Huang Z. Shi D. (2013). GAP chemistry for pyrrolyl coumarin derivatives: a highly efficient one-pot synthesis under catalyst-free conditions. Green. Chem. 15, 3307–3311. 10.1039/C3GC41799A

[B51] Wei H.-X. Kim S. H. Li G. (2001). The first transition metal-ligand complex-catalyzed regioselective and stereoselective aminohalogenation of cinnamic esters. Tetrahedron 57, 3869–3873. 10.1016/S0040-4020(01)00228-9

[B52] Wei J.-F. Chen Z.-G. Lei W. Zhang L.-H. Wang M.-Z. Shi X.-Y. (2009a). Silicon Powder: The First Nonmetal Elemental Catalyst for Aminobromination of Olefins with TsNH2 and NBS. Org. Lett. 11, 4216–4219. 10.1021/ol9015833 19681580

[B53] Wei J.-F. Zhang L.-H. Chen Z.-G. Shi X.-Y. Cao J.-J. (2009b). KI-catalyzed aminobromination of olefins with TsNH2-NBS combination. Org. Biomol. Chem. 7, 3280–3284. 10.1039/B904789A 19641786

[B54] Wu G. Liu Y. Yang Z. Jiang T. Katakam N. Rouh H. (2019a). Enantioselective assembly of multi-layer 3D chirality. Natl. Sci. Rev. 7, 588–599. 10.1093/nsr/nwz203 PMC8289020 34692078

[B55] Wu G. Liu Y. Yang Z. Katakam N. Rouh H. Ahmed S. (2019b). Multilayer *3D* chirality and its synthetic assembly. Research 2019, 1–11. 10.34133/2019/6717104 PMC6750085 31549078

[B56] Wu X.-L. Wang G.-W. (2007). Aminochlorination in Water: First Brønsted Acid-Promoted Synthesis of Vicinal Chloramines. J. Org. Chem. 72, 9398–9401. 10.1021/jo701957t 17973433

[B57] Wu X.-L. Wang G.-W. (2008). Aminohalogenation of electron-deficient olefins promoted by hypervalent iodine compounds. Eur. J. Org. Chem. 2008, 6239–6246. 10.1002/ejoc.200800842

[B58] Wu X.-L. Xia J.-J. Wang G.-W. (2008). Aminobromination of olefins with TsNH2 and NBS as the nitrogen and bromine sources mediated by hypervalent iodine in a ball mill. Org. Biomol. Chem. 6, 548–553. 10.1039/B717333D 18219426

[B59] Xie W. Jiang G. Liu H. Hu J. Pan X. Zhang H. (2013). Highly Enantioselective Bromocyclization of Tryptamines and its Application in the Synthesis of (−)-Chimonanthine. Angew. Chem. Int. Ed. 52, 12924–12927. 10.1002/anie.201306774 24123660

[B60] Xu X. Kotti S. R. S. S. Liu J. Cannon J. F. Headley A. D. Li G. (2004). Ionic liquid media resulted in the first asymmetric aminohalogenation reaction of alkenes. Org. Lett. 6, 4881–4884. 10.1021/ol048045i 15606090

[B61] Yadav J. S. Subba Reddy B. V. Narasimha Chary D. Chandrakanth D. (2009). InX3-catalyzed haloamidation of vinyl arenes: a facile synthesis of α-bromo- and α-fluoroamides. Tetrahedron Lett. 50, 1136–1138. 10.1016/j.tetlet.2008.12.090

[B62] Yeung Y. Gao X. Gao E. J. (2006a). A general process for the haloamidation of olefins. Scope and mechanism. J. Am. Chem. Soc. 128, 9644–9645. 10.1021/ja063675w 16866514

[B63] Yeung Y. Hong S. Hong E. J. (2006b). A short enantioselective pathway for the synthesis of the anti-influenza neuramidase inhibitor oseltamivir from 1,3-butadiene and acrylic acid. J. Am. Chem. Soc. 128, 6310–6311. 10.1021/ja0616433 16683783

[B64] Yu W. Z. Cheng Y. A. Wong M. W. Yeung Y.-Y. (2017). Atmosphere- and temperature-controlled regioselective aminobromination of olefins. Adv. Synth. Catal. 359, 234–239. 10.1002/adsc.201600821

