# Supplementary material for: Regio- and Diastereoselective Vicinal Aminobromination of Electron Deficient Olefins via Phosphorus-Based GAP Protocol
Source: Front Chem. 2021 Sep 8;9:742399. doi: 10.3389/fchem.2021.742399 (PMC8456122; doi:10.3389/fchem.2021.742399)
Supplement: Supplementary file 1 [file DataSheet1.docx]

***Supporting Information***

Regio- and Diastereoselective Vicinal Aminobromination of Electron Deficient Olefin Via Phosphorus based GAP Protocol

Anis Ur Rahman,^1^ Nighat Zarshad,^2^ Iltaf Khan,^3^ Fozia,^4^ Guigen Li *^1,5^ and Asad Ali *^6^

^1^ Institute of Chemistry and BioMedical Sciences, School of Chemistry and Chemical Engineering, Nanjing University, Nanjing, 210093, China

^2^ School of Chemistry and Chemical Engineering, Southeast University, Nanjing, 211189, China

^3^ Key Laboratory of Functional Inorganic Material Chemistry, School of Chemistry and Materials Science, Heilongjiang University, Harbin, China

^4^ Biochemistry Department, KMU Institute of Medical Sciences, 26000 Kohat, Pakistan

^5^ Department of Chemistry and Biochemistry, Texas Tech University, Lubbock, Texas 79409-1061, United States

^6^ Faculty of Chemical and Life Sciences, Department of Chemistry, Abdul Wali Khan University, Mardan, 23200, Pakistan.

*** Correspondence:**Corresponding Author
[guigen.li@ttu.edu](mailto:guigen.li@ttu.edu); [asad_org@yahoo.com](mailto:asad_org@yahoo.com)

**Table of Contents**

[1. General Methods 2](#_Toc76844358)

[2. General procedures for the preparation of starting materials 3](#_Toc76844359)

[2.1. Synthesis and characterization of GAP auxiliary (4-(hydroxymethyl)phenyl) diphenylphosphine oxide (dppBnOH) (4) 3](#_Toc76844360)

[2.2. Synthesis and characterization of GAP auxiliary (4-(aminomethyl)phenyl)diphenyl phosphine oxide (dppBnNH_2_) (9) 5](#_Toc76844361)

[2.3. General procedure for the preparation of 4-(diphenylphosphoryl)benzyl cinnamates (11a-k) 7](#_Toc76844362)

[2.4. General procedure for the preparation of 4-(diphenylphosphoryl)benzyl cinnamides (12a-k) 12](#_Toc76844363)

[3. Aminobromination of 4-(diphenylphosphoryl)benzyl cinnamates (13a-k) 18](#_Toc76844364)

[4. Aminobromination of *N*-(4-(diphenylphosphoryl)benzyl)cinnamamides (14a-k) 25](#_Toc76844365)

[5. General procedure for deprotection of GAP auxiliary BnDpp 32](#_Toc76844366)

[6. NMR Spectra 34](#_Toc76844367)

[7. References 72](#_Toc76844368)

# General Methods

Unless otherwise stated, all reactions were carried out under nitrogen atmosphere in dry Schlenk flask. All the reagents and solvents were purchased from commercial sources and used without additional purification. Reactions were monitored by thin layer chromatography (TLC) with a suitable solvent system on plates (GF254) visualized by using UV light (254 nm). NMR was recorded on Bruker Avance 400 (^1^H: 400 MHz, ^13^C: 100 MHz, ^31^P: 162 MHz). Chemical shifts were quoted in parts per million (ppm) referenced to the appropriate solvent peak CDCl_3_, DMSO-*d*_6_ or TMS. The following abbreviations were used to describe peak splitting patterns when appropriate: s (singlet), d (doublet), t (triplet), q (quartet), dd (doublet of doublet), ddd (doublet of doublet of doublet), m (multiplet). Coupling constants, *J*, were reported in hertz unit (Hz).

# General procedures for the preparation of starting materials

## Synthesis and characterization of GAP auxiliary (4-(hydroxymethyl)phenyl) diphenylphosphine oxide (dppBnOH) (4)

**Scheme S-1:** General procedure for the synthesis of dppBnOH **(4).**

*4-(diphenylphosphoryl)benzoic acid* ***(2)***

Following a procedure by Cole W. Seifert et al., (Seifert et al., 2016) diphenyl(*p*-tolyl)phosphane **1** (10 g, 36 mmol) was placed in a 500 mL round-bottomed flask, followed by NaOH (aq) (0.43 M, 130 mL) and then KMnO_4_ (22.2 g, 140 mmol). The reaction was stirred and heated at reflux for 12 h, after which the reaction mixture was filtered through Celite while hot. The resulting solution was washed twice with diethyl ether, followed by the addition of 50 % H_2_SO_4_ to precipitate the product. After filtration, 4-(diphenylphosphoryl)benzoic acid **2** (10.8 g, 93 %) was collected as a white solid that was directly subjected to the next reaction.

*Ethyl 4-(diphenylphosphoryl)benzoate* ***(3)***(Jablonkai and Keglevich, 2015)

4-(diphenylphosphoryl)benzoic acid **2** (10.6 g, 33 mmol) was placed in a 500 mL round-bottomed flask along with EtOH (300 mL) and SOCl_2_ (3 mL). The reaction was heated to reflux and stirred for 12 h. Upon completion, the reaction was cooled to room temperature, the solvent was evaporated and the residue obtained was purified by column chromatography (DCM/MeOH 3%) to give ethyl 4-(diphenylphosphoryl)benzoate **3** (9.0 g, 78 %) as a dense oil.

*(4-(hydroxymethyl)phenyl)diphenylphosphine oxide* ***(4)***(Seifert et al., 2016)

Ester **3** (9.0 g, 25.7mmol) was placed in a 500 mL round-bottomed flask along with ethanol (300 mL). The reaction was cooled to 0 °C, after which NaBH_4_ (2.9 g, 77 mmol) was added portion wise. The reaction was brought to room temperature and stirred for 12 h. The solvent was evacuated, followed by solvation of the crude material in DCM and washing three times with 2 M HCl (aq). The organic layer was then dried with Na_2_SO_4_, filtered, and evacuated to afford (4-(hydroxymethyl)phenyl)diphenylphosphine oxide (BndppOH) **4** as a white solid which was subsequently washed with ether to remove ester impurities. Yield 86% (6.8 g). ^1^H NMR (400 MHz, CDCl_3_) δ 7.60 – 7.51 (m, 4H), 7.51 – 7.29 (m, 10H), 5.08 (t, *J* = 6.0 Hz, 1H), 4.64 (d, *J* = 5.3 Hz, 2H). ^13^C NMR (101 MHz, CDCl_3_) δ 146.74, 146.71, 132.74, 132.05, 132.03, 132.00, 131.95, 131.94, 131.70, 130.59, 129.54, 128.58, 128.45, 126.65, 126.53, 63.89.

## Synthesis and characterization of GAP auxiliary (4-(aminomethyl)phenyl)diphenyl phosphine oxide (dppBnNH_2_) (9)

**Scheme S 2:** General procedure for the synthesis of dppBnNH_2_ **(9).**

*4-(diphenylphosphanyl)benzonitrile (****7****)* (Hingst et al., 1998)

Diphenylphosphane **5** (10.5 mL, 60 mmol) was added to a suspension of KOH (6 g, 108 mmol) in DMSO (300 mL) and the mixture were stirred for 2 h at RT. After addition of 4‑chlorobenzonitrile **6** (8.25 g, 60 mmol) the mixture was stirred for 1 h at 60 °C. Crude product was precipitated from the reaction mixture by addition of water and separated by filtration. Further purification was performed by recrystallization from MeOH to give white crystals 80% (13.8 g). ^1^H NMR (400 MHz, CDCl_3_) δ 7.57 (dd, *J* = 8.2, 1.5 Hz, 2H), 7.44 – 7.27 (m, 12H). ^31^P NMR (162 MHz, CDCl_3_) δ -4.28.

*(4-(diphenylphosphanyl)phenyl)methanamine (****8)***

Following the procedure of Janssen, M et al.,(Janssen et al., 2009) to a solution of phosphinobenzonitrile **7** (13.8 g, 48 mmol) in THF (300 mL) at 0°C was added LiAlH_4_ (7.3 g, 192 mmol) in small portions. The mixture was stirred for 2h at 50°C and after cooling to 0 °C, diluted with diethyl ether (150 mL). The reaction mixture was then quenched by careful addition of water and 15% aqueous sodium hydroxide (10 mL). The resultant slurry was filtered through a Celite pad, the phases in the filtrate were separated; the organic phase was dried over Na_2_SO_4_ and concentrated to dryness. The crude product was purified via column chromatography over silica using CHCl_3_/MeOH (9:1) as an eluent to afford yellowish oil 87% (12.2 g). ^1^H NMR (400 MHz, CDCl_3_) δ 7.35 – 7.28 (m, 12H), 3.87 (s, 2H), 1.71 (s, 2H). ^31^P NMR (162 MHz, CDCl_3_) δ -6.04. ^13^C NMR (101 MHz, CDCl_3_) δ 143.96, 137.45, 137.34, 135.53, 135.42, 134.23, 134.03, 133.80, 133.60, 128.71, 128.56, 128.52, 128.49, 127.36, 127.29, 46.21.

*(4-(aminomethyl)phenyl)diphenylphosphine oxide* ***(9)*** (Janssen et al., 2009)

The phosphinobenzylamine (12.2 g, 42 mmol) was dissolved in 250 mL of CH_2_Cl_2_ and H_2_O_2_ (17 mL, 35% solution in water) was added at 0°C. After 1h 40 mL of water was added, and the phases were separated. The organic layer was washed with saturated sodium bicarbonate solution, dried over Na_2_SO_4_ and concentrated to dryness. ^1^H NMR (400 MHz, CDCl_3_) δ 7.70 – 7.55 (m, 6H), 7.54 – 7.45 (m, 2H), 7.45 – 7.35 (m, 6H), 3.88 (s, 2H), 1.83 (s, 2H). ^31^P NMR (162 MHz, CDCl_3_) δ 29.01. ^13^C NMR (101 MHz, CDCl_3_) δ 147.41, 147.38, 133.17, 132.45, 132.35, 132.12, 132.02, 131.96, 131.93, 131.25, 130.20, 128.58, 128.46, 127.28, 127.15, 46.12.

## General procedure for the preparation of 4-(diphenylphosphoryl)benzyl cinnamates (11a-k)

BndppOH **4** (1.85 g, 1.6 mmol, 1 eq), cinnamic acida **10a-k** (1.76 mmol, 1.1 eq), and dry DCM (30 mL) were stirred at 0 °C in a 50 mL Schlenck flask. EDCI(HCl) (0.34 g, 1.76 mmol, 1.1 eq) was added, and the reaction was stirred for 10 min, at which point DMAP (20 mg, 10 mol%) was added, the reaction was brought to room temperature and stirred for 12 h. The reaction mixture was washed twice with satd. NH_4_Cl (aq), followed by two washes with satd. Na_2_CO_3_ (aq). The combined organic layers were dried with Na_2_SO_4_, filtered, and evacuated to afford the crude protected cinnamic acid. GAP purification was performed by dissolving the crude mixture in a minimal amount of ethyl acetate, followed by precipitation with petroleum ether and filtration of the resulting precipitate.

*4-(diphenylphosphoryl)benzyl cinnamate* ***(11a)***

White solid; 89% (624 mg); ^1^H NMR (400 MHz, CDCl_3_) δ 7.76 – 7.60 (m, 7H), 7.57 – 7.40 (m, 10H), 7.40 – 7.32 (m, 3H), 6.48 (d, *J* = 16.0 Hz, 1H), 5.29 (s, 2H). ^31^P NMR (162 MHz, CDCl_3_) δ 28.75. ^13^C NMR (101 MHz, CDCl_3_) δ 166.67, 145.73, 140.30, 140.27, 134.25, 133.01, 132.55, 132.45, 132.19, 132.09, 131.97, 130.60, 129.01, 128.68, 128.56, 128.23, 127.97, 127.85, 117.50, 65.57. HRMS (ESI) Calculated for C_28_H_23_O_3_P [M+Na]^+^: 461.1283 Found: 461.1285

*4-(diphenylphosphoryl)benzyl-2-methylcinnamate* ***(11b)***

White solid; 65% (470 mg); ^1^H NMR (400 MHz, CDCl_3_) δ 8.00 (d, *J* = 15.8 Hz, 1H), 7.72 – 7.59 (m, 6H), 7.53 – 7.36 (m, 9H), 7.24 – 7.09 (m, 3H), 6.38 (d, *J* = 15.9 Hz, 1H), 5.26 (s, 2H), 2.36 (s, 3H). ^31^P NMR (162 MHz, CDCl_3_) δ 28.54. ^13^C NMR (101 MHz, CDCl_3_) δ 166.17, 142.86, 139.87, 137.30, 132.68, 132.56, 132.02, 131.92, 131.67, 131.60, 131.57, 131.52, 130.42, 129.84, 128.18, 128.06, 127.46, 127.33, 126.01, 125.96, 117.97, 65.01, 19.34. HRMS (ESI) Calculated for C_29_H_25_O_3_P [M+Na]^+^: 475.1439 Found: 475.1444.

*4-(diphenylphosphoryl)benzyl-4-methylcinnamate* ***(11c)***

White solid; 66% (477 mg); ^1^H NMR (400 MHz, CDCl_3_) δ 7.74 – 7.60 (m, 7H), 7.55 – 7.35 (m, 10H), 7.15 (d, *J* = 7.9 Hz, 2H), 6.42 (d, *J* = 16.0 Hz, 1H), 5.26 (s, 2H), 2.32 (s, 3H). ^31^P NMR (162 MHz, CDCl_3_) δ 28.68. ^13^C NMR (101 MHz, CDCl_3_) δ 166.71, 145.61, 140.95, 140.35, 132.94, 132.89, 132.43, 132.32, 132.08, 132.05, 132.00, 131.98, 131.90, 131.85, 131.45, 129.64, 128.58, 128.46, 128.14, 127.85, 127.73, 116.30, 65.36, 21.46. HRMS (ESI) Calculated for C_29_H_25_O_3_P [M+Na]^+^: 475.1439 Found: 475.1444.

*4-(diphenylphosphoryl)benzyl-2-methoxycinnamate* ***(11d)***

White solid; 68% (509 mg); ^1^H NMR (400 MHz, CDCl_3_) δ 8.04 (d, *J* = 16.2 Hz, 1H), 7.67 (dt, *J* = 13.5, 7.8 Hz, 6H), 7.58 – 7.41 (m, 9H), 7.34 (t, *J* = 8.0 Hz, 1H), 6.99 – 6.86 (m, 2H), 6.58 (dd, *J* = 16.2, 2.7 Hz, 1H), 5.29 (s, 2H), 3.86 (d, *J* = 2.7 Hz, 3H).  ^31^P NMR (162 MHz, CDCl_3_) δ 28.95. ^13^C NMR (101 MHz, CDCl_3_) δ 167.22, 158.49, 141.22, 140.52, 132.96, 132.80, 132.49, 132.39, 132.18, 132.08, 131.92, 131.81, 129.19, 128.67, 128.55, 127.93, 127.81, 123.19, 120.78, 117.95, 111.22, 65.38, 55.55. HRMS (ESI) Calculated for C_29_H_25_O_4_P [M+Na]^+^: 491.1388 Found: 491.1393

*4-(diphenylphosphoryl)benzyl-3,4-dimethoxycinnamate* ***(11e)***

White solid; 63 % (502 mg); ^1^H NMR (400 MHz, CDCl_3_) δ 7.72 – 7.58 (m, 7H), 7.55 – 7.37 (m, 8H), 7.07 (d, *J* = 8.4 Hz, 1H), 7.01 (s, 1H), 6.82 (d, *J* = 7.8 Hz, 1H), 6.33 (dd, *J* = 16.0, 2.7 Hz, 1H), 5.25 (s, 2H), 3.86 (d, *J* = 2.7 Hz, 6H). ^31^P NMR (162 MHz, CDCl_3_) δ 28.80. ^13^C NMR (101 MHz, CDCl_3_) δ 166.82, 151.37, 149.28, 145.58, 140.43, 140.40, 132.94, 132.91, 132.47, 132.37, 132.13, 132.06, 132.03, 131.90, 131.87, 128.63, 128.51, 127.91, 127.79, 127.21, 122.84, 115.09, 111.12, 109.73, 65.39, 56.01, 55.93. HRMS (ESI) Calculated for C_30_H_27_O_5_P [M+Na]^+^: 521.1494 Found: 521.1501.

*4-(diphenylphosphoryl)benzyl-2,3,4-trimethoxycinnamate* ***(11f)***

White solid; 58% (490 mg); ^1^H NMR (400 MHz, CDCl_3_) δ 7.95 (d, *J* = 16.1 Hz, 1H), 7.78 – 7.61 (m, 6H), 7.59 – 7.40 (m, 8H), 7.27 (d, *J* = 9.3 Hz, 1H), 6.69 (d, *J* = 8.7 Hz, 1H), 6.48 (d, *J* = 16.2 Hz, 1H), 5.30 (s, 2H), 3.96 – 3.82 (m, 9H). ^31^P NMR (162 MHz, CDCl_3_) δ 28.85. ^13^C NMR (101 MHz, CDCl_3_) δ 167.16, 155.75, 153.39, 142.33, 140.68, 140.55, 132.93, 132.77, 132.44, 132.34, 132.13, 132.05, 132.03, 131.89, 131.73, 128.62, 128.50, 127.86, 127.74, 123.40, 121.23, 116.20, 107.62, 65.29, 61.47, 60.92, 56.09. HRMS (ESI) Calculated for C_31_H_29_O_6_P [M+Na]^+^: 551.1599 Found: 551.1602.

*4-(diphenylphosphoryl)benzyl-4-bromocinnamate* ***(11g)***

White solid; 78% (644 mg); ^1^H NMR (400 MHz, CDCl_3_) δ 7.66 (dd, *J* = 12.4, 8.6 Hz, 7H), 7.58 – 7.50 (m, 3H), 7.50 – 7.40 (m, 7H), 7.40 – 7.32 (m, 2H), 6.46 (dd, *J* = 16.2, 2.7 Hz, 1H), 5.28 (s, 2H). ^31^P NMR (162 MHz, CDCl_3_) δ 28.81. ^13^C NMR (101 MHz, CDCl_3_) δ 166.38, 144.29, 140.13, 140.10, 133.13, 132.91, 132.53, 132.43, 132.24, 132.16, 132.06, 131.87, 129.58, 128.67, 128.55, 127.99, 127.86, 124.87, 118.17, 65.67. HRMS (ESI) Calculated for C_28_H_22_BrO_3_P [M+Na]^+^: 539.0388 Found: 539.0385

*4-(diphenylphosphoryl)benzyl-3-fluorocinnamate* ***(11h)***

White solid; 68% (496 mg); ^1^H NMR (400 MHz, CDCl_3_) δ 7.72 – 7.57 (m, 7H), 7.54 – 7.36 (m, 8H), 7.33 – 7.20 (m, 2H), 7.16 (dt, *J* = 9.7, 2.0 Hz, 1H), 7.01 (td, *J* = 8.3, 2.5 Hz, 1H), 6.44 (d, *J* = 16.0 Hz, 1H), 5.26 (s, 2H). ^31^P NMR (162 MHz, CDCl_3_) δ 28.64. ^13^C NMR (101 MHz, CDCl_3_) δ 166.09, 164.13, 161.67, 144.10, 144.08, 140.05, 140.02, 136.40, 136.32, 133.00, 132.85, 132.42, 132.31, 132.04, 132.00, 131.97, 131.94, 131.81, 130.50, 130.42, 128.56, 128.44, 127.88, 127.76, 124.12, 124.09, 118.85, 117.40, 117.18, 114.40, 114.18, 65.56. HRMS (ESI) Calculated for C_28_H_22_FO_3_P [M+Na]^+^: 479.1188 Found: 479.1198.

*4-(diphenylphosphoryl)benzyl-4-bromo-2-fluorocinnamate* ***(11i)***

White solid; 81% (692 mg); ^1^H NMR (400 MHz, CDCl_3_) δ 8.03 (d, *J* = 16.0 Hz, 1H), 7.74 – 7.61 (m, 6H), 7.61 – 7.38 (m, 9H), 7.33 (dd, *J* = 8.1, 2.6 Hz, 1H), 7.03 (td, *J* = 8.3, 2.6 Hz, 1H), 6.37 (d, *J* = 15.9 Hz, 1H), 5.29 (s, 2H). ^31^P NMR (162 MHz, CDCl_3_) δ 29.04. ^13^C NMR (101 MHz, CDCl_3_) δ 165.72, 164.30, 161.76, 142.53, 140.02, 139.99, 132.99, 132.86, 132.37, 132.27, 132.00, 131.90, 130.54, 130.50, 129.00, 128.91, 128.52, 128.40, 127.80, 127.67, 125.67, 125.57, 120.67, 120.43, 120.02, 120.00, 115.39, 115.18, 65.54. HRMS (ESI) Calculated for C_28_H_21_BrFO_3_P [M+Na]^+^: 557.0293 Found: 557.0298

*4-(diphenylphosphoryl)benzyl-2-chloro-5-nitrocinnamate* ***(11j)***

White solid; 93% (769 mg); ^1^H NMR (400 MHz, CDCl_3_) δ 8.47 (d, *J* = 3.1 Hz, 1H), 8.20 – 8.00 (m, 2H), 7.79 – 7.35 (m, 15H), 6.62 (d, *J* = 15.9 Hz, 1H), 5.32 (s, 2H). ^31^P NMR (162 MHz, CDCl_3_) δ 28.81. ^13^C NMR (101 MHz, CDCl_3_) δ 165.39, 146.80, 141.25, 139.09, 134.03, 132.84, 132.58, 132.48, 132.15, 132.06, 131.80, 131.35, 128.68, 128.56, 128.07, 127.95, 125.28, 123.09, 122.61, 66.08. HRMS (ESI) Calculated for C_28_H_21_ClNO_5_P [M+Na]^+^: 540.0744 Found: 540.0745.

*4-(diphenylphosphoryl)benzyl-3-(naphthalen-1-yl)acrylate* ***(11k)***

White solid; 72% (562 mg); ^1^H NMR (400 MHz, CDCl_3_) δ 8.58 (dd, *J* = 15.7, 3.0 Hz, 1H), 8.15 (d, *J* = 8.3 Hz, 1H), 7.86 (t, *J* = 9.4 Hz, 2H), 7.78 – 7.60 (m, 7H), 7.60 – 7.37 (m, 11H), 6.57 (dd, *J* = 15.7, 3.0 Hz, 1H), 5.34 (s, 2H). ^31^P NMR (162 MHz, CDCl_3_) δ 28.85. ^13^C NMR (101 MHz, CDCl_3_) δ 166.49, 142.61, 140.25, 140.22, 133.64, 132.95, 132.87, 132.49, 132.39, 132.11, 132.06, 132.01, 131.92, 131.84, 131.44, 131.35, 130.79, 128.77, 128.62, 128.50, 127.96, 127.84, 126.98, 126.29, 125.45, 125.13, 123.24, 119.96, 65.58. HRMS (ESI) Calculated for C_32_H_25_O_3_P [M+Na]^+^: 511.1439 Found: 511.1447

## General procedure for the preparation of 4-(diphenylphosphoryl)benzyl cinnamides (12a-k)

To a stirred solution of 1.6 mmol cinnamic acid **10a-k** and dppBnNH_2_ (0.49 g, 1.6 mmol) in 30 mL anhydrous DCM is added DCC (0.33 g, 1.6 mmol) at room temperature, which is then stirred overnight. Precipitated urea is then filtered off and the filtrate evaporated down in vacuo. GAP purification was performed by dissolving the crude mixture in a minimal amount of ethyl acetate, followed by precipitation with petroleum ether and filtration of the resulting precipitate.

*N-(4-(diphenylphosphoryl)benzyl)cinnamamide* ***(12a)***

White solid; 92% (643mg); ^1^H NMR (400 MHz, CDCl_3_) δ 8.09 (t, *J* = 5.3 Hz, 1H), 7.63 (d, *J* = 15.7 Hz, 1H), 7.60 – 7.49 (m, 6H), 7.45 – 7.35 (m, 7H), 7.33 – 7.23 (m, 5H), 6.67 (d, *J* = 15.7 Hz, 1H), 4.57 (d, *J* = 5.9 Hz, 2H). ^31^P NMR (162 MHz, CDCl_3_) δ 29.83. ^13^C NMR (101 MHz, CDCl_3_) δ 166.57, 143.52, 143.49, 140.88, 135.20, 132.78, 132.41, 132.31, 132.25, 132.22, 132.11, 132.02, 131.74, 131.17, 130.12, 129.54, 128.80, 128.78, 128.66, 128.03, 127.90, 121.21, 43.28. HRMS (ESI) Calculated for C_28_H_24_NO_2_P [M+Na]^+^: 460.1442 Found: 460.1445.

*N-(4-(diphenylphosphoryl)benzyl)-2-methylcinnamamide* ***(12b)***

^1^H NMR (400 MHz, CDCl_3_) δ 8.46 (t, *J* = 5.9 Hz, 1H), 7.91 (d, *J* = 15.5 Hz, 1H), 7.53 (dddd, *J* = 11.4, 9.6, 7.7, 1.4 Hz, 6H), 7.43 – 7.27 (m, 9H), 7.23 – 7.09 (m, 2H), 7.02 (td, *J* = 7.5, 1.6 Hz, 1H), 6.64 (d, *J* = 15.6 Hz, 1H), 4.56 (d, *J* = 5.8 Hz, 2H), 2.37 (s, 3H). ^31^P NMR (162 MHz, CDCl_3_) δ 29.96. ^13^C NMR (101 MHz, CDCl_3_) δ 166.70, 143.63, 143.61, 138.37, 137.47, 134.19, 132.67, 132.29, 132.19, 132.03, 131.93, 131.63, 130.92, 130.67, 129.87, 129.19, 128.72, 128.60, 128.00, 127.88, 126.19, 126.10, 122.40, 43.19, 19.88. HRMS (ESI) Calculated for C_29_H_26_NO_2_P [M+Na]^+^: 474.1599 Found: 474.1599.

*N-(4-(diphenylphosphoryl)benzyl)-2-methoxycinnamamide* ***(12c)***

White solid; 96% (718 mg); ^1^H NMR (400 MHz, CDCl_3_) δ 8.17 (t, *J* = 6.0 Hz, 1H), 7.92 (d, *J* = 15.8 Hz, 1H), 7.59 – 7.43 (m, 6H), 7.42 – 7.18 (m, 10H), 6.84 – 6.70 (m, 3H), 4.51 (d, *J* = 5.9 Hz, 2H), 3.70 (s, 3H). ^31^P NMR (162 MHz, CDCl_3_) δ 29.69. ^13^C NMR (101 MHz, CDCl_3_) δ 167.09, 158.11, 143.67, 143.64, 136.00, 132.74, 132.21, 132.10, 132.08, 132.06, 131.99, 131.89, 131.70, 130.94, 130.57, 129.89, 128.63, 128.51, 127.84, 127.72, 124.09, 121.80, 120.53, 111.06, 55.35, 43.09. HRMS (ESI) Calculated for C_29_H_26_NO_3_P [M+Na]^+^: 490.1548 Found: 490.1552.

*N-(4-(diphenylphosphoryl)benzyl)-3,4-dimethoxycinnamamide* ***(12d)***

White solid; 96% (764 mg); ^1^H NMR (400 MHz, CDCl_3_) δ 8.06 (t, *J* = 6.0 Hz, 1H), 7.62 – 7.46 (m, 7H), 7.43 – 7.32 (m, 6H), 7.31 – 7.24 (m, 2H), 7.02 – 6.88 (m, 2H), 6.76 (d, *J* = 8.2 Hz, 1H), 6.58 (d, *J* = 15.6 Hz, 1H), 4.55 (d, *J* = 5.8 Hz, 2H), 3.86 (s, 3H), 3.69 (s, 3H). ^31^P NMR (162 MHz, CDCl_3_) δ 29.76. ^13^C NMR (101 MHz, CDCl_3_) δ 166.81, 150.46, 149.10, 143.64, 140.61, 132.80, 132.31, 132.21, 132.18, 132.04, 131.94, 131.09, 130.04, 128.73, 128.61, 128.23, 127.90, 127.78, 122.02, 119.22, 111.12, 109.78, 56.01, 55.78, 43.16. HRMS (ESI) Calculated for C_30_H_28_NO_4_P [M+Na]^+^: 520.1654 Found: 520.1661.

*N-(4-(diphenylphosphoryl)benzyl)-2,3,4-trimethoxycinnamamide* ***(12e)***

White solid; 96% (810 mg); ^1^H NMR (400 MHz, CDCl_3_) δ 8.10 (t, *J* = 6.0 Hz, 1H), 7.78 (d, *J* = 15.8 Hz, 1H), 7.59 – 7.45 (m, 6H), 7.43 – 7.33 (m, 6H), 7.28 (dd, *J* = 8.4, 2.8 Hz, 2H), 7.07 (d, *J* = 8.8 Hz, 1H), 6.66 (d, *J* = 15.8 Hz, 1H), 6.52 (d, *J* = 8.7 Hz, 1H), 4.51 (d, *J* = 5.9 Hz, 2H), 3.85 – 3.72 (m, 9H). ^31^P NMR (162 MHz, CDCl_3_) δ 29.77. ^13^C NMR (101 MHz, CDCl_3_) δ 167.08, 154.77, 153.04, 143.69, 143.66, 142.34, 135.68, 132.71, 132.22, 132.12, 132.10, 132.07, 132.00, 131.90, 131.67, 130.92, 129.88, 128.63, 128.51, 127.81, 127.68, 122.99, 122.17, 120.32, 107.51, 61.27, 60.85, 56.03, 43.08. HRMS (ESI) Calculated for C_31_H_30_NO_5_P [M+Na]^+^: 550.1759 Found: 550.1766.

*N-(4-(diphenylphosphoryl)benzyl)-4-bromocinnamamide* ***(12f)***

White solid; 91% (750 mg); ^1^H NMR (400 MHz, CDCl_3_) δ 8.47 (t, *J* = 5.9 Hz, 1H), 7.59 – 7.48 (m, 7H), 7.45 – 7.31 (m, 8H), 7.31 – 7.16 (m, 4H), 6.70 (d, *J* = 15.5 Hz, 1H), 4.56 (d, *J* = 5.8 Hz, 2H). ^31^P NMR (162 MHz, CDCl_3_) δ 30.19. ^13^C NMR (101 MHz, CDCl_3_) δ 166.34, 143.53, 139.31, 134.19, 132.61, 132.34, 132.24, 132.05, 131.95, 131.56, 130.91, 129.86, 129.27, 128.81, 128.69, 128.06, 127.93, 123.49, 122.07, 43.24. HRMS (ESI) Calculated for C_28_H_23_BrNO_2_P [M+Na]^+^: 538.0547 Found: 538.0541.

*N-(4-(diphenylphosphoryl)benzyl)-3-fluorocinnamamide* ***(12g)***

White solid; 89% (648 mg); ^1^H NMR (400 MHz, CDCl_3_) δ 8.55 (t, *J* = 6.0 Hz, 1H), 7.64 – 7.47 (m, 7H), 7.46 – 7.17 (m, 9H), 7.12 (d, *J* = 7.7 Hz, 1H), 7.06 (dt, *J* = 9.9, 2.0 Hz, 1H), 6.97 (td, *J* = 8.3, 2.6 Hz, 1H), 6.72 (d, *J* = 15.7 Hz, 1H), 4.57 (d, *J* = 5.8 Hz, 2H). ^31^P NMR (162 MHz, CDCl_3_) δ 30.06. ^13^C NMR (101 MHz, CDCl_3_) δ 166.24, 164.23, 161.78, 143.52, 139.27, 137.64, 137.56, 132.63, 132.34, 132.31, 132.24, 132.03, 131.93, 131.58, 130.32, 130.24, 128.82, 128.70, 128.10, 127.98, 124.07, 122.81, 116.39, 116.17, 113.96, 113.74, 43.26. HRMS (ESI) Calculated for C_28_H_23_FNO_2_P [M+Na]^+^: 478.1348 Found: 478.1348.

*N-(4-(diphenylphosphoryl)benzyl)-4-bromo-2-fluorocinnamamide* ***(12h)***

White solid; 88% (751 mg); ^1^H NMR (400 MHz, CDCl_3_) δ 8.63 (t, *J* = 5.9 Hz, 1H), 7.88 (d, *J* = 15.6 Hz, 1H), 7.59 – 7.45 (m, 6H), 7.42 – 7.22 (m, 10H), 6.82 (td, *J* = 8.3, 2.6 Hz, 1H), 6.64 (d, *J* = 15.6 Hz, 1H), 4.55 (d, *J* = 5.9 Hz, 2H). ^31^P NMR (162 MHz, CDCl_3_) δ 30.12. ^13^C NMR (101 MHz, CDCl_3_) δ 165.95, 163.81, 161.29, 143.56, 137.83, 132.61, 132.29, 132.20, 132.03, 131.93, 131.79, 131.76, 131.57, 130.89, 129.84, 128.77, 128.65, 128.08, 127.96, 125.39, 125.30, 124.29, 124.27, 120.58, 120.34, 115.07, 114.85, 43.24. HRMS (ESI) Calculated for C_28_H_22_BrFNO_2_P [M+H]^+^: 534.0634 Found: 534.0637.

*3-(2-chloro-5-nitrophenyl)-N-(4-(diphenylphosphoryl)benzyl)acrylamide* ***(12i)***

^1^H NMR (400 MHz, CDCl_3_) δ 8.39 (t, *J* = 5.9 Hz, 1H), 8.07 (dd, *J* = 9.0, 2.7 Hz, 1H), 7.68 – 7.30 (m, 17H), 6.72 (d, *J* = 15.6 Hz, 1H), 4.60 (d, *J* = 5.8 Hz, 2H). ^31^P NMR (162 MHz, CDCl_3_) δ 29.90. ^13^C NMR (101 MHz, CDCl_3_) δ 165.26, 146.76, 143.32, 143.29, 141.12, 135.31, 134.31, 132.50, 132.39, 132.35, 132.29, 132.05, 131.95, 131.46, 131.10, 128.82, 128.70, 128.19, 128.06, 127.17, 124.25, 122.34, 43.34. HRMS (ESI) Calculated for C_28_H_22_ClN_2_O_4_P [M+Na]^+^: 539.0903 Found: 539.0909.

*N-(4-(diphenylphosphoryl)benzyl)-4-chloro-3-nitrocinnamamide* ***(12j)***

White solid; 96% (793 mg); ^1^H NMR (400 MHz, CDCl_3_) δ 8.91 (t, *J* = 5.8 Hz, 1H), 7.81 (s, 1H), 7.62 – 7.22 (m, 17H), 6.85 (d, *J* = 15.7 Hz, 1H), 4.57 (d, *J* = 5.7 Hz, 2H). ^31^P NMR (162 MHz, CDCl_3_) δ 30.18. ^13^C NMR (101 MHz, CDCl_3_) δ 165.52, 148.25, 143.38, 136.53, 135.61, 132.44, 132.33, 132.23, 132.06, 132.00, 131.90, 131.39, 130.92, 129.87, 128.86, 128.74, 128.13, 128.01, 127.81, 127.13, 125.15, 123.70, 43.27. HRMS (ESI) Calculated for C_28_H_22_ClN_2_O_4_P [M+Na]^+^: 539.0903 Found: 539.0906.

*N-(4-(diphenylphosphoryl)benzyl)-3-(naphthalen-1-yl)acrylamide* ***(12k)***

^1^H NMR (400 MHz, CDCl_3_) δ 8.52 (t, *J* = 6.0 Hz, 1H), 8.46 (d, *J* = 15.5 Hz, 1H), 8.20 (dd, *J* = 9.2, 2.5 Hz, 1H), 7.86 – 7.73 (m, 2H), 7.57 – 7.27 (m, 18H), 6.77 (d, *J* = 15.4 Hz, 1H), 4.59 (d, *J* = 5.8 Hz, 2H). ^31^P NMR (162 MHz, CDCl_3_) δ 29.97. ^13^C NMR (101 MHz, CDCl_3_) δ 166.56, 143.62, 143.59, 137.72, 133.69, 132.78, 132.67, 132.33, 132.23, 132.20, 132.17, 132.04, 131.94, 131.63, 131.58, 130.99, 129.95, 129.65, 128.72, 128.59, 128.03, 127.90, 126.63, 126.12, 125.43, 124.60, 124.23, 123.85, 43.24. HRMS (ESI) Calculated for C_32_H_26_NO_2_P [M+Na]^+^: 510.1599 Found: 510.1601.

# Aminobromination of 4-(diphenylphosphoryl)benzyl cinnamates (13a-k)

**Typical procedure:** Into a dry vial was added **11a** (1 mmol, 1 eq), NBS (356 mg, 2 mmol, 2 eq), 4-TsNH_2_ (342 mg, 2 mmol, 2 eq), PhI(OAc)_2_ (64 mg, 20 mol%) and freshly activated 4 Å molecular sieves (500 mg) and capped under nitrogen protection. CHCl_3_ (3 mL) was added via a syringe and the reaction mixture was allowed to reflux for 48 h. After completion (monitored by TLC), the reaction was quenched with dropwise addition of saturated aqueous Na_2_SO_3_ solution (2 mL) and DCM (3 × 10 mL) was added to extract the product. The combined organic layers were washed with brine, dried over anhydrous sodium sulfate, and concentrated under reduced pressure. The mixture was redissolved in the minimal amount of solvents like ethyl acetate or DCM, and then petroleum ether was added. The GAP auxiliary precipitated in the form of a white solid which was filtered and washed with petroleum ether. The filtrate is evaporated under a vacuum to obtain the desired β-aminobromine as a white product.

*4-(diphenylphosphoryl)benzyl 3-bromo-2-((4-methylphenyl)sulfonamido)-3-phenylpropanoate* ***(13a)***

White solid, 94% (193.8 mg); ^1^H NMR (400 MHz, CDCl_3_) δ 7.73 – 7.39 (m, 16H), 7.36 – 7.07 (m, 7H), 6.17 (d, *J* = 9.8 Hz, 1H), 5.12 (d, *J* = 7.8 Hz, 1H), 5.06 – 4.86 (m, 2H), 4.57 (t, *J* = 8.8 Hz, 1H), 2.34 (s, 3H). ^31^P NMR (162 MHz, CDCl_3_) δ 28.57. ^13^C NMR (101 MHz, CDCl_3_) δ 168.79, 143.67, 138.61, 138.59, 136.73, 136.35, 133.40, 132.80, 132.44, 132.37, 132.34, 132.18, 132.15, 132.05, 131.76, 129.57, 129.05, 128.71, 128.66, 128.59, 128.55, 128.36, 128.32, 128.22, 128.09, 127.96, 127.27, 127.14, 66.87, 61.92, 51.41, 21.58. HRMS (ESI) Calculated for C_35_H_31_BrFNO_5_PS [M+H]^+^: 688.0922 Found: 688.0920

*4-(diphenylphosphoryl)benzyl 3-bromo-2-((4-methylphenyl)sulfonamido)-3-(o-tolyl)propanoate* ***(13b)***

White solid, 85% (178.8 mg); ^1^H NMR (400 MHz, CDCl_3_) δ 7.69 – 7.38 (m, 15H), 7.38 – 7.23 (m, 3H), 7.18 – 6.97 (m, 4H), 6.73 (d, *J* = 9.7 Hz, 1H), 5.37 (d, *J* = 9.2 Hz, 1H), 5.06 – 4.81 (m, 2H), 4.68 (t, *J* = 9.6 Hz, 1H), 2.32 (s, 3H), 2.22 (s, 3H). ^31^P NMR (162 MHz, CDCl_3_) δ 28.70. ^13^C NMR (101 MHz, CDCl_3_) δ 169.39, 143.53, 138.73, 138.70, 136.74, 136.20, 134.69, 133.10, 132.72, 132.35, 132.25, 132.10, 132.00, 131.68, 130.76, 130.47, 129.47, 128.82, 128.63, 128.51, 128.14, 128.02, 127.89, 127.71, 127.20, 126.63, 66.67, 60.46, 47.66, 21.50, 19.13. HRMS (ESI) Calculated for C_36_H_33_BrNO_5_PS [M+H]^+^: 702.1078 Found: 702.1076.

*4-(diphenylphosphoryl)benzyl 3-bromo-2-((4-methylphenyl)sulfonamido)-3-(p-tolyl)propanoate* ***(13c)***

White solid, 89% (187.2 mg); ^1^H NMR (400 MHz, CDCl_3_) δ 7.70 – 7.38 (m, 16H), 7.35 – 7.27 (m, 2H), 7.16 – 7.02 (m, 4H), 6.93 (d, *J* = 7.8 Hz, 1H), 5.14 – 4.83 (m, 3H), 4.54 (t, *J* = 8.9 Hz, 1H), 2.32 (s, 3H), 2.23 (s, 3H). ^31^P NMR (162 MHz, CDCl_3_) δ 28.74. ^13^C NMR (101 MHz, CDCl_3_) δ 168.92, 143.51, 139.01, 138.64, 136.72, 133.35, 132.67, 132.38, 132.28, 132.15, 132.11, 132.01, 131.64, 129.53, 129.45, 129.33, 129.14, 128.76, 128.68, 128.56, 128.14, 128.01, 127.21, 66.77, 61.89, 51.24, 21.57, 21.20. HRMS (ESI) Calculated for C_36_H_33_BrNO_5_PS [M+H]^+^: 702.1078 Found: 702.1074.

*4-(diphenylphosphoryl)benzyl 3-bromo-3-(2-methoxyphenyl)-2-((4-methylphenyl)sulfonamido)propanoate* ***(13d)***

White solid, 80% (172.1 mg); ^1^H NMR (400 MHz, CDCl_3_) δ 7.75 – 7.02 (m, 21H), 6.67 – 6.48 (m, 2H), 6.05 (dd, *J* = 17.4, 9.7 Hz, 1H), 5.22 – 4.87 (m, 2H), 4.77 – 4.60 (m, 1H), 3.72 (s, 3H), 2.31 (d, *J* = 6.5 Hz, 3H). ^31^P NMR (162 MHz, CDCl_3_) δ 28.57. ^13^C NMR (101 MHz, CDCl_3_) δ 168.47, 155.49, 143.40, 138.80, 137.29, 136.74, 133.86, 132.89, 132.73, 132.67, 132.47, 132.30, 132.24, 132.19, 132.14, 131.97, 131.87, 131.63, 130.15, 129.39, 129.34, 128.53, 128.41, 127.84, 127.72, 126.89, 126.62, 126.35, 112.84, 111.89, 66.56, 60.06, 55.85, 48.50, 21.46. HRMS (ESI) Calculated for C_36_H_33_BrNO_6_PS [M+H]^+^: 688.0922 Found: 688.0913.

*4-(diphenylphosphoryl)benzyl 3-bromo-3-(3,4-dimethoxyphenyl)-2-((4-methylphenyl)sulfonamido)propanoate* ***(13e)***

Light yellow solid, 85% (190.5 mg); ^1^H NMR (400 MHz, CDCl_3_) δ 7.76 – 7.19 (m, 18H), 7.05 – 6.92 (m, 2H), 6.82 (d, *J* = 14.2 Hz, 1H), 6.68 (d, *J* = 9.9 Hz, 1H), 5.32 (d, *J* = 8.2 Hz, 1H), 5.23 – 5.02 (m, 2H), 4.88 (t, *J* = 9.0 Hz, 1H), 3.76 (s, 3H), 3.59 (s, 3H), 2.28 (s, 3H). ^31^P NMR (162 MHz, CDCl_3_) δ 29.12. ^13^C NMR (101 MHz, CDCl_3_) δ 168.20, 149.41, 148.78, 142.76, 139.06, 137.83, 132.43, 132.32, 132.13, 132.01, 129.00, 128.96, 128.68, 128.56, 127.81, 127.68, 127.62, 127.50, 127.06, 120.32, 115.04, 110.28, 66.93, 60.28, 56.13, 55.85, 47.08, 21.37. HRMS (ESI) Calculated for C_37_H_35_BrNO_7_PS [M+H]^+^: 748.1133 Found: 748.1128.

*4-(diphenylphosphoryl)benzyl 3-bromo-2-((4-methylphenyl)sulfonamido)-3-(2,3,4-trimethoxyphenyl)propanoate* ***(13f)***

Light yellow solid, 87% (202.8 mg); ^1^H NMR (400 MHz, CDCl_3_) 7.85 – 7.03 (m, 19H), 6.88 (d, *J* = 11.6 Hz, 1H), 6.66 (d, *J* = 8.6 Hz, 1H), 5.45 – 4.81 (m, 3H), 4.52 (t, *J* = 9.1 Hz, 1H), 3.90 (s, 3H), 3.82 (s, 3H), 3.75 (s, 3H), 2.30 (s, 3H). ^31^P NMR (162 MHz, CDCl_3_) δ 28.91. ^13^C NMR (101 MHz, CDCl_3_) δ 167.86, 154.11, 150.50, 146.40, 142.97, 137.33, 132.72, 132.48, 132.38, 132.13, 132.03, 131.68, 129.17, 128.65, 128.53, 127.70, 127.06, 126.98, 110.93, 106.37, 67.04, 61.12, 60.91, 60.53, 55.93, 45.60, 21.51. HRMS (ESI) Calculated for C_38_H_37_BrNO_8_PS [M+ H]^+^: 778.1239 Found: 778.1237.

*4-(diphenylphosphoryl)benzyl 3-bromo-3-(4-bromophenyl)-2-((4-methylphenyl)sulfonamido)propanoate* ***(13g)***

White solid, 72% (165.2 mg); ^1^H NMR (400 MHz, CDCl_3_) δ 7.78 – 7.39 (m, 15H), 7.37 – 7.24 (m, 4H), 7.17 (d, *J* = 8.1 Hz, 2H), 7.09 (d, *J* = 8.5 Hz, 2H), 5.05 (dd, *J* = 12.9, 8.0 Hz, 2H), 4.97 (d, *J* = 13.0 Hz, 1H), 4.55 (t, *J* = 9.6 Hz, 1H), 2.34 (s, 3H). ^31^P NMR (162 MHz, CDCl_3_) δ 28.90. ^13^C NMR (101 MHz, CDCl_3_) δ 168.99, 143.46, 138.87, 138.85, 136.98, 135.88, 132.59, 132.39, 132.29, 132.20, 132.18, 132.11, 132.01, 131.57, 130.04, 129.43, 128.73, 128.70, 128.58, 128.08, 127.96, 126.96, 122.98, 66.74, 61.83, 49.79, 21.61. HRMS (ESI) Calculated for C_35_H_30_Br_2_NO_5_PS [M+H]^+^: 766.0027 Found: 766.0030.

*4-(diphenylphosphoryl)benzyl 3-bromo-3-(3-fluorophenyl)-2-((4-methylphenyl)sulfonamido)propanoate* ***(13h)***

White solid, 61% (129 mg); ^1^H NMR (400 MHz, CDCl_3_) δ 7.61 – 7.39 (m, 13H), 7.35 (dd, *J* = 8.2, 2.6 Hz, 2H), 7.26 (d, *J* = 9.7 Hz, 1H), 7.13 – 7.04 (m, 4H), 7.00 (d, *J* = 8.3 Hz, 1H), 6.94 – 6.81 (m, 2H), 5.11 (d, *J* = 9.0 Hz, 1H), 5.09 – 4.89 (m, 2H), 4.56 (t, *J* = 9.4 Hz, 1H), 2.32 (s, 3H). ^31^P NMR (162 MHz, CDCl_3_) δ 28.92. ^13^C NMR (101 MHz, CDCl_3_) δ 168.77, 163.48, 161.03, 143.43, 139.12, 139.05, 138.74, 138.71, 136.80, 132.99, 132.59, 132.56, 132.05, 131.95, 131.49, 130.13, 130.04, 129.40, 128.64, 128.51, 128.06, 127.94, 126.97, 124.11, 124.08, 115.92, 115.71, 115.63, 115.40, 66.70, 61.81, 49.74, 21.44. HRMS (ESI) Calculated for C_35_H_29_Br_2_FNO_5_PS [M+H]^+^: 706.0828 Found: 706.0824.

*4-(diphenylphosphoryl)benzyl 3-bromo-3-(4-bromo-2-fluorophenyl)-2-((4-methylphenyl)sulfonamido)propanoate* ***(13i)***

White solid, 57% (133.9 mg); ^1^H NMR (400 MHz, CDCl_3_) δ 7.69 (m, 8H), 7.59 – 7.34 (m, 11H), 7.32 – 7.12 (m, 3H), 5.66 (d, *J* = 11.8 Hz, 1H), 5.47 – 5.25 (m, 2H), 5.00 (d, *J* = 11.8 Hz, 1H), 2.41 (s, 3H). ^31^P NMR (162 MHz, CDCl_3_) δ 28.66. ^13^C NMR (100 MHz CDCl_3_) δ 168.98, 157.97, 155.51, 142.51, 137.98, 132.58, 132.07, 131.85, 131.73, 131.45, 131.33, 130.04, 130.02, 129.87, 129.32, 128.77, 128.44, 128.01, 127.63, 126.38, 121.17, 119.27, 119.05, 66.63, 56.56, 56.45, 48.99, 21.52. HRMS (ESI) Calculated for C_35_H_29_Br_2_FNO_5_PS [M+H]^+^: 783.9933 Found: 783.9930.

*4-(diphenylphosphoryl)benzyl 3-bromo-3-(2-chloro-4-nitrophenyl)-2-((4-methylphenyl)sulfonamido)propanoate* ***(13j)***

White solid, 53% (121.8 mg); ^1^H NMR (400 MHz, CDCl_3_) δ 8.23 (d, *J* = 1.6 Hz, 1H), 8.08 (d, *J* = 8.5 Hz, 1H), 7.72 – 7.37 (m, 18H), 7.28 (d, *J* = 7.6 Hz, 2H), 5.61 (d, *J* = 6.9 Hz, 1H), 5.02 – 4.82 (m, 2H), 4.38 (t, *J* = 9.5 Hz, 1H), 2.40 (s, 3H). ^31^P NMR (162 MHz, CDCl_3_) δ 28.70. ^13^C NMR (101 MHz, CDCl_3_) δ 170.96, 147.27, 142.67, 137.74, 135.72, 131.87, 131.80, 131.58, 131.45, 130.42, 129.87, 129.40, 128.34, 127.63, 127.10, 125.41, 123.15, 66.68, 57.53, 49.55, 21.51. HRMS (ESI) Calculated for C_35_H_29_Cl_2_N_2_O_7_PS [M+Na]^+^: 745.0708 Found: 745.0703.

*4-(diphenylphosphoryl)benzyl-3-bromo-2-((4-methylphenyl)sulfonamido)-3-(naphthalen-1-yl)propanoate* ***(13k)***

White solid, 81% (179.1 mg); ^1^H NMR (400 MHz, CDCl_3_) δ 7.89 – 7.40 (m, 16H), 7.38 – 7.05 (m, 9H), 6.26 (d, *J* = 8.4 Hz, 1H), 5.37 (d, *J* = 7.0 Hz, 1H), 5.22 – 5.03 (m, 2H), 4.58 (t, *J* = 8.7 Hz, 1H), 2.37 (s, 3H). ^31^P NMR (162 MHz, CDCl_3_) δ 28.91. ^13^C NMR (101 MHz, CDCl_3_) δ 167.56, 142.37, 138.45, 136.54, 133.68, 132.79, 132.33, 132.26, 132.15, 132.12, 132.00, 131.72, 131.22, 130.14, 129.77, 129.60, 129.26, 129.18, 128.71, 128.63, 128.22, 127.89, 127.21, 126.72, 126.72, 126.06, 125.63, 122.48, 66.72, 56.79, 51.63, 21.47. HRMS (ESI) Calculated for C_39_H_33_BrNO_5_PS [M+Na]^+^: 760.0898 Found: 760.0891.

# Aminobromination of *N*-(4-(diphenylphosphoryl)benzyl)cinnamamides (14a-k)

**Typical procedure:** Into a dry vial was added **12a** (1 mmol, 1 eq), NBS (356 mg, 2 mmol, 2 eq), 4-TsNH_2_ (342 mg, 2 mmol, 2 eq), PhI(OAc)_2_ (64 mg, 20 mol%) and freshly activated 4 Å molecular sieves (500 mg) and capped under nitrogen protection. CHCl_3_ (3 mL) was added via a syringe and the reaction mixture was allowed to reflux for 48 h. After completion (monitored by TLC), the reaction was quenched with dropwise addition of saturated aqueous Na_2_SO_3_ solution (2 mL) and DCM (3 × 10 mL) was added to extract the product. The combined organic layers were washed with brine, dried over anhydrous sodium sulfate, and concentrated under reduced pressure. The mixture was redissolved in the minimal amount of solvents like ethyl acetate or DCM, and then petroleum ether was added. The GAP auxiliary precipitated in the form of a white solid which was filtered and washed with petroleum ether. The filtrate is evaporated under a vacuum to obtain the desired β-aminobromine as a white product.

*3-bromo-N-(4-(diphenylphosphoryl)benzyl)-2-((4-methylphenyl)sulfonamido)-3-phenylpropanamide* ***(14a)***

White solid, 78% (160.5 mg); ^1^H NMR (400 MHz, DMSO-*d*_6_) δ 8.91 (t, *J* = 5.8 Hz, 1H), 8.36 (d, *J* = 9.7 Hz, 1H), 7.66 – 7.48 (m, 12H), 7.47 – 7.41 (m, 2H), 7.38 – 7.29 (m, 4H), 7.29 – 7.23 (m, 3H), 7.17 (d, *J* = 7.9 Hz, 2H), 5.05 (d, *J* = 10.6 Hz, 1H), 4.60 (t, *J* = 10.2 Hz, 1H), 4.20 (dd, *J* = 15.9, 6.0 Hz, 1H), 4.03 (dd, *J* = 15.9, 5.3 Hz, 1H), 2.27 (s, 3H). ^31^P NMR (162 MHz, DMSO-*d*_6_) δ 25.40. ^13^C NMR (101 MHz, DMSO-*d*_6_) δ 168.56, 142.74, 142.71, 142.27, 138.24, 138.11, 133.32, 132.30, 132.04, 132.02, 131.50, 131.47, 131.40, 131.36, 130.53, 129.06, 128.81, 128.69, 128.47, 128.37, 128.28, 127.54, 127.42, 126.32, 60.54, 51.76, 42.08, 20.91. HRMS (ESI) Calculated for C_35_H_32_BrN_2_O_4_PS [M+Na]^+^: 709.0901 Found: 709.0892.

*3-bromo-N-(4-(diphenylphosphoryl)benzyl)-2-((4-methylphenyl)sulfonamido)-3-(o-tolyl)propanamide* ***(14b)***

White solid, 81% (170.1 mg); ^1^H NMR (400 MHz, DMSO-*d*_6_) δ 8.68 (d, *J* = 9.4 Hz, 1H), 8.60 (t, *J* = 6.0 Hz, 1H), 7.72 (d, *J* = 7.9 Hz, 2H), 7.67 – 7.49 (m, 11H), 7.31 (d, *J* = 8.0 Hz, 2H), 7.24 (dd, *J* = 11.6, 7.7 Hz, 2H), 7.09 (t, *J* = 7.5 Hz, 1H), 6.95 (q, *J* = 7.7 Hz, 2H), 6.57 – 6.48 (m, 2H), 5.38 (d, *J* = 10.3 Hz, 1H), 4.55 (t, *J* = 9.9 Hz, 1H), 3.88 (dd, *J* = 16.2, 6.9 Hz, 1H), 3.52 (dd, *J* = 16.3, 4.7 Hz, 1H), 2.32 (s, 3H), 2.19 (s, 3H). ^31^P NMR (162 MHz, DMSO-*d*_6_) δ 25.54. ^13^C NMR (101 MHz, DMSO-*d*_6_) δ 167.15, 142.44, 142.41, 142.39, 138.53, 136.15, 135.37, 133.27, 132.25, 132.05, 132.03, 131.47, 131.37, 131.29, 131.19, 131.02, 130.22, 129.99, 129.05, 128.78, 128.67, 128.40, 126.68, 126.57, 126.44, 61.99, 51.28, 41.38, 20.96, 18.66. HRMS (ESI) Calculated for C_36_H_34_BrN_2_O_4_PS [M+H]^+^: 701.1235 Found: 701.1238.

*3-bromo-N-(4-(diphenylphosphoryl)benzyl)-3-(2-methoxyphenyl)-2-((4-methylphenyl)sulfonamido)propenamide* ***(14c)***

White solid, 79% (169.7 mg); ^1^H NMR (400 MHz, DMSO-*d*_6_) δ 8.66 (t, *J* = 6.3 Hz, 1H), 7.97 (d, *J* = 7.9 Hz, 2H), 7.72 – 7.51 (m, 12H), 7.48 (d, *J* = 8.0 Hz, 2H), 7.36 (dd, *J* = 11.6, 7.8 Hz, 2H), 7.29 – 7.12 (m, 3H), 6.88 (d, *J* = 7.8 Hz, 2H), 6.81 (d, *J* = 8.7 Hz, 1H), 4.28 – 4.07 (m, 2H), 3.99 (d, *J* = 7.6 Hz, 1H), 3.67 (s, 3H), 2.38 (s, 3H). ^31^P NMR (162 MHz, DMSO-*d*_6_) δ 25.49. ^13^C NMR (101 MHz, DMSO-*d*_6_) δ 162.75, 157.03, 145.33, 143.01, 142.99, 133.28, 132.91, 132.26, 131.99, 131.97, 131.47, 131.37, 131.27, 131.22, 130.63, 130.04, 128.76, 128.64, 128.23, 126.71, 126.59, 122.05, 112.90, 111.45, 55.91, 43.83, 41.65, 41.47, 21.11. HRMS (ESI) Calculated for C_36_H_34_BrN_2_O_5_PS [M+H]^+^: 739.1007 Found: 739.1012.

*3-bromo-3-(3,4-dimethoxyphenyl)-N-(4-(diphenylphosphoryl)benzyl)-2-((4-methylphenyl)sulfonamido)propenamide* ***(14d)***

Light yellow solid, 80% (179.1 mg); ^1^H NMR (400 MHz, CDCl_3_) δ 7.77 (t, *J* = 6.0 Hz, 1H), 7.69 – 7.34 (m, 16H), 7.30 – 7.15 (m, 4H), 6.96 (s, 1H), 6.60 (s, 1H), 4.52 (t, *J* = 5.8 Hz, 2H), 4.26 (d, *J* = 4.3 Hz, 1H), 3.93 (d, *J* = 4.4 Hz, 1H), 3.85 (s, 3H), 3.63 (s, 3H), 2.35 (s, 3H). ^31^P NMR (162 MHz, CDCl_3_) δ 29.25. ^13^C NMR (101 MHz, CDCl_3_) δ 165.41, 150.25, 148.04, 144.77, 142.19, 136.25, 132.83, 132.53, 132.43, 132.19, 132.15, 132.05, 131.79, 129.66, 128.76, 128.63, 128.07, 127.68, 127.56, 116.37, 115.27, 112.23, 56.35, 55.95, 50.66, 45.76, 43.14, 21.71. HRMS (ESI) Calculated for C_37_H_36_BrN_2_O_6_PS [M+H]^+^: 747.1293 Found: 747.1294.

*3-bromo-N-(4-(diphenylphosphoryl)benzyl)-2-((4-methylphenyl)sulfonamido)-3-(2,3,4-trimethoxyphenyl)propenamide* ***(14e)***

Light yellow solid, 79% (183.9 mg); ^1^H NMR (400 MHz, CDCl_3_) δ 8.76 (t, *J* = 5.9 Hz, 1H), 7.72 – 7.12 (m, 19H), 7.01 (d, *J* = 7.9 Hz, 2H), 5.44 (d, *J* = 9.5 Hz, 1H), 4.72 (t, *J* = 9.6 Hz, 1H), 4.41 – 4.06 (m, 2H), 3.95 – 3.71 (m, 9H), 2.32 (s, 3H). ^31^P NMR (162 MHz, CDCl_3_) δ 29.77. ^13^C NMR (101 MHz, CDCl_3_) δ 169.24, 151.61, 151.32, 146.83, 143.43, 142.35, 136.78, 132.58, 132.40, 132.32, 132.30, 132.30, 132.24, 132.24, 132.19, 132.09, 131.53, 131.10, 129.70, 129.44, 128.87, 128.75, 128.03, 127.99, 127.87, 127.76, 127.42, 111.77, 61.69, 61.26, 61.08, 60.93, 44.78, 43.34, 21.72. HRMS (ESI) Calculated for C_38_H_38_BrN_2_O_7_PS [M+Na]^+^: 799.1218 Found: 799.1212.

*3-bromo-3-(4-bromophenyl)-N-(4-(diphenylphosphoryl)benzyl)-2-((4-methylphenyl)sulfonamido)propanamide* ***(14f)***

White solid, 69% (158.1 mg); ^1^H NMR (400 MHz, CDCl_3_) δ 9.22 (t, *J* = 6.1 Hz, 1H), 8.05 (d, *J* = 7.6 Hz, 1H), 7.60 – 7.28 (m, 22H), 4.98 (d, *J* = 10.6 Hz, 1H), 4.34 (d, *J* = 10.1 Hz, 1H), 4.16 (dd, *J* = 15.8, 5.9 Hz, 1H), 3.98 (dd, *J* = 15.8, 5.8 Hz, 1H), 2.30 (s, 3H). ^31^P NMR (162 MHz, CDCl_3_) δ 30.22. ^13^C NMR (100 MHz, CDCl_3_) δ 169.09, 142.51, 141.18, 137.98, 137.20, 132.53, 132.17, 132.07, 132.04, 131.92, 131.85, 131.73, 131.28, 130.02, 128.77, 128.44, 128.00, 127.79, 127.55, 126.54, 122.74, 56.96, 51.49, 44.09, 21.48. HRMS (ESI) Calculated for C_35_H_31_Br_2_N_2_O_4_PS [M+H]^+^: 765.0187 Found: 765.0188.

*3-bromo-N-(4-(diphenylphosphoryl)benzyl)-3-(3-fluorophenyl)-2-((4-methylphenyl)sulfonamido)propenamide* ***(14g)***

White solid, 67% (141.5 mg); ^1^H NMR (400 MHz, DMSO-*d*_6_) δ 8.92 (t, *J* = 5.7 Hz, 1H), 8.45 (d, *J* = 9.7 Hz, 1H), 7.68 – 7.41 (m, 14H), 7.39 – 7.02 (m, 8H), 5.06 (d, *J* = 10.5 Hz, 1H), 4.59 (t, *J* = 10.1 Hz, 1H), 4.26 (dd, *J* = 16.0, 5.9 Hz, 1H), 4.11 (dd, *J* = 15.9, 5.4 Hz, 1H), 2.27 (s, 3H). ^31^P NMR (162 MHz, DMSO-*d*_6_) δ 25.51. ^13^C NMR (101 MHz, DMSO-*d*_6_) δ 168.48, 162.81, 160.39, 142.72, 142.69, 142.37, 140.96, 140.89, 137.98, 133.28, 132.26, 132.05, 132.02, 131.58, 131.50, 131.41, 130.55, 130.24, 130.16, 129.08, 128.80, 128.69, 127.55, 127.43, 126.24, 124.74, 115.44, 115.25, 115.04, 60.52, 50.22, 42.12, 20.90. HRMS (ESI) Calculated for C_35_H_31_BrFN_2_O_4_PS [M+H]^+^: 705.0988 Found: 705.0986.

*3-bromo-3-(4-bromo-2-fluorophenyl)-N-(4-(diphenylphosphoryl)benzyl)-2-((4-methylphenyl)sulfonamido)propanamide* ***(14h)***

White solid, 66% (154.8 mg); ^1^H NMR (400 MHz, CDCl_3_) δ 9.19 (s, 1H), 7.71 – 7.36 (m, 14), 7.32 – 7.08 (m, 4H), 6.99 (d, *J* = 8.0 Hz, 2H), 6.62 (td, *J* = 8.3, 2.7 Hz, 1H), 6.29 (d, *J* = 10.1 Hz, 1H), 5.56 (d, *J* = 9.8 Hz, 1H), 5.00 (t, *J* = 10.0 Hz, 1H), 4.16 (dd, *J* = 15.5, 5.6 Hz, 1H), 3.98 (dd, *J* = 15.4, 4.7 Hz, 1H), 2.32 (s, 3H). ^31^P NMR (162 MHz, CDCl_3_) δ 30.28. ^13^C NMR (101 MHz, CDCl_3_) δ 168.81, 143.22, 142.33, 142.30, 137.10, 132.51, 132.50, 132.33, 132.22, 132.18, 132.07, 131.29, 130.77, 129.72, 129.71, 129.34, 128.93, 128.90, 128.81, 128.78, 128.36, 128.24, 127.98, 127.59, 127.54, 124.40, 124.30, 115.21, 61.80, 48.81, 43.50, 21.63. HRMS (ESI) Calculated for C_35_H_30_Br_2_FN_2_O_4_PS [M+H]^+^: 783.0093 Found: 783.0090.

*3-bromo-3-(2-chloro-4-nitrophenyl)-N-(4-(diphenylphosphoryl)benzyl)-2-((4-methylphenyl)sulfonamido)propanamide* ***(14i)***

White solid, 54% (124 mg); ^1^H NMR (400 MHz, CDCl_3_) δ 9.84 (t, *J* = 5.9 Hz, 1H), 8.28 (s, 1H), 8.06 (dd, *J* = 8.8, 2.5 Hz, 1H), 7.64 – 7.27 (m, 20H), 6.09 (d, *J* = 11.5 Hz, 1H), 5.61 (d, *J* = 11.6 Hz, 1H), 4.66 (dd, *J* = 15.7, 5.9 Hz, 1H) 4.50 (dd, *J* = 15.7, 5.3 Hz, 1H), 2.33 (s, 3H). ^31^P NMR (162 MHz, CDCl_3_) δ 30.58. ^13^C NMR (101 MHz, CDCl_3_) δ 167.18, 146.84, 142.65, 142.62, 140.69, 138.53, 132.34, 132.22, 132.12, 132.04, 131.91, 131.81, 130.99, 130.82, 129.76, 129.48, 128.79, 128.67, 126.32, 124.39, 124.08, 60.42, 47.31, 43.31, 21.05. HRMS (ESI) Calculated for C_35_H_30_BrClN_3_O_6_PS [M+H]^+^: 766.0543 Found: 766.0547.

*3-bromo-3-(4-chloro-3-nitrophenyl)-N-(4-(diphenylphosphoryl)benzyl)-2-((4-methylphenyl)sulfonamido)propanamide* ***(14j)***

White solid, 56% (128.5 mg); ^1^H NMR (400 MHz, CDCl_3_) δ 8.95 (t, *J* = 5.8 Hz, 1H), 8.29 (s, 1H), 7.70 – 7.17 (m, 19H), 7.00 (d, *J* = 8.1 Hz, 2H), 4.73 (d, *J* = 7.9 Hz, 1H), 4.59 – 4.26 (m, 2H), 4.13 (d, *J* = 7.3 Hz, 1H), 2.33 (s, 3H). ^31^P NMR (162 MHz, CDCl_3_) δ 31.62. ^13^C NMR (101 MHz, CDCl_3_) δ 170.21, 148.21, 144.45, 142.87, 138.17, 137.59, 133.84, 132.53, 132.29, 132.07, 132.00, 131.68, 130.89, 129.53, 129.45, 129.22, 128.77, 128.55, 128.35, 127.98, 127.54, 127.17, 127.09, 125.27, 61.59, 51.27, 43.09, 21.54. HRMS (ESI) Calculated for C_35_H_30_BrClN_3_O_6_PS [M+H]^+^: 766.0543 Found: 766.0542.

*3-bromo-N-(4-(diphenylphosphoryl)benzyl)-2-((4-methylphenyl)sulfonamido)-3-(naphthalen-1-yl)propanamide* ***(14k)***

White solid, 77% (170 mg); ^1^H NMR (400 MHz, CDCl_3_) δ 9.86 (t, *J* = 5.8 Hz, 1H), 8.20 (d, *J* = 8.4 Hz, 1H), 7.89 – 7.72 (m, 2H), 7.68 – 7.31 (m, 22H), 7.20 (t, *J* = 7.7 Hz, 1H), 6.52 (d, *J* = 11.4 Hz, 1H), 5.83 (d, *J* = 11.4 Hz, 1H), 4.71 (dd, *J* = 15.6, 6.2 Hz, 1H) 4.52 (dd, *J* = 15.7, 5.2 Hz, 1H), 2.28 (s, 3H). ^31^P NMR (162 MHz, CDCl_3_) δ 30.41. ^13^C NMR (101 MHz, CDCl_3_) δ 168.42, 143.03, 143.01, 134.19, 133.71, 132.32, 132.29, 132.18, 132.02, 131.92, 131.25, 130.89, 130.61, 129.50, 128.87, 128.82, 128.70, 127.97, 127.84, 126.87, 126.10, 125.31, 122.76, 48.40, 46.07, 43.34, 22.06. HRMS (ESI) Calculated for C_39_H_34_BrN_2_O_4_PS [M+H]^+^: 737.1238 Found: 737.1230.

# General procedure for deprotection of GAP auxiliary BnDpp

To a 10 mL round bottom flask was added **14a** (0.2 g, 0.32 mmol), 10 wt% Pd/C (20 mg) 2 mL MeOH and NaBH_4_ (15.2 mg, 2 equiv.). To prevent the loss of produced hydrogen and overpressure in the flask, it was sealed with a rubber septum and a deflated balloon. the reaction mixture was drained through a Celite after 2 h and the filtrate was concentrated under reduced pressure before being redissolved in EtOAc. After that, KHSO_4_ was used to neutralize the reaction mixture. The organic layer was separated, dried over anhydrous Na_2_SO_4_ and evaporated to dryness to afford crude GAP auxiliary, which was easily purified using the GAP washing method.

*3-bromo-2-((4-methylphenyl)sulfonamido)-3-phenylpropanamide* ***(15a)***

^1^H NMR (400 MHz, DMSO-*d*_6_) δ 8.12 (d, *J* = 9.8 Hz, 1H), 7.79 – 7.69 (m, 1H), 7.63 (d, *J* = 8.0 Hz, 1H), 7.48 – 7.10 (m, 9H), 4.97 (d, *J* = 9.8 Hz, 1H), 4.35 (t, *J* = 9.8 Hz, 1H), 2.34 (s, 3H). ^13^C NMR (101 MHz, DMSO-*d*_6_) δ 170.00, 142.20, 138.26, 137.77, 129.11, 128.42, 128.25, 128.21, 126.34, 60.87, 50.05, 21.03.

# NMR Spectra

**2a**


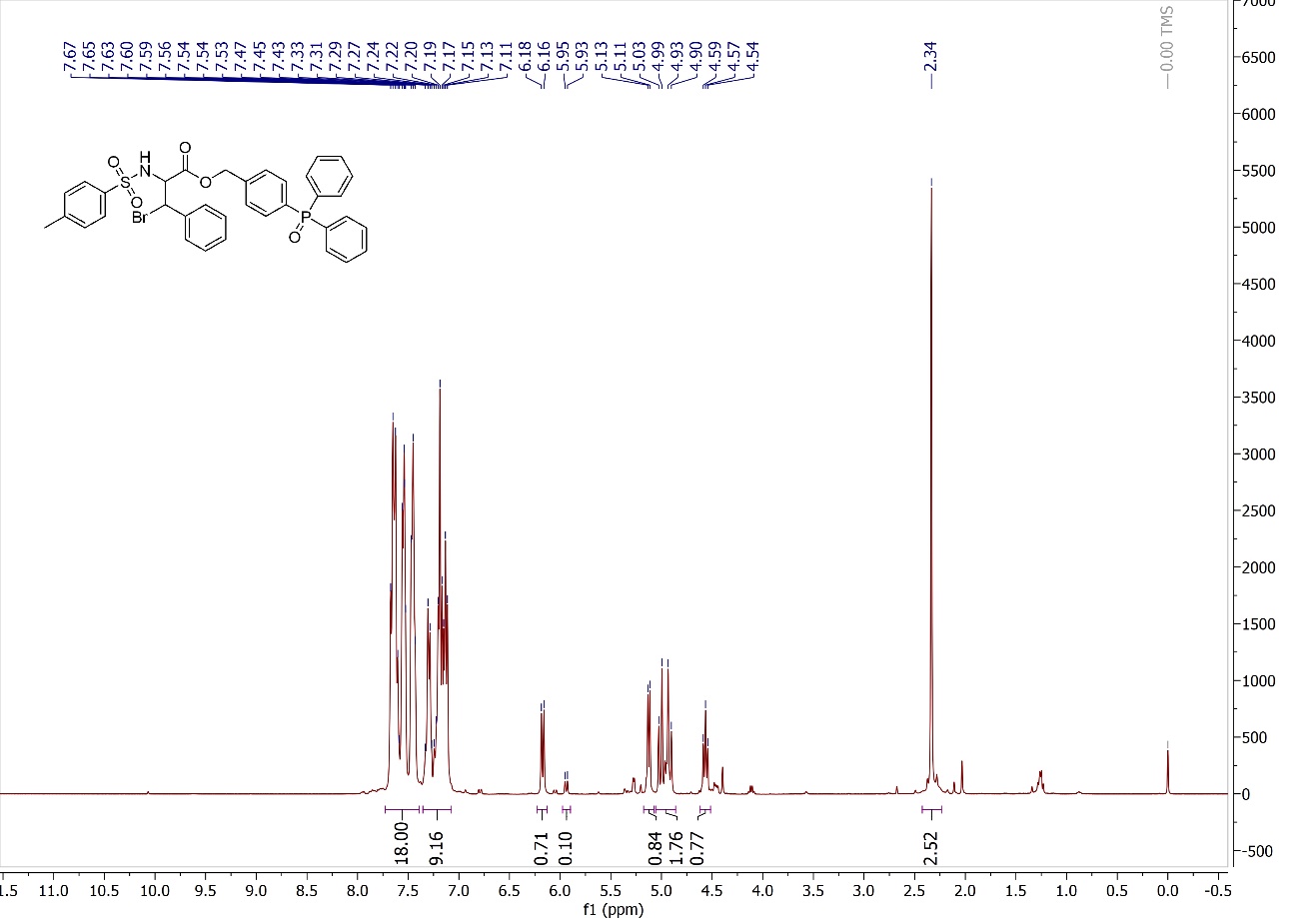


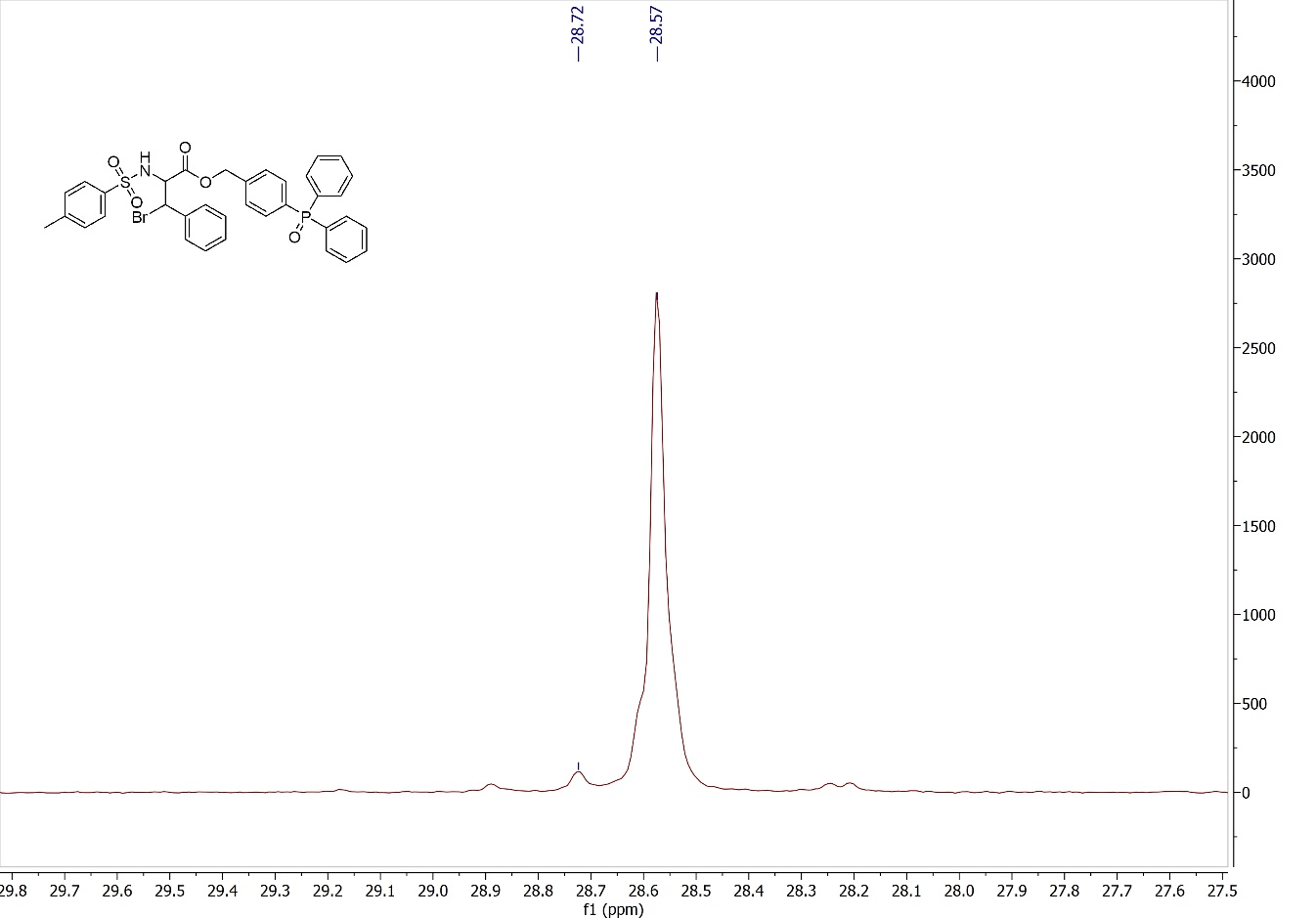


**2a**


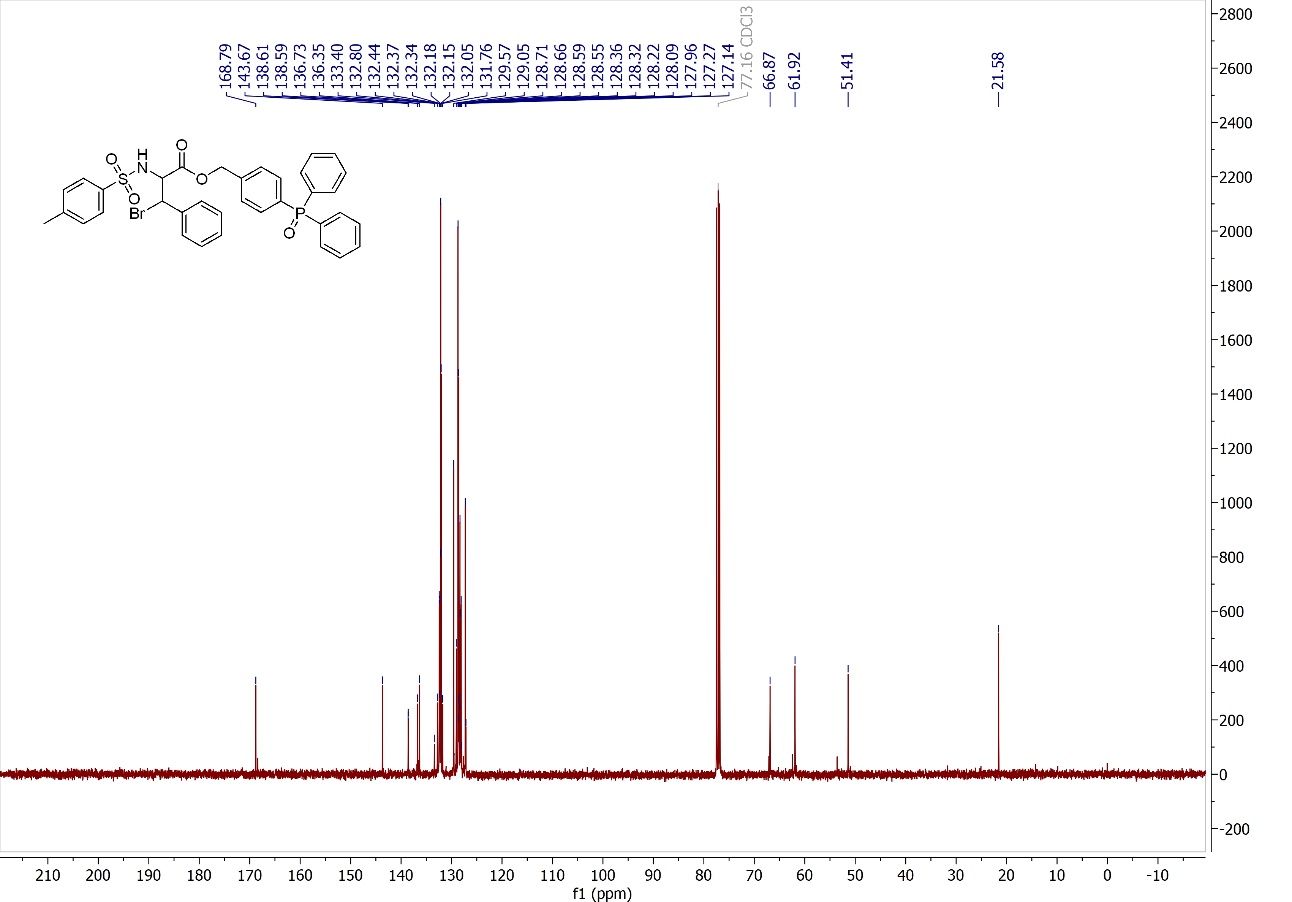


**2a**


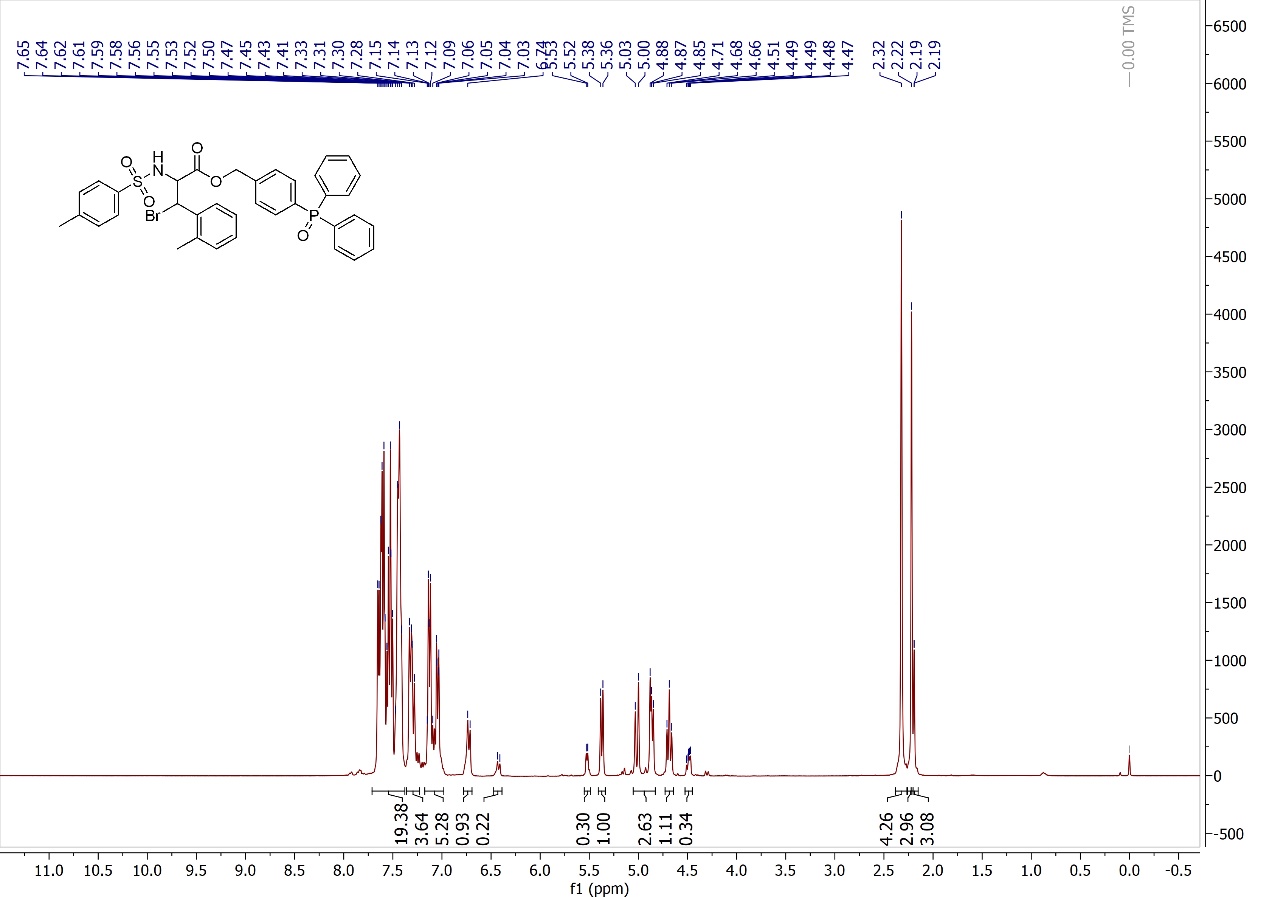

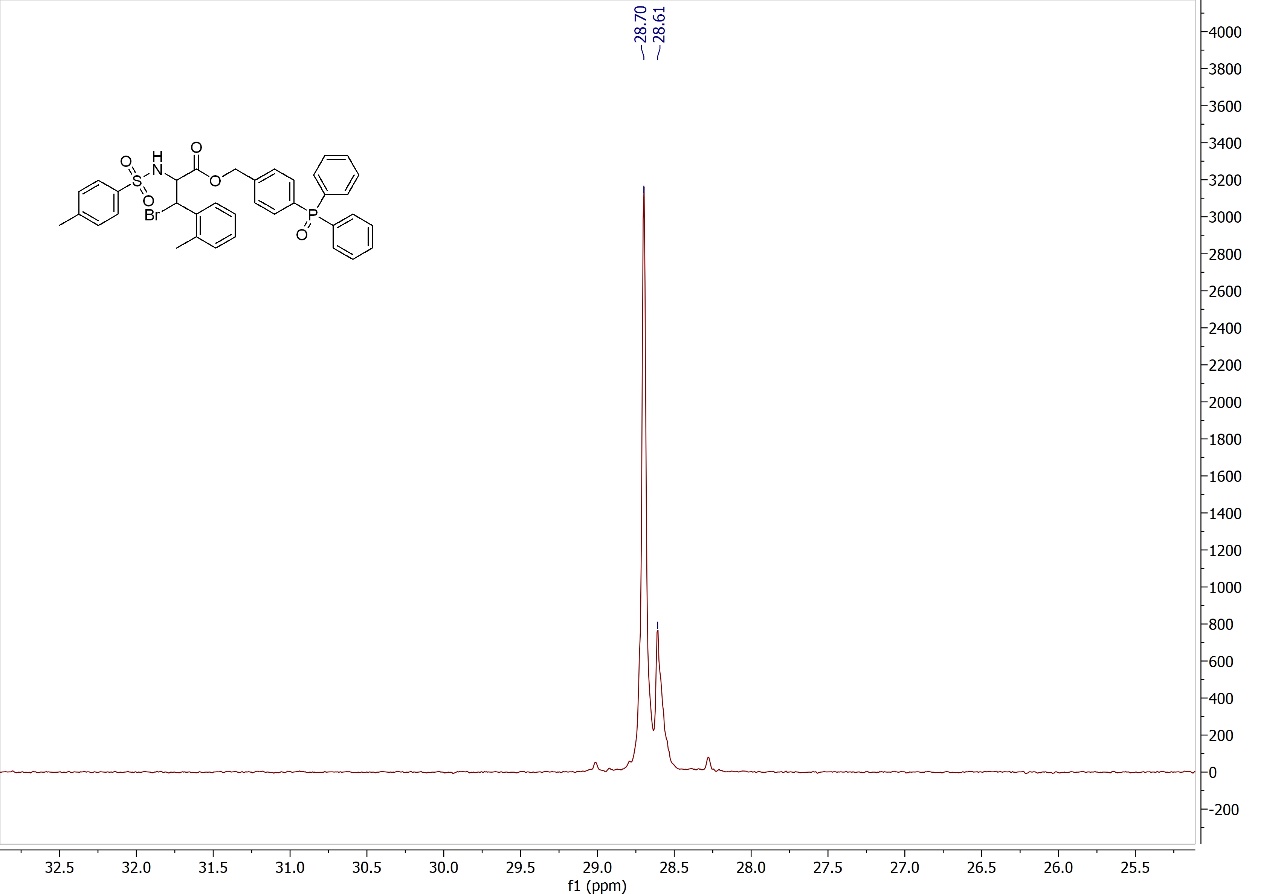


**2b**

**2b**


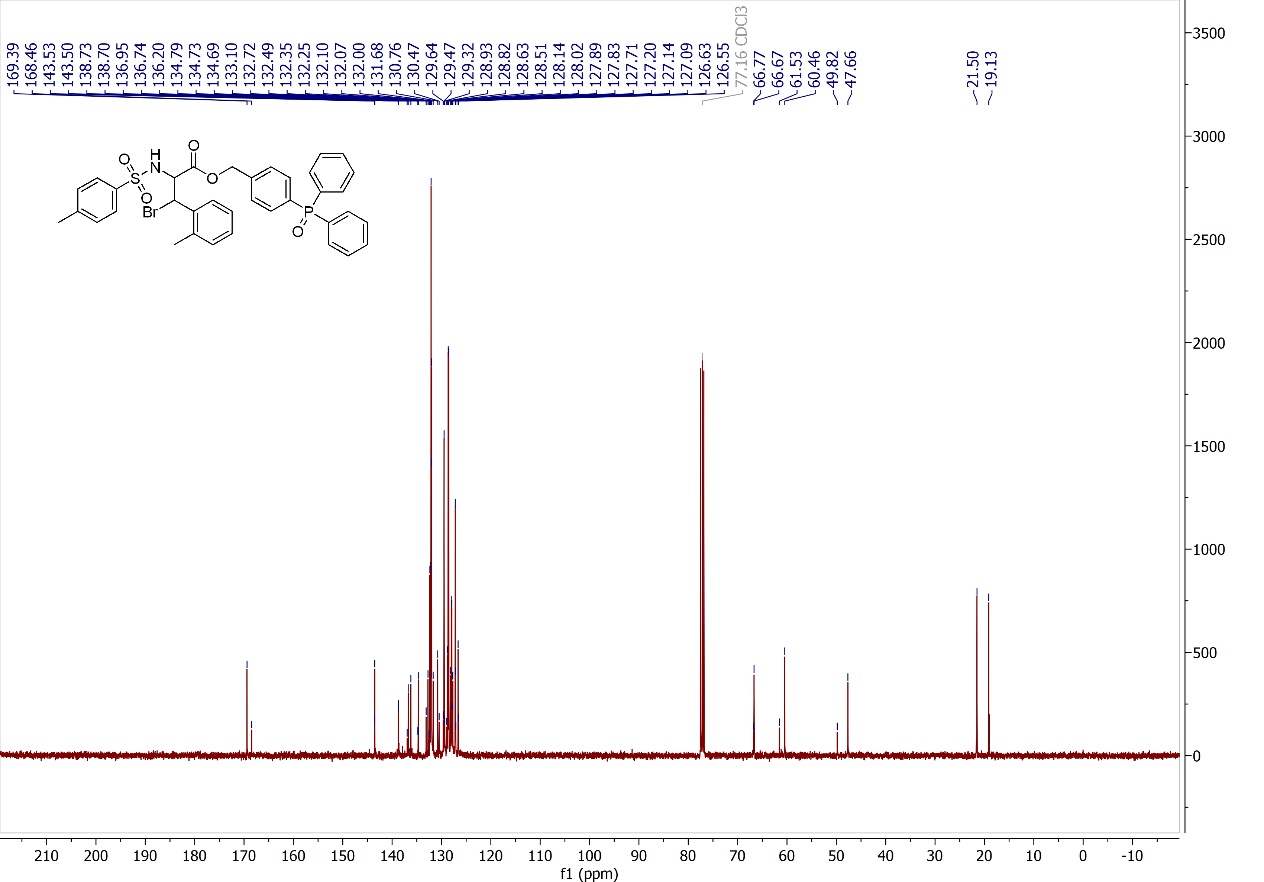


**2b**


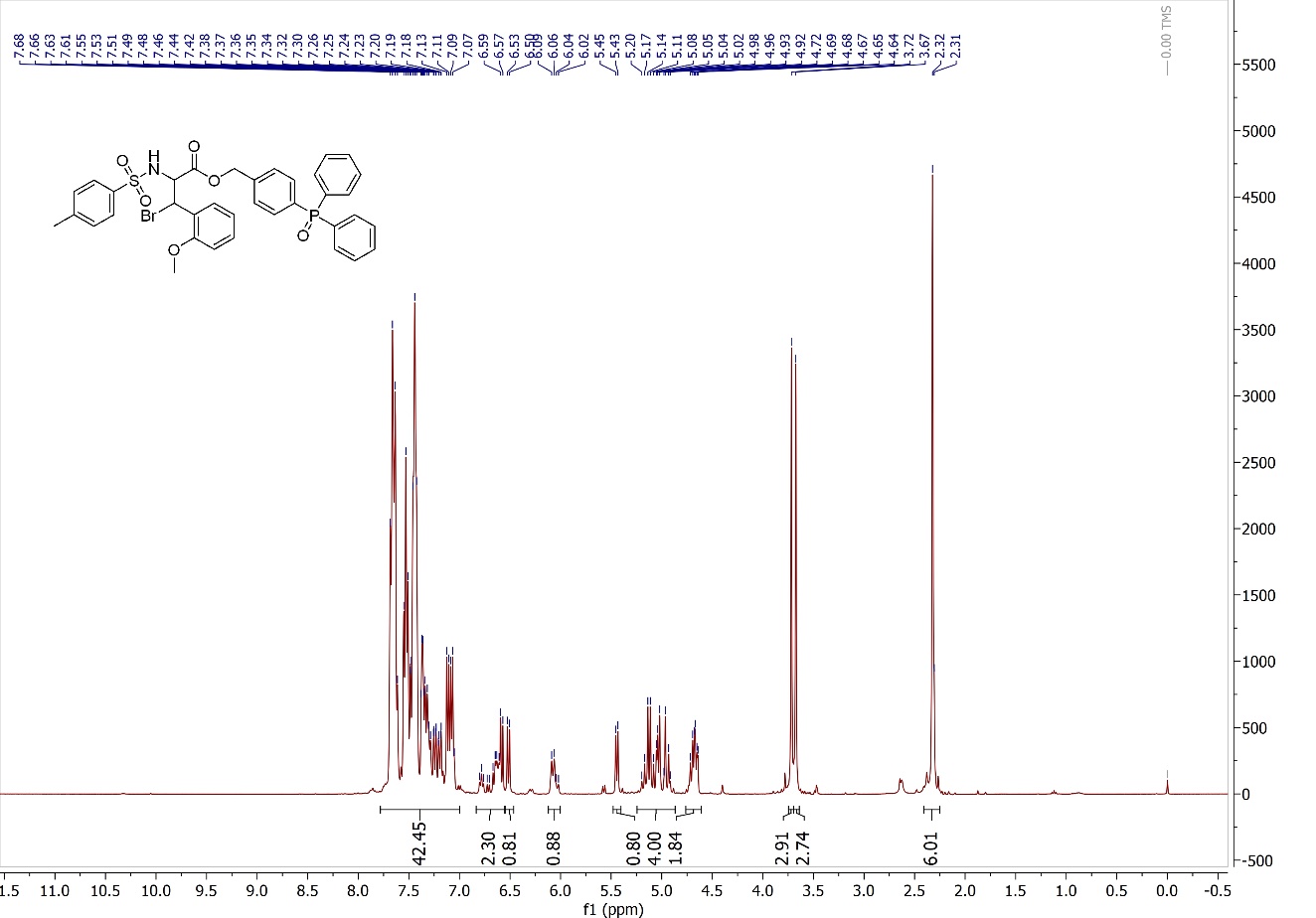

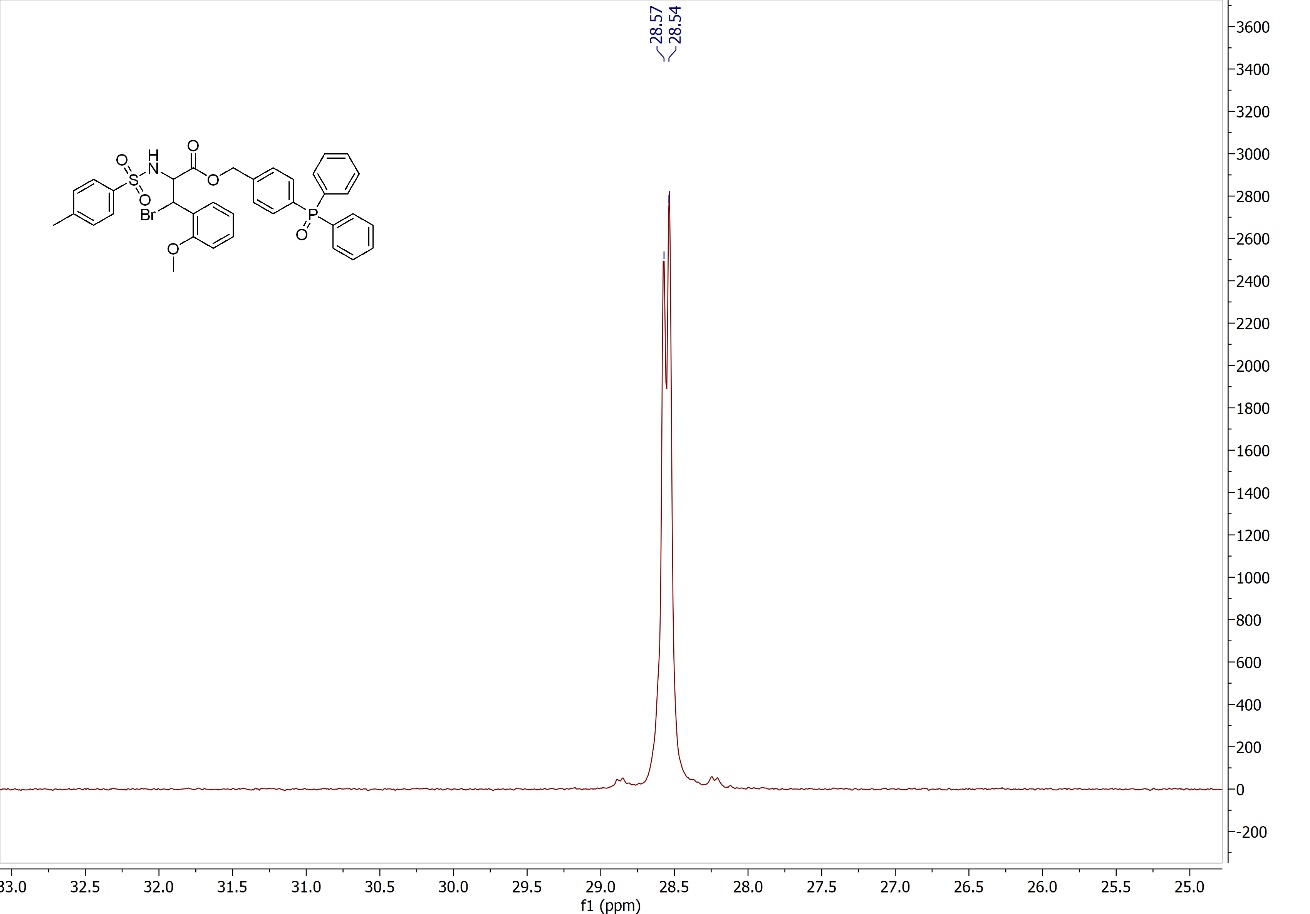


**2d**

**2d**


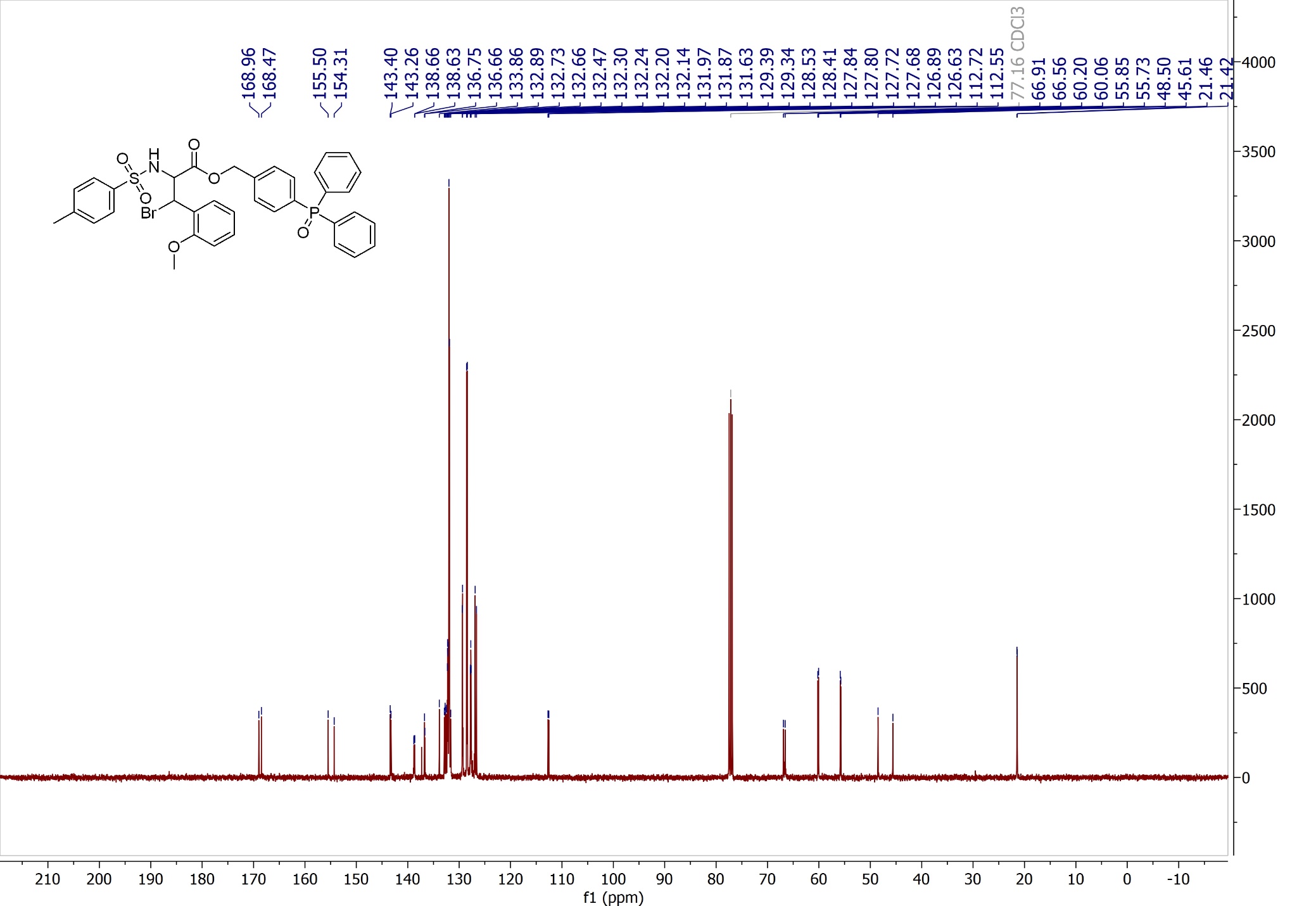


**2d**


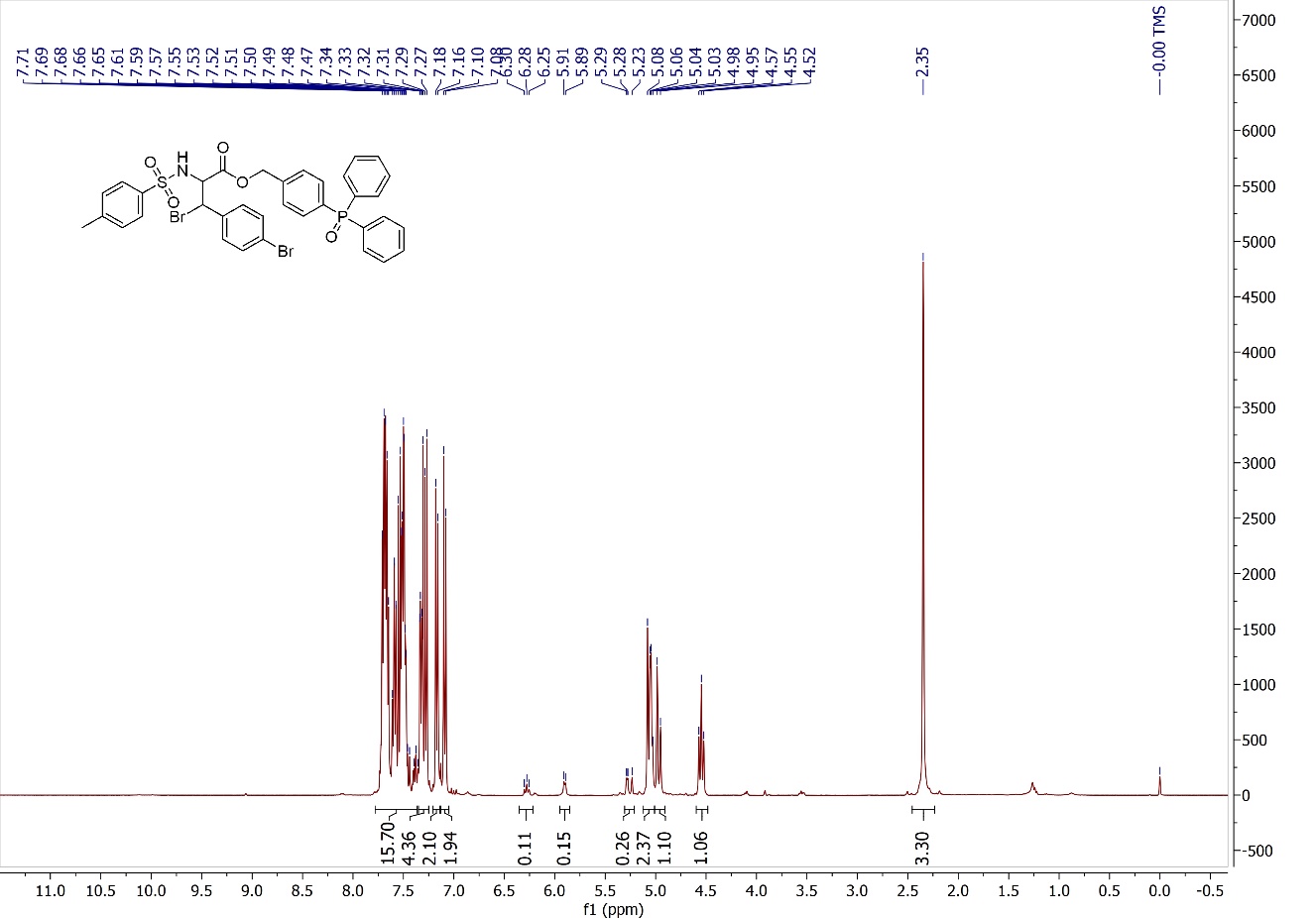

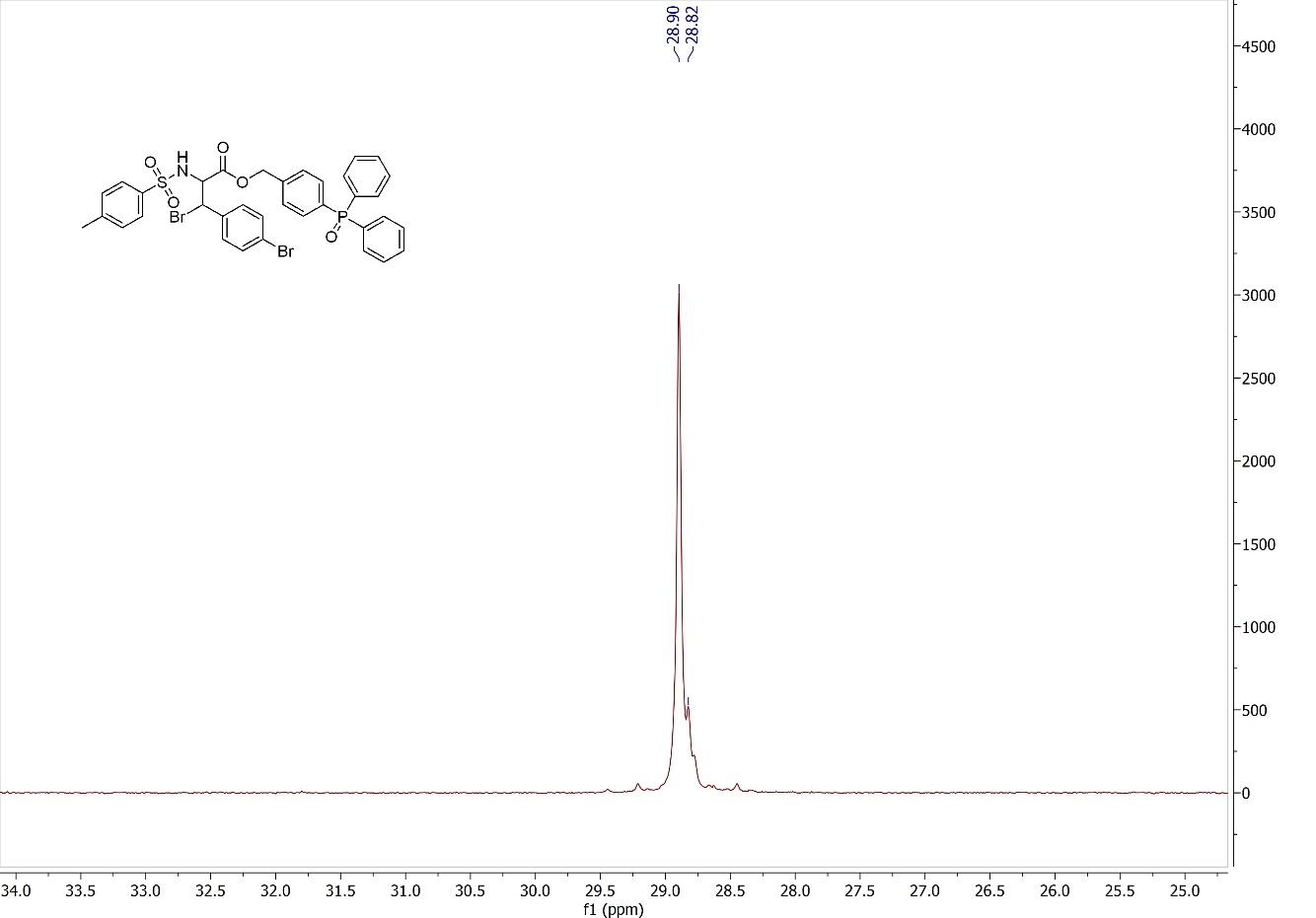


**2g**

**2g**


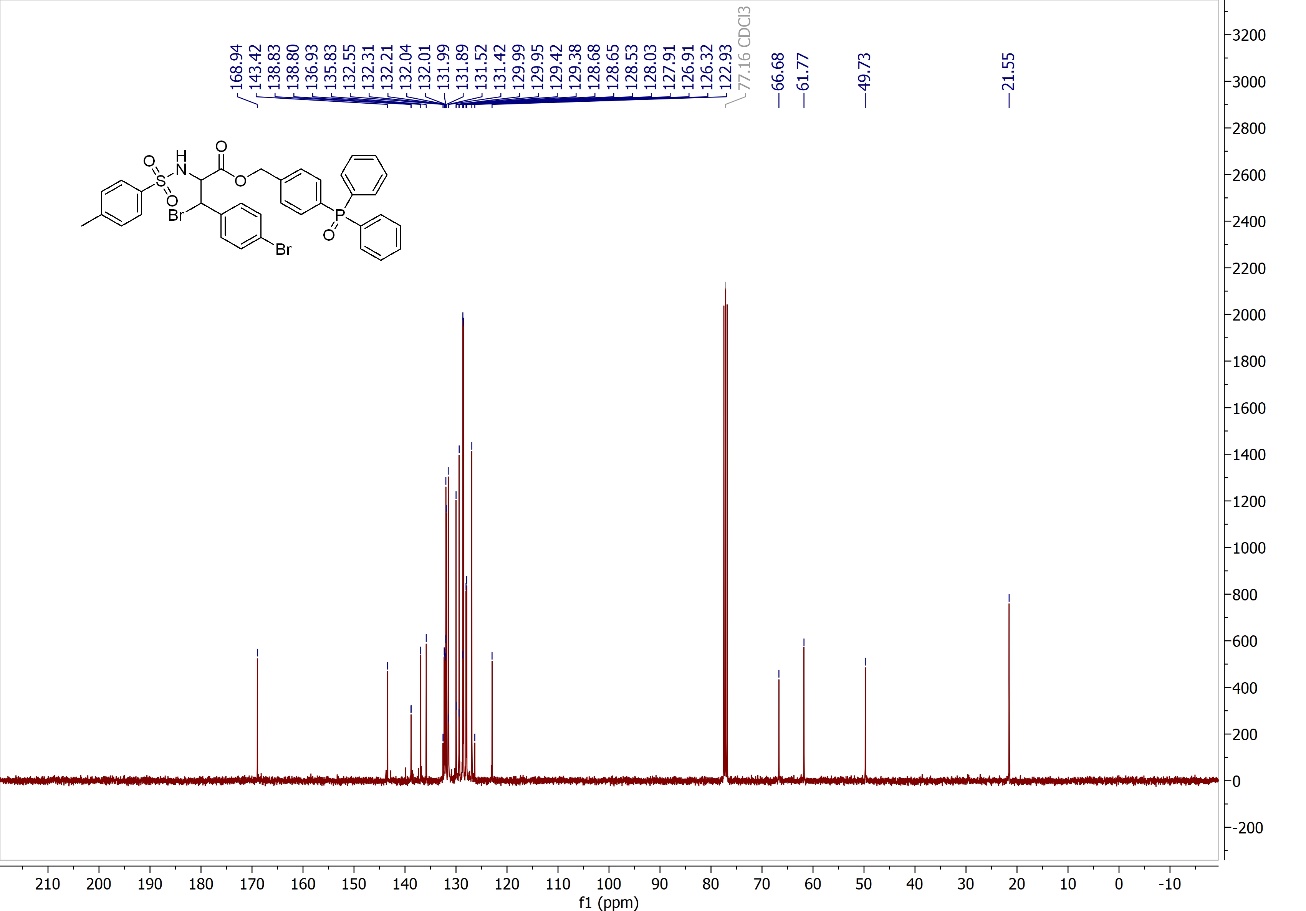


**2g**


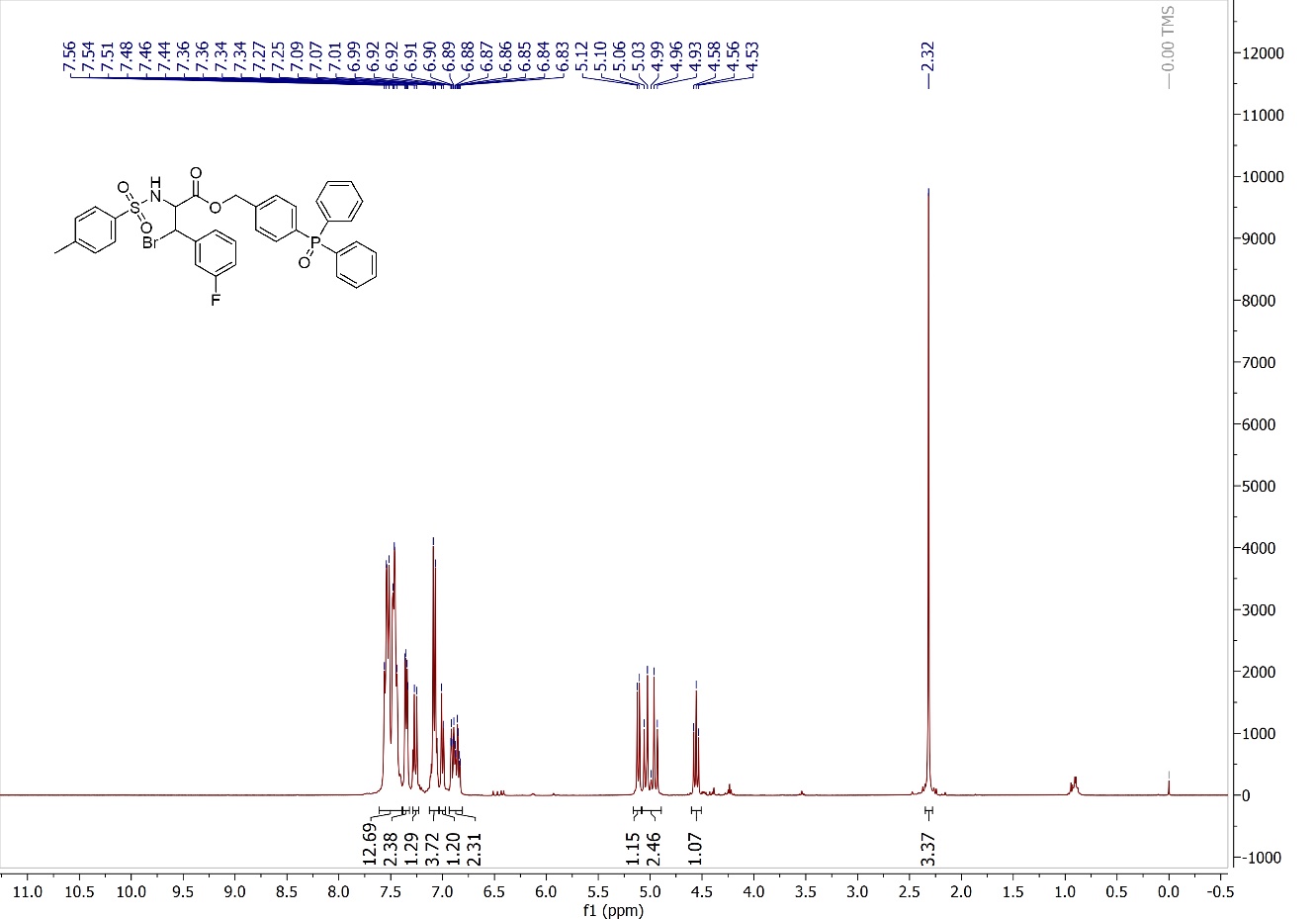


**2h**


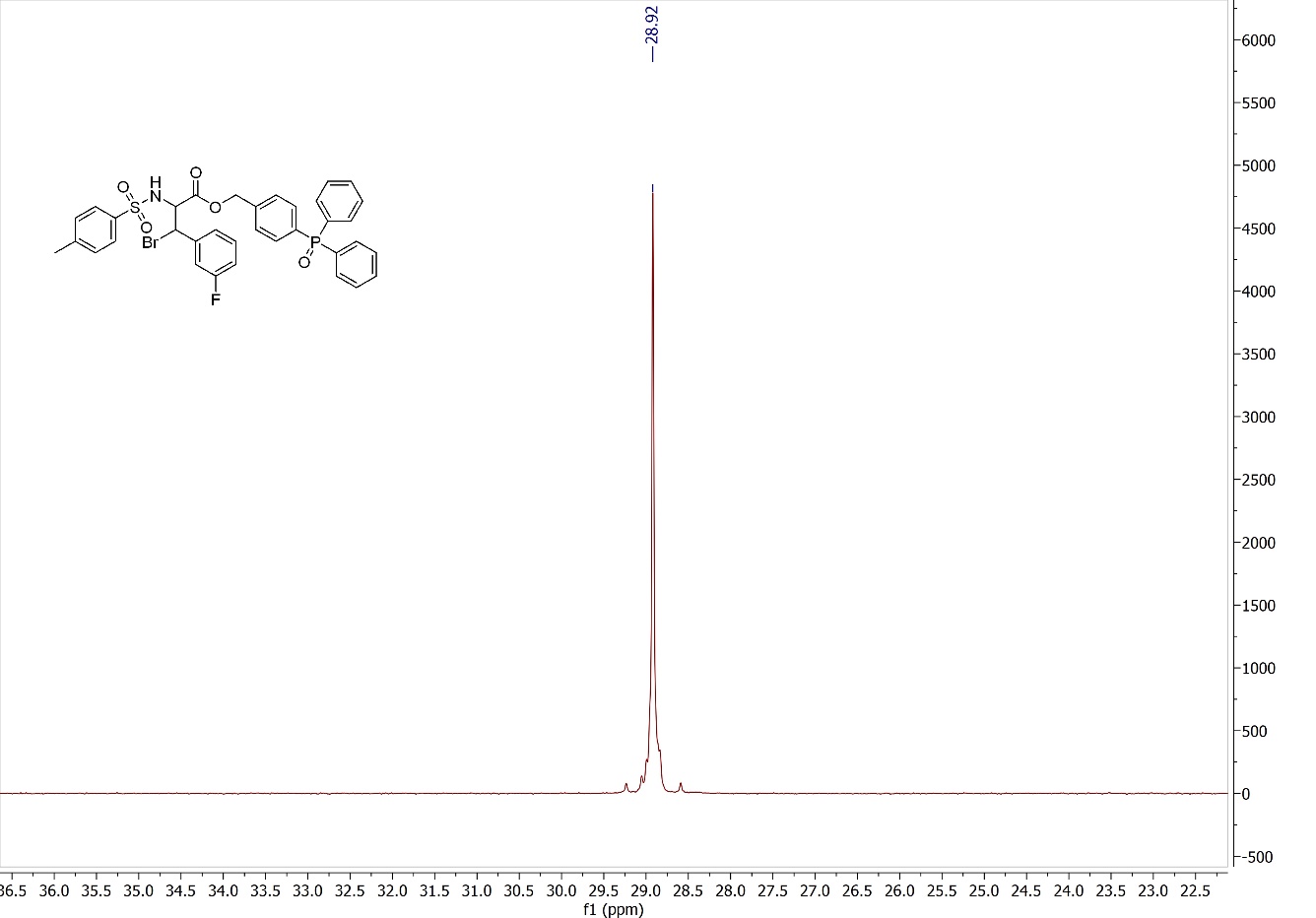


**2h**


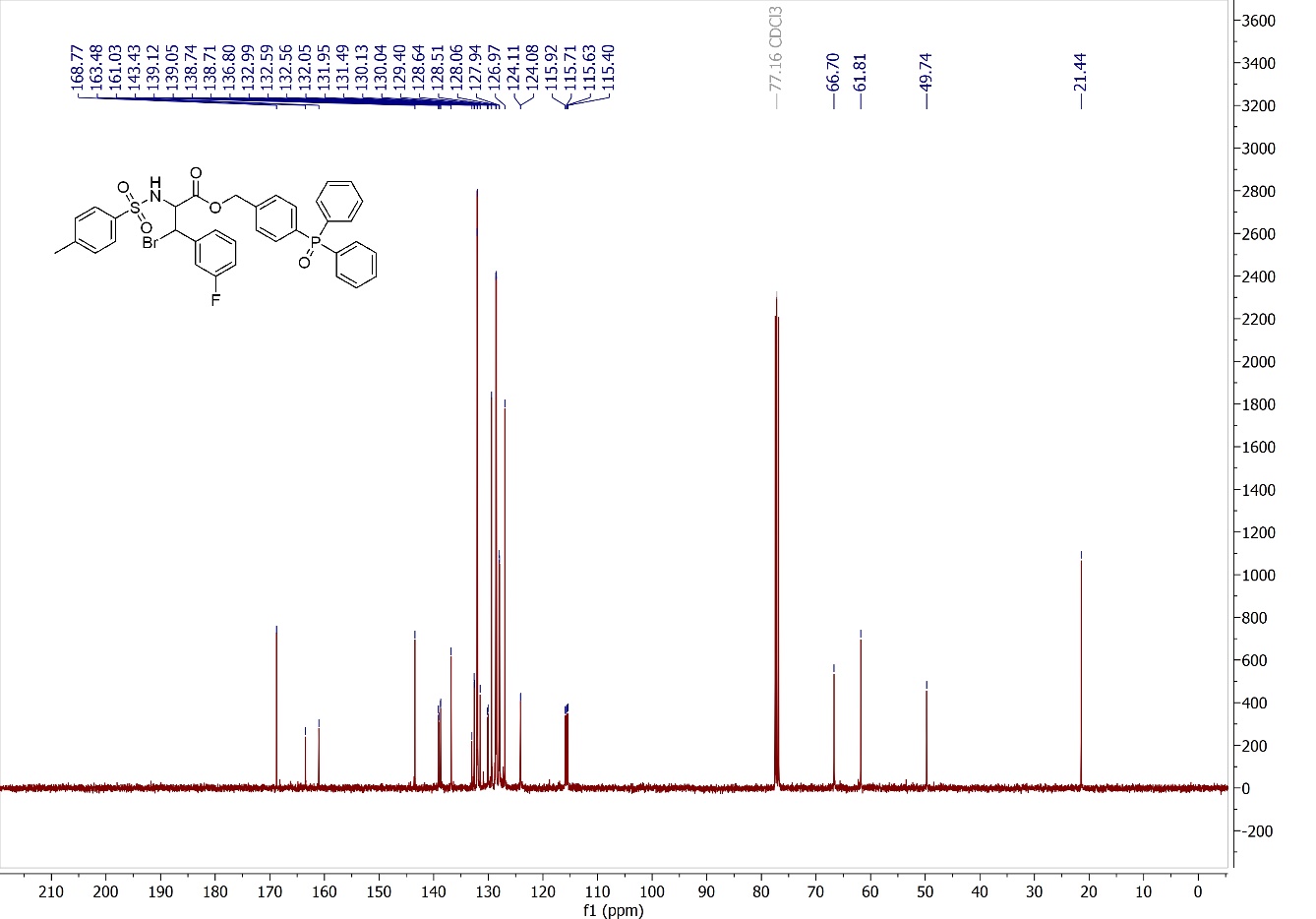


**2h**


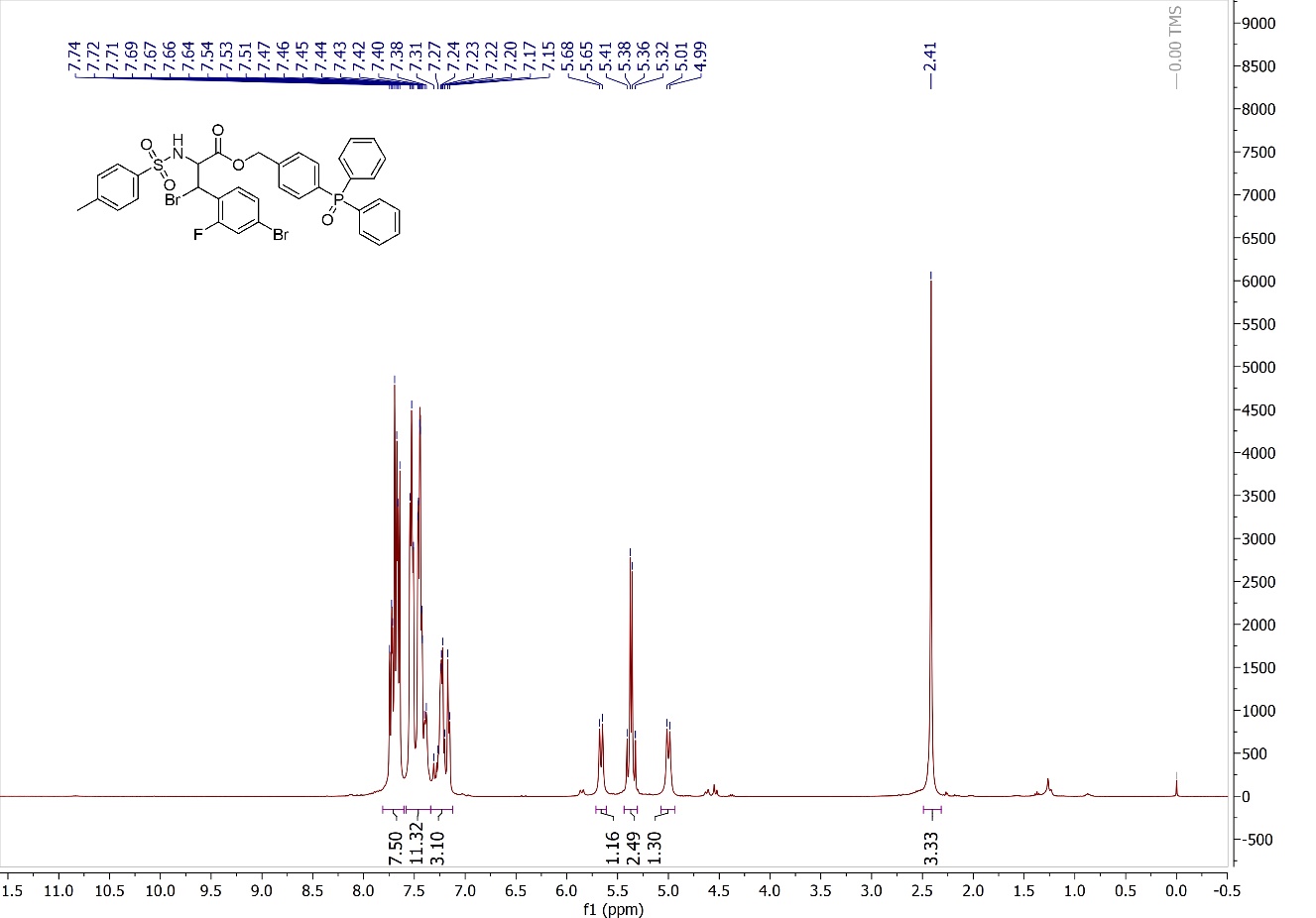


**2i**


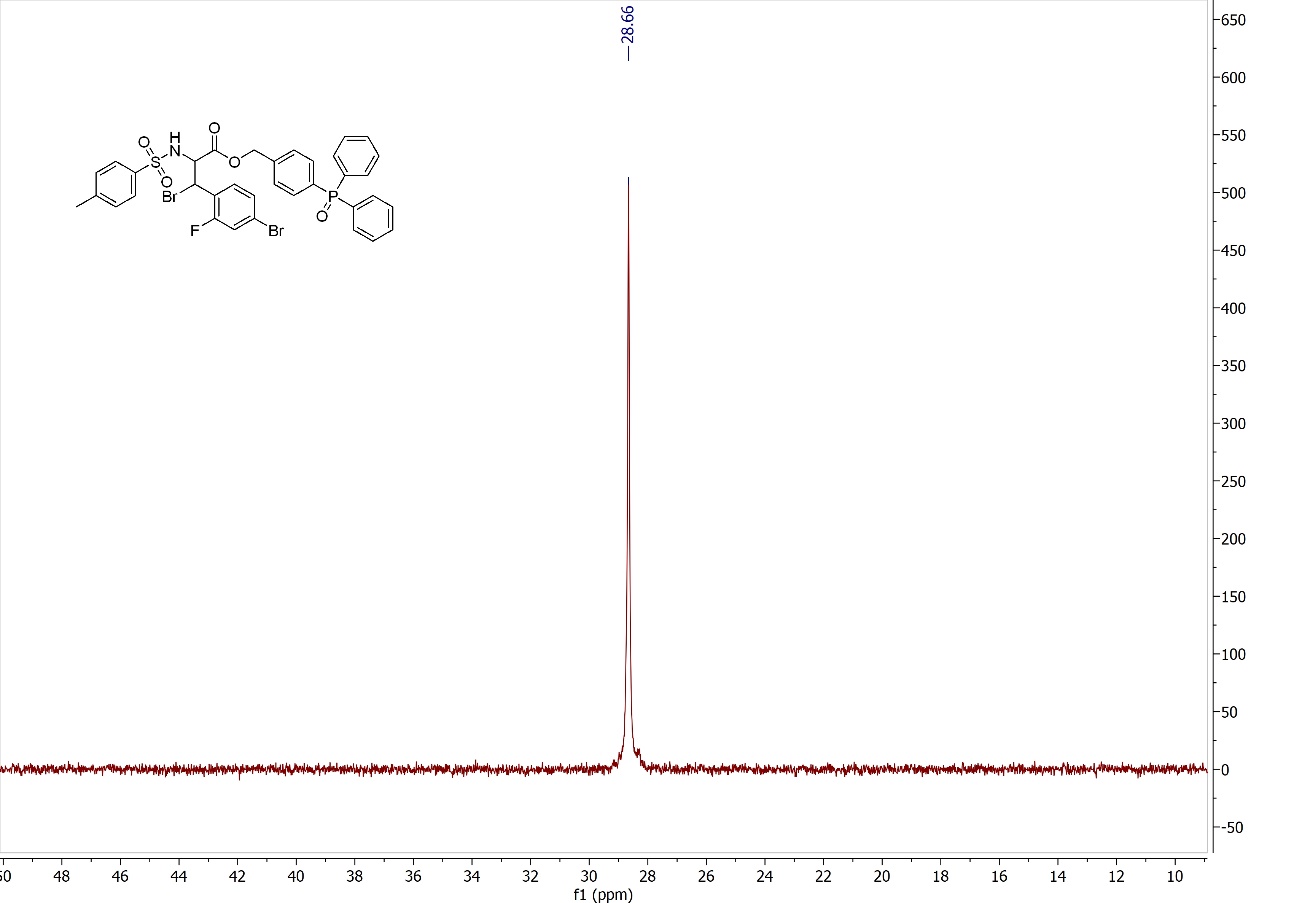


**2i**


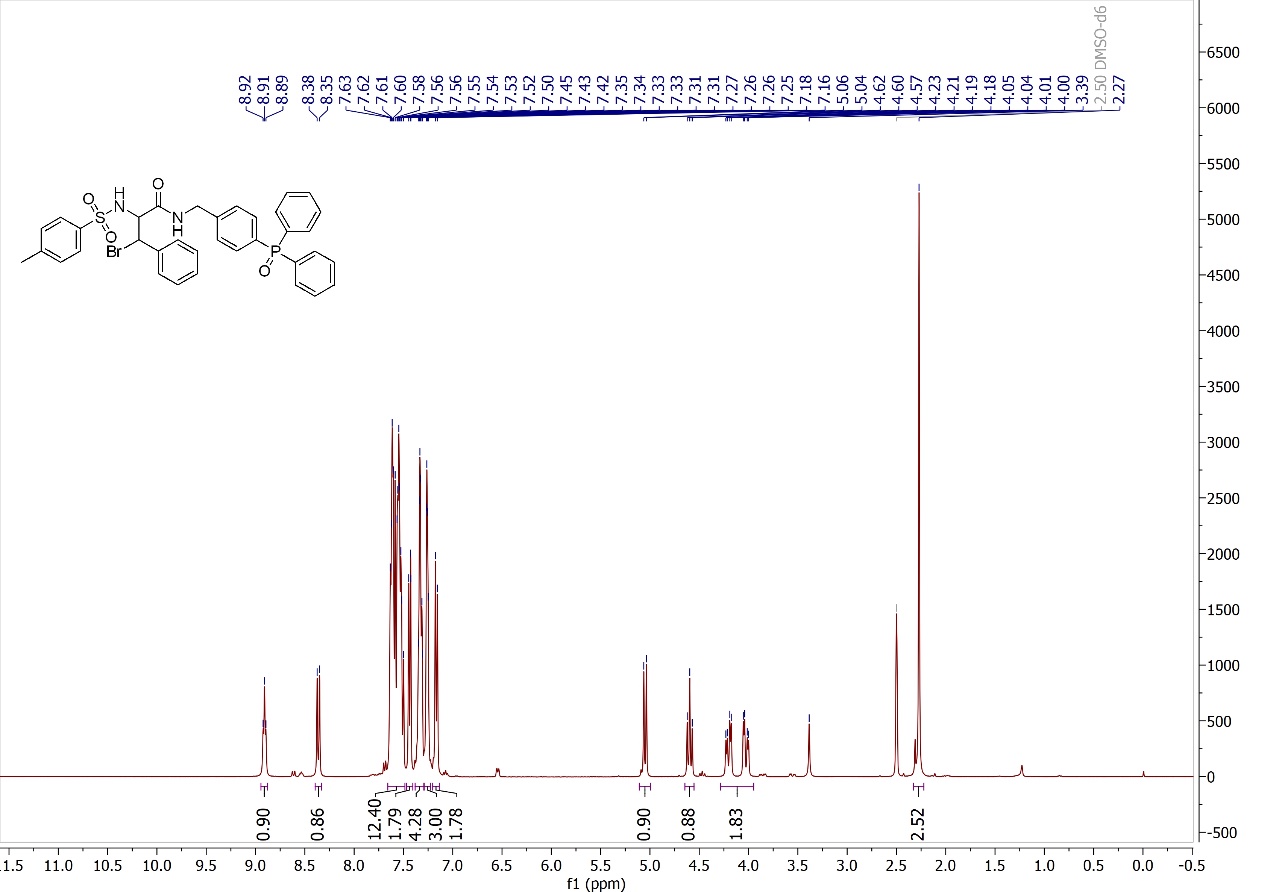


**4a**


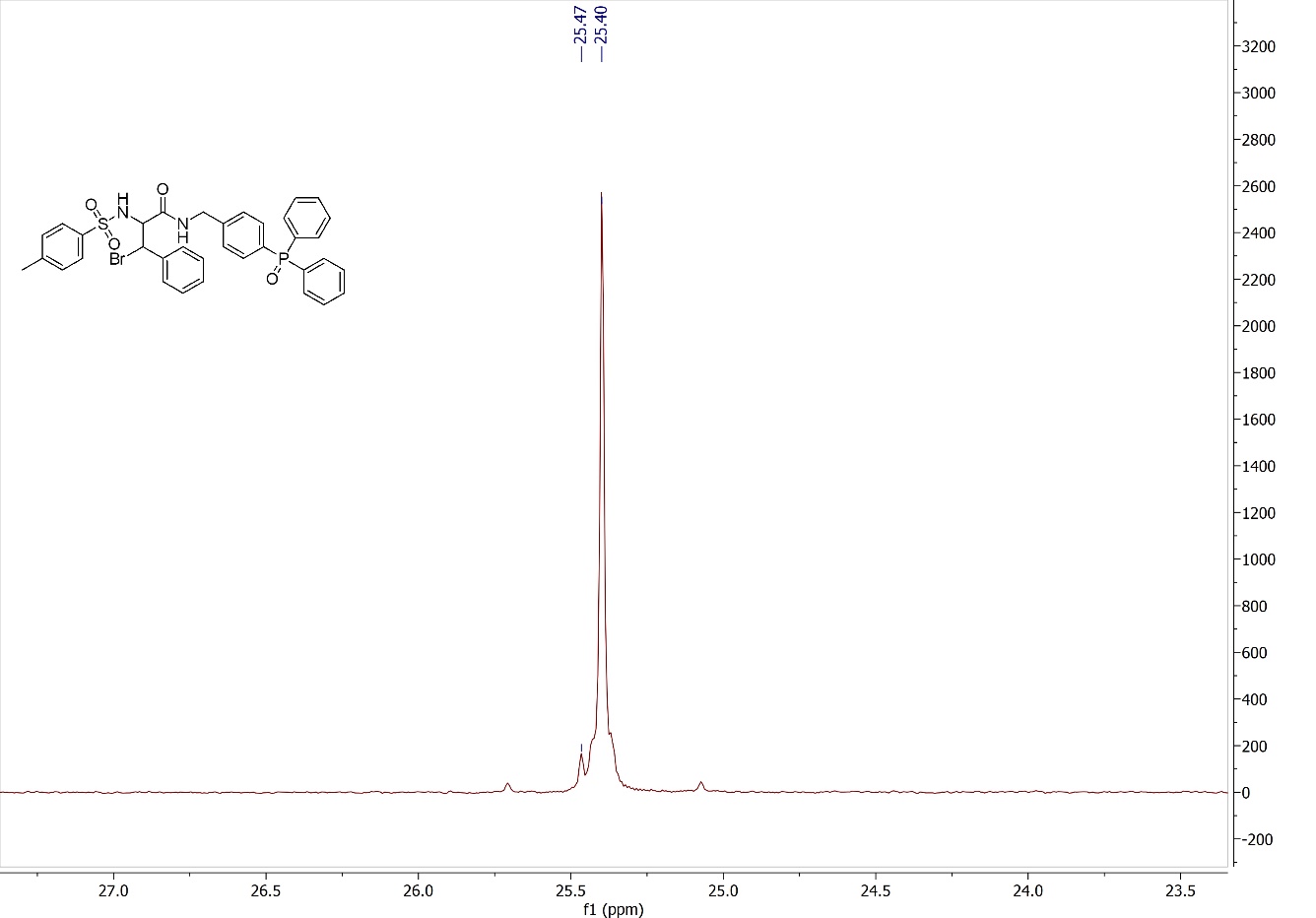


**4a**


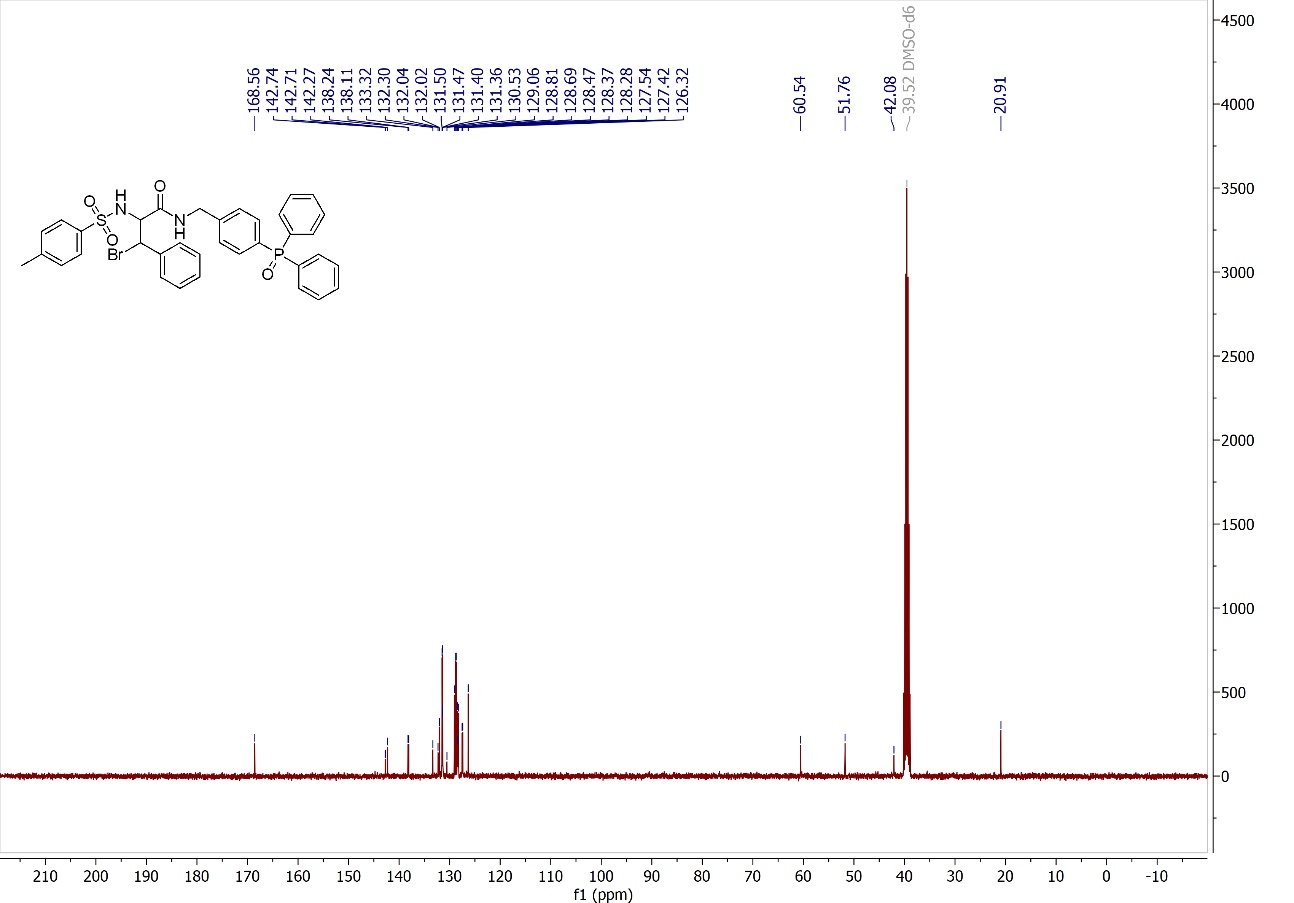


**4a**


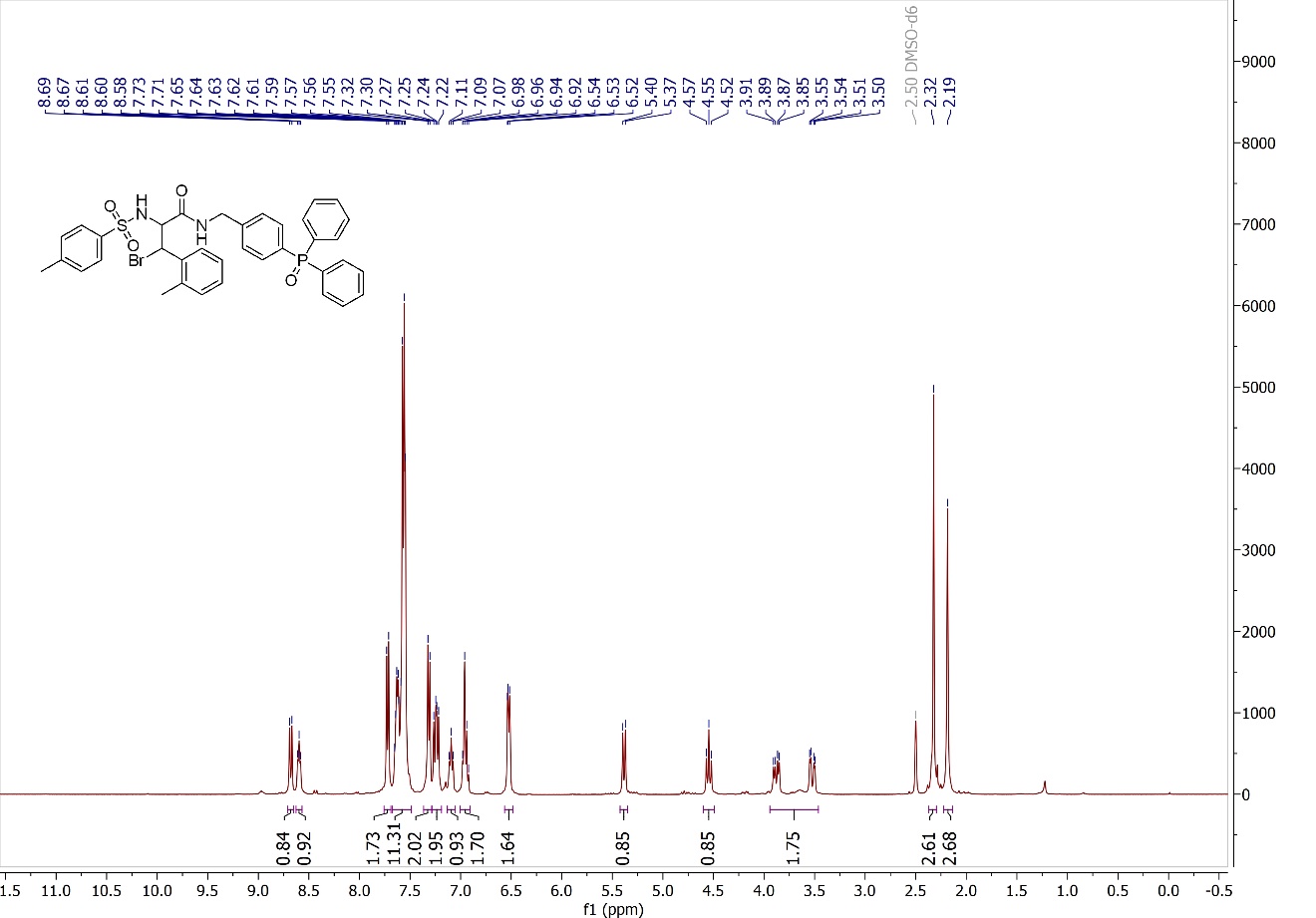


**4b**


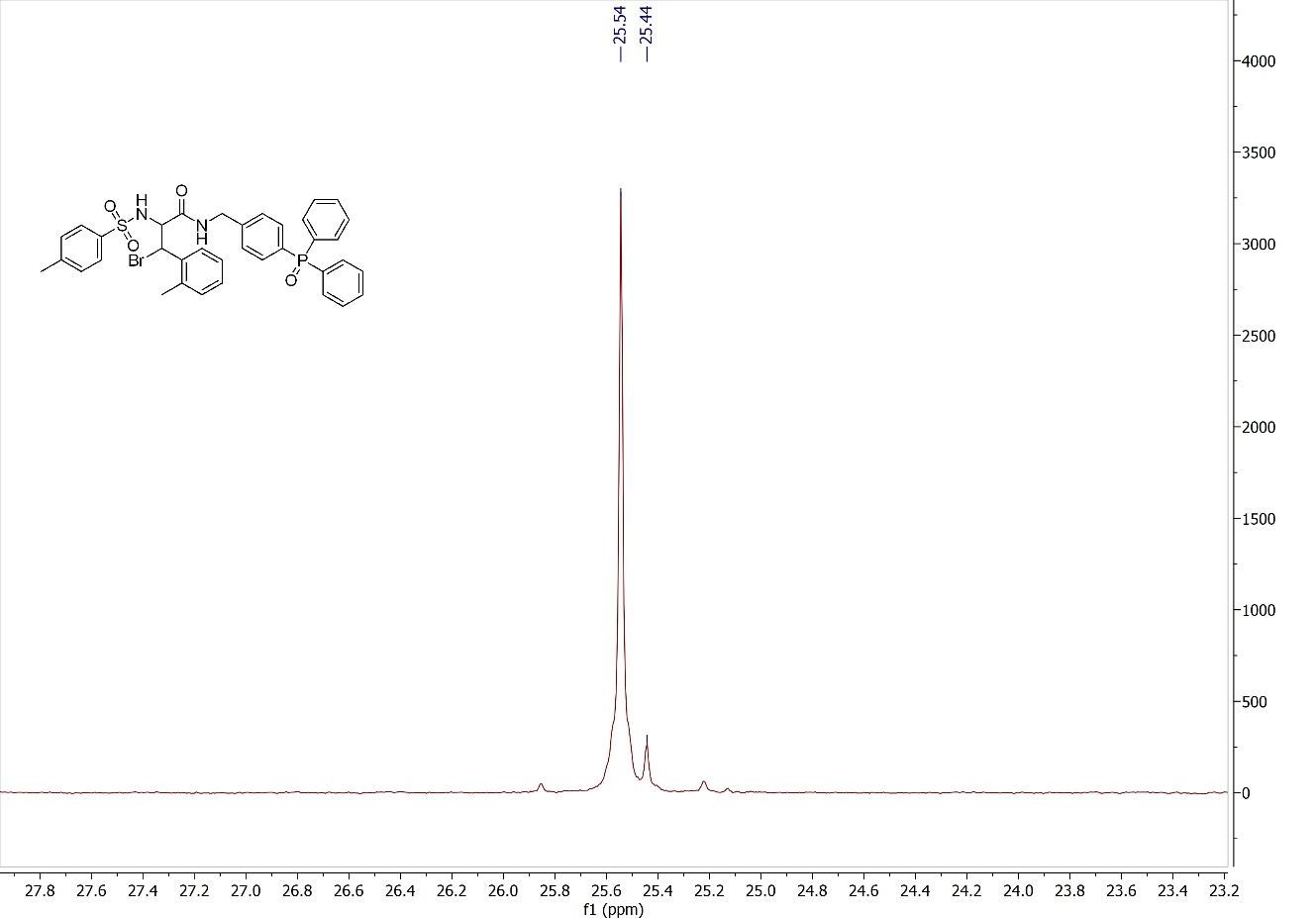


**4b**


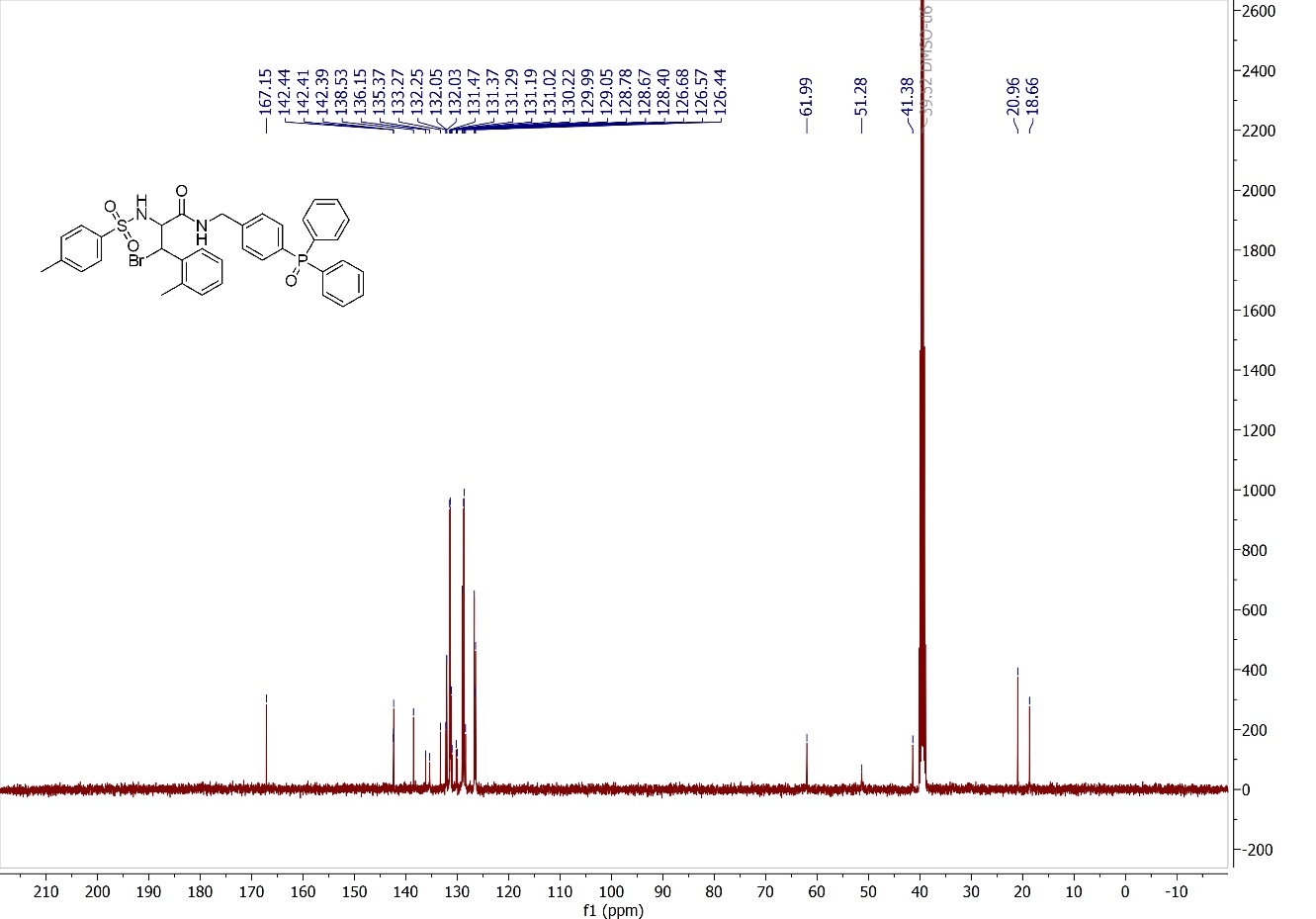


**4b**


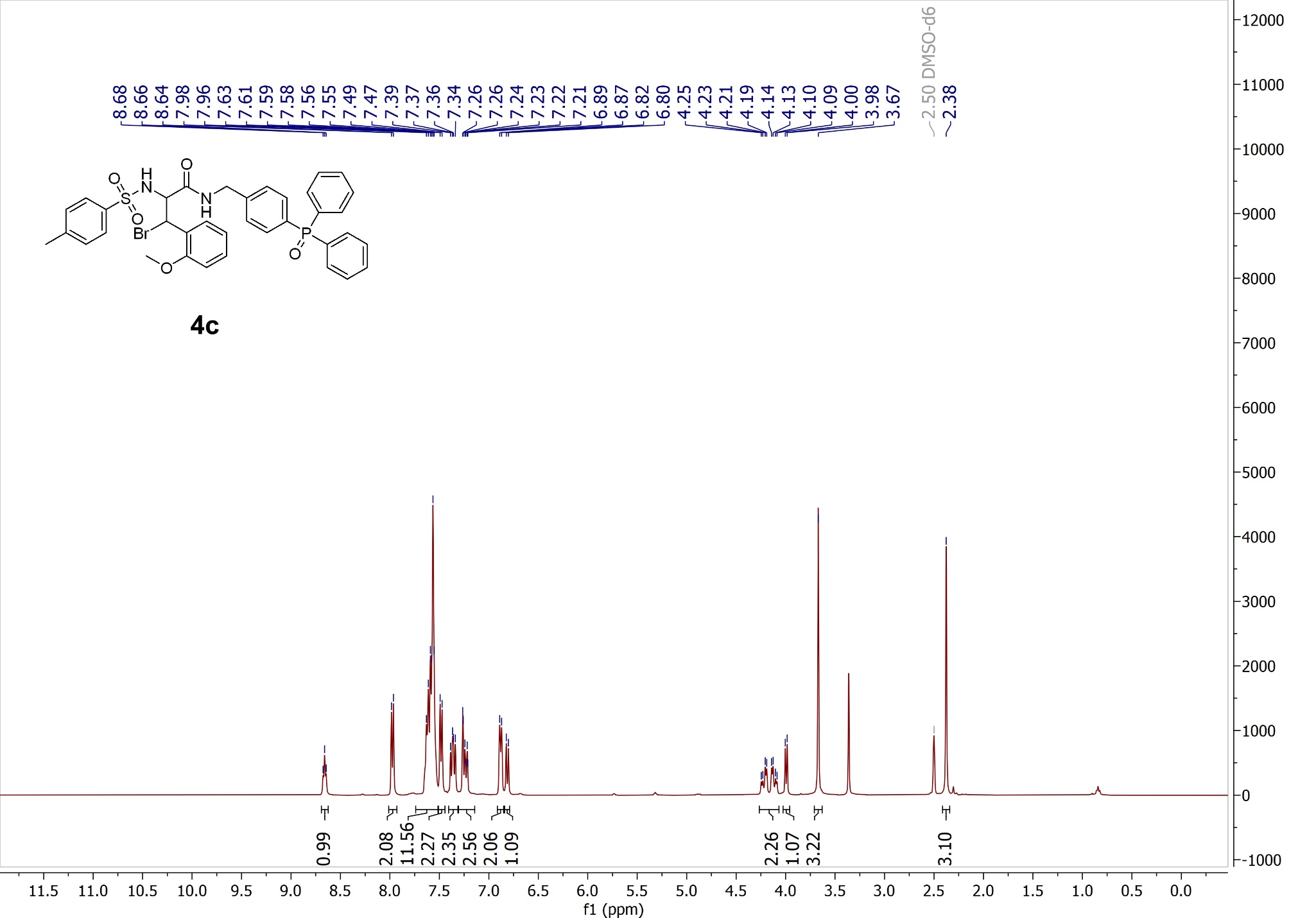


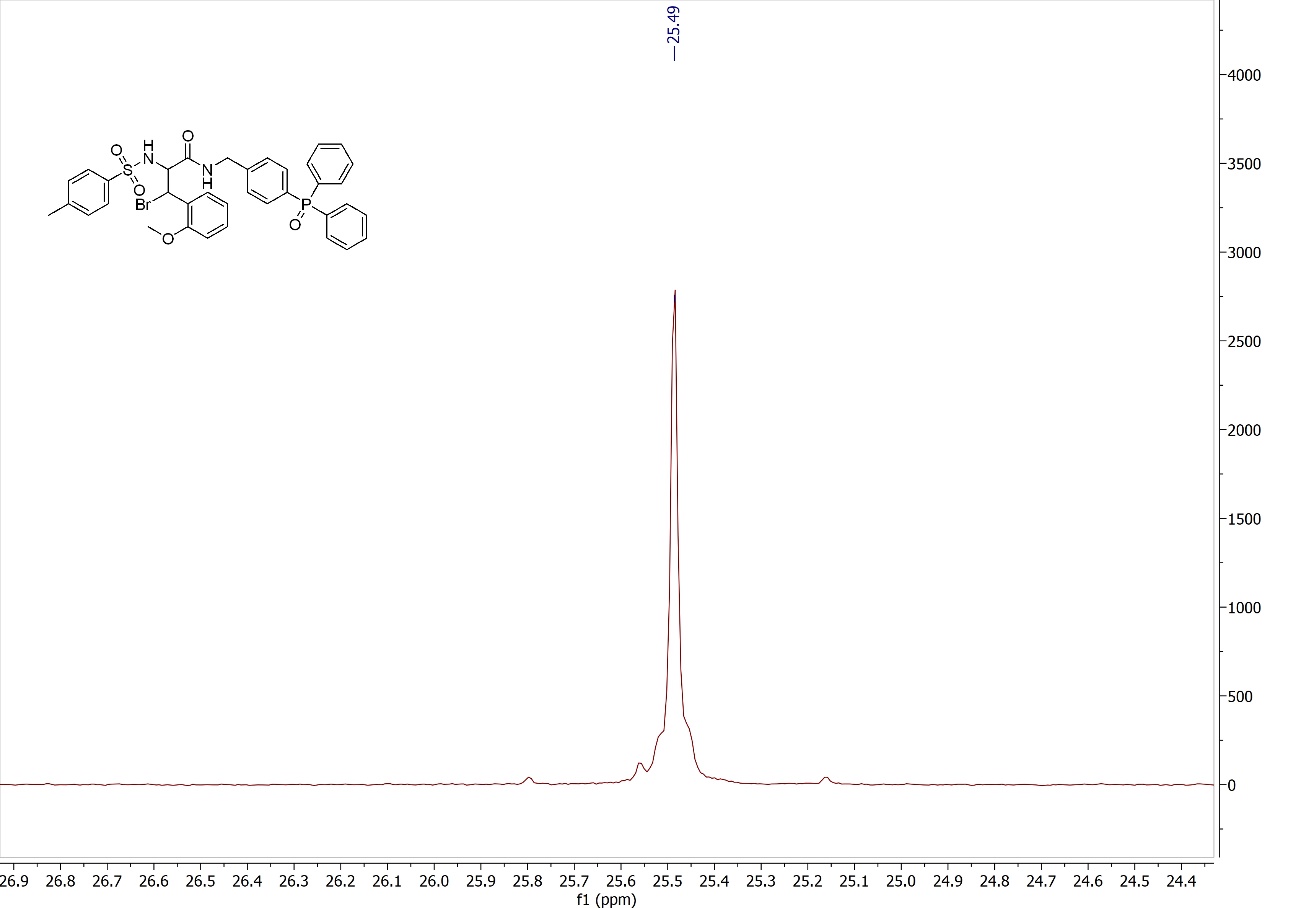


**4c**


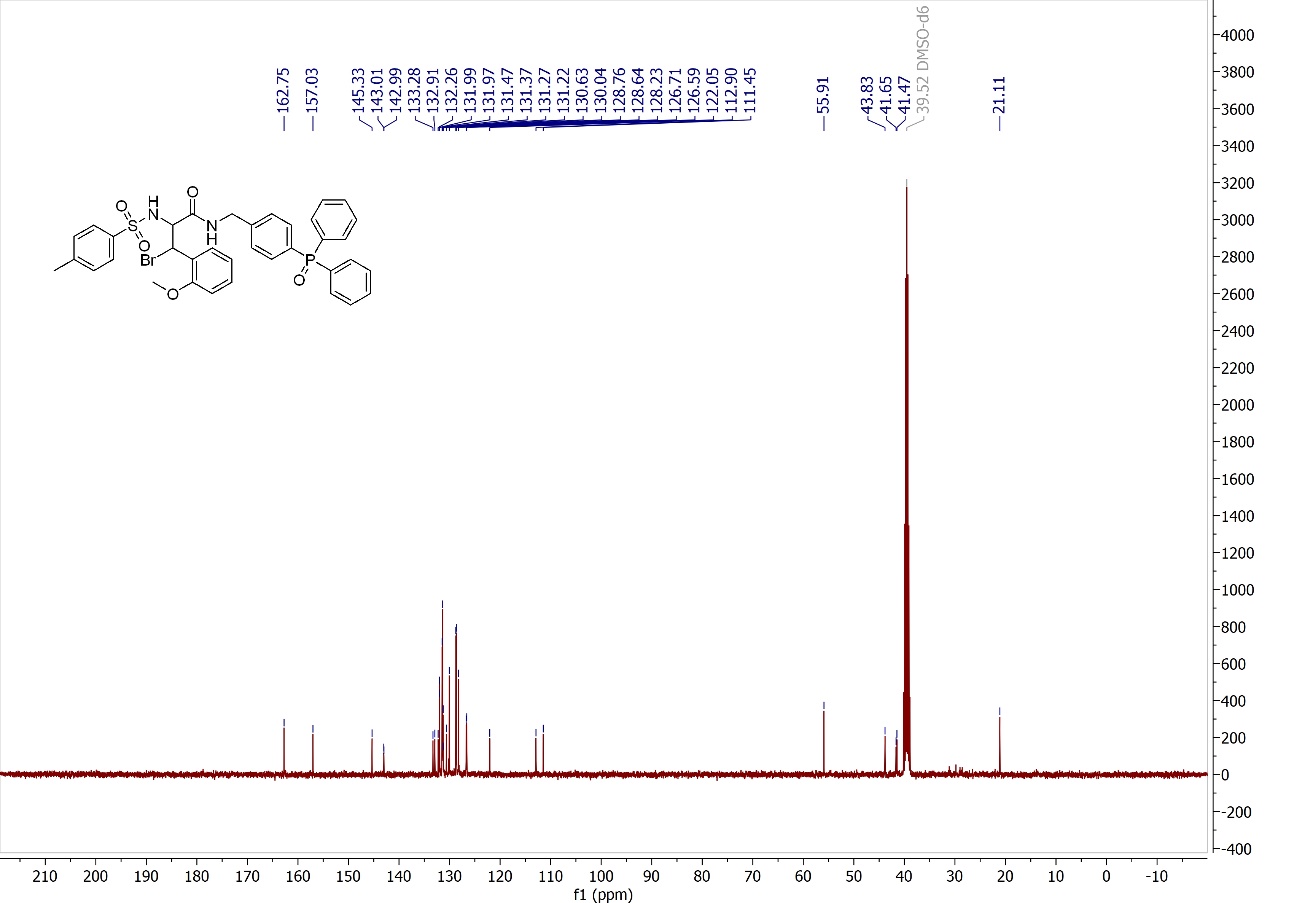


**4c**


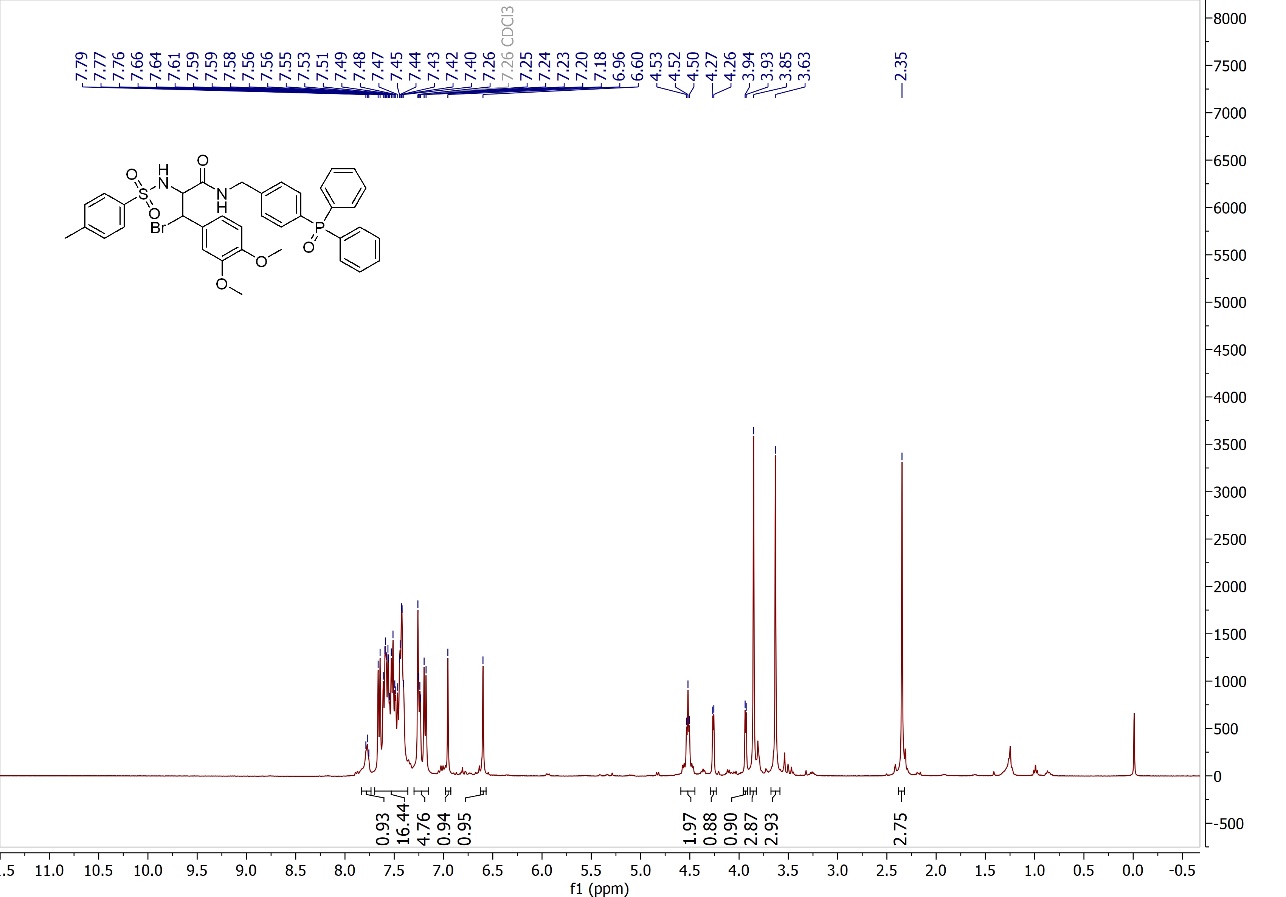


**4d**


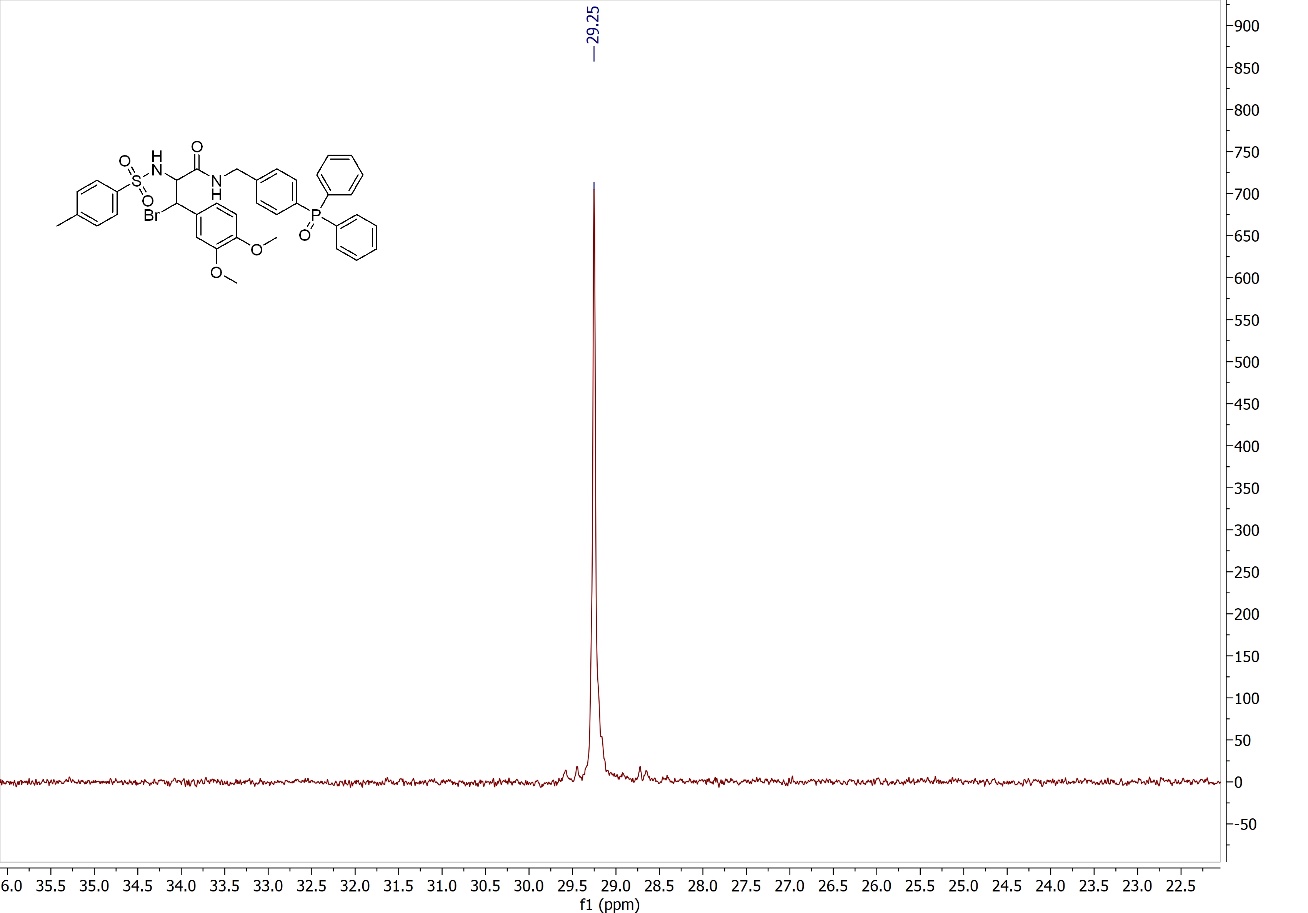


**4d**


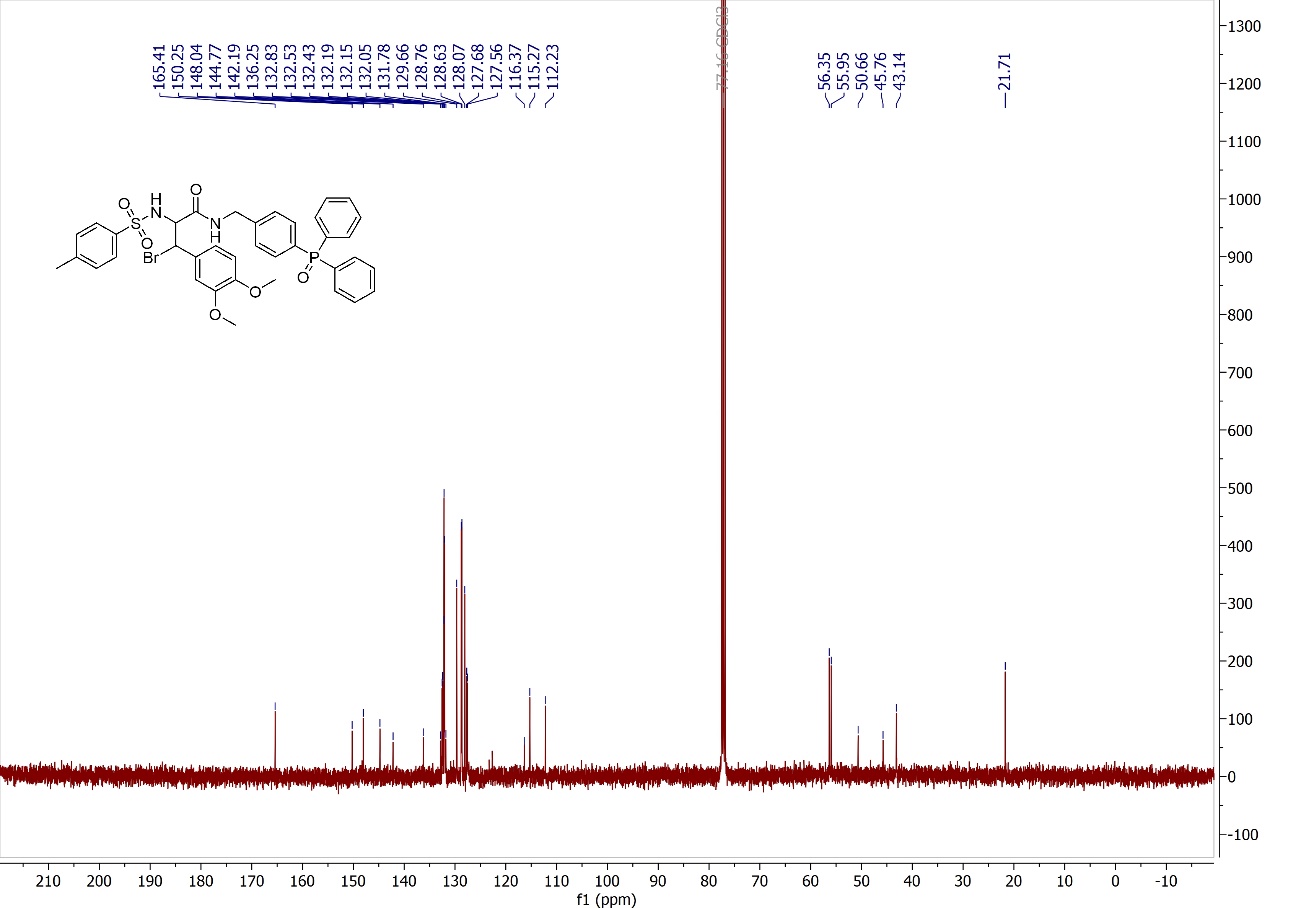


**4d**


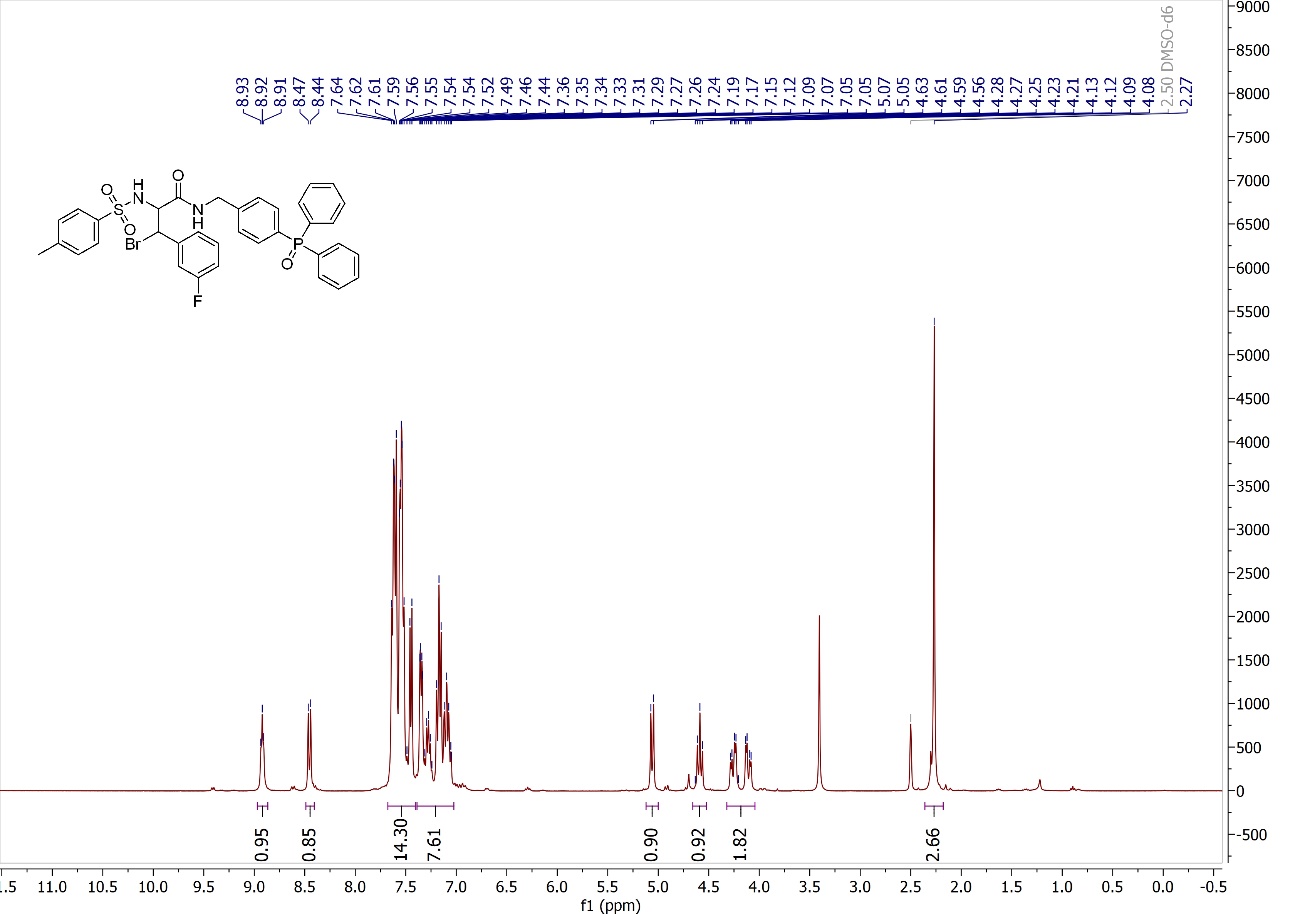

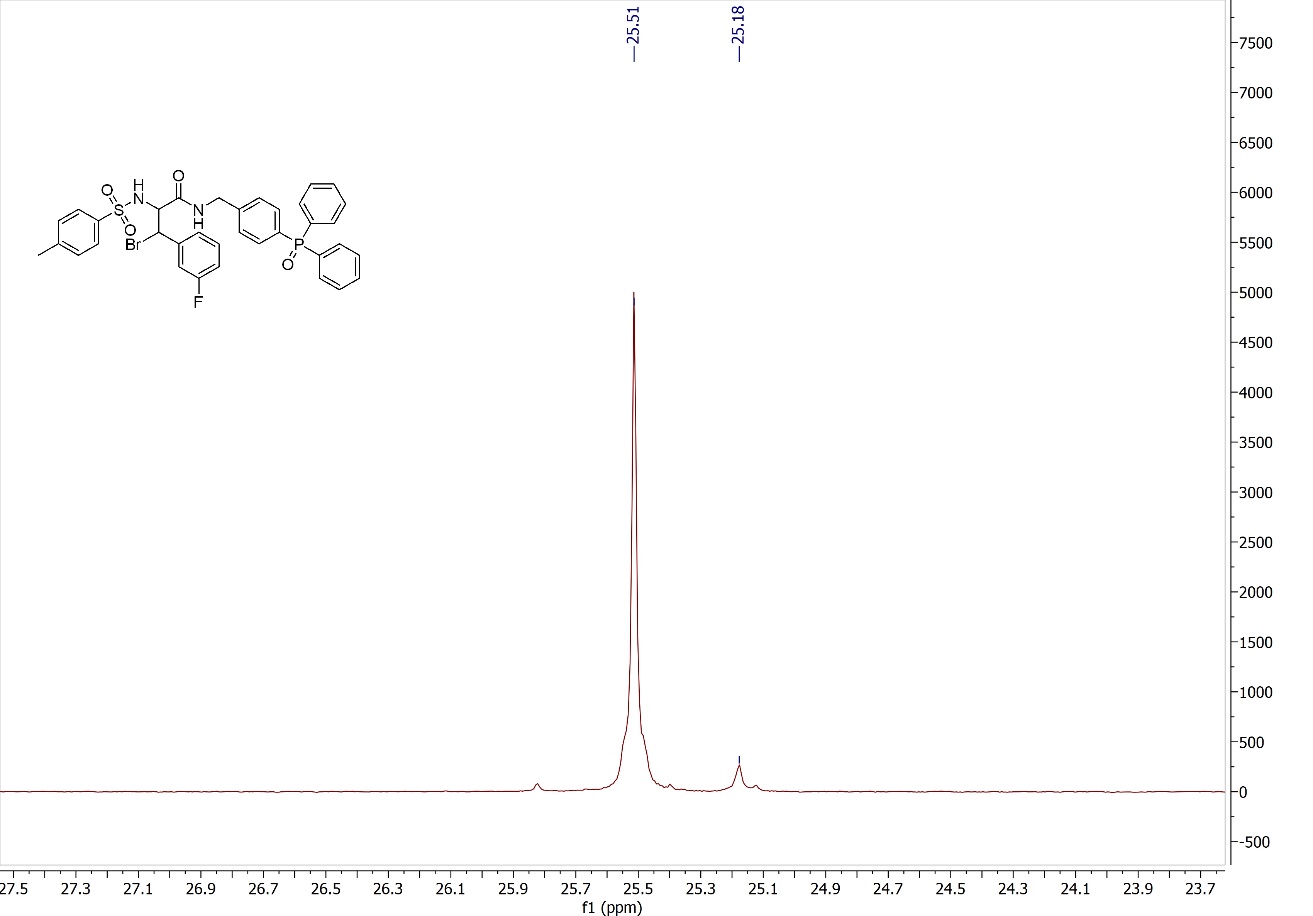


**4g**

**4g**


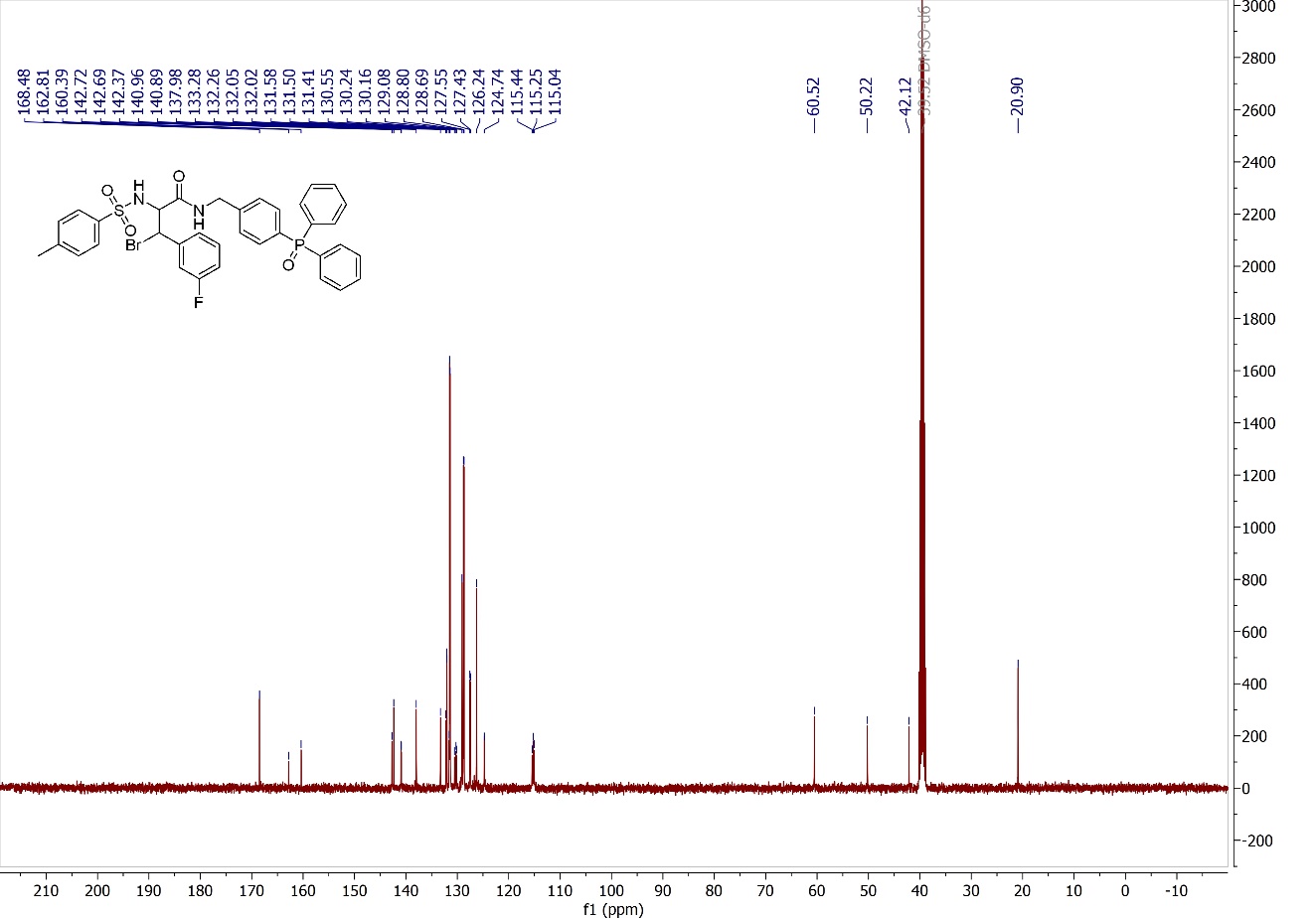


**4g**


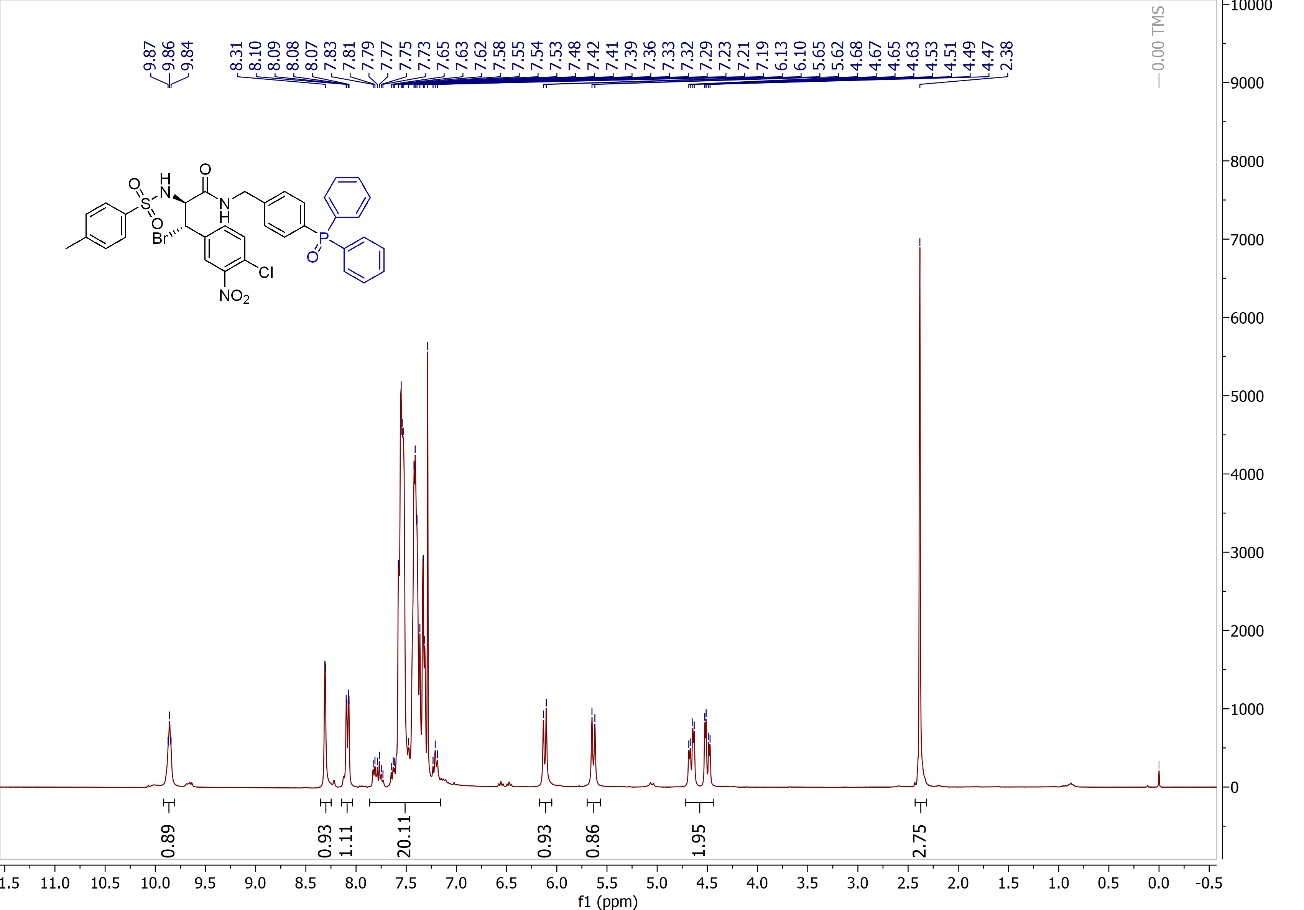

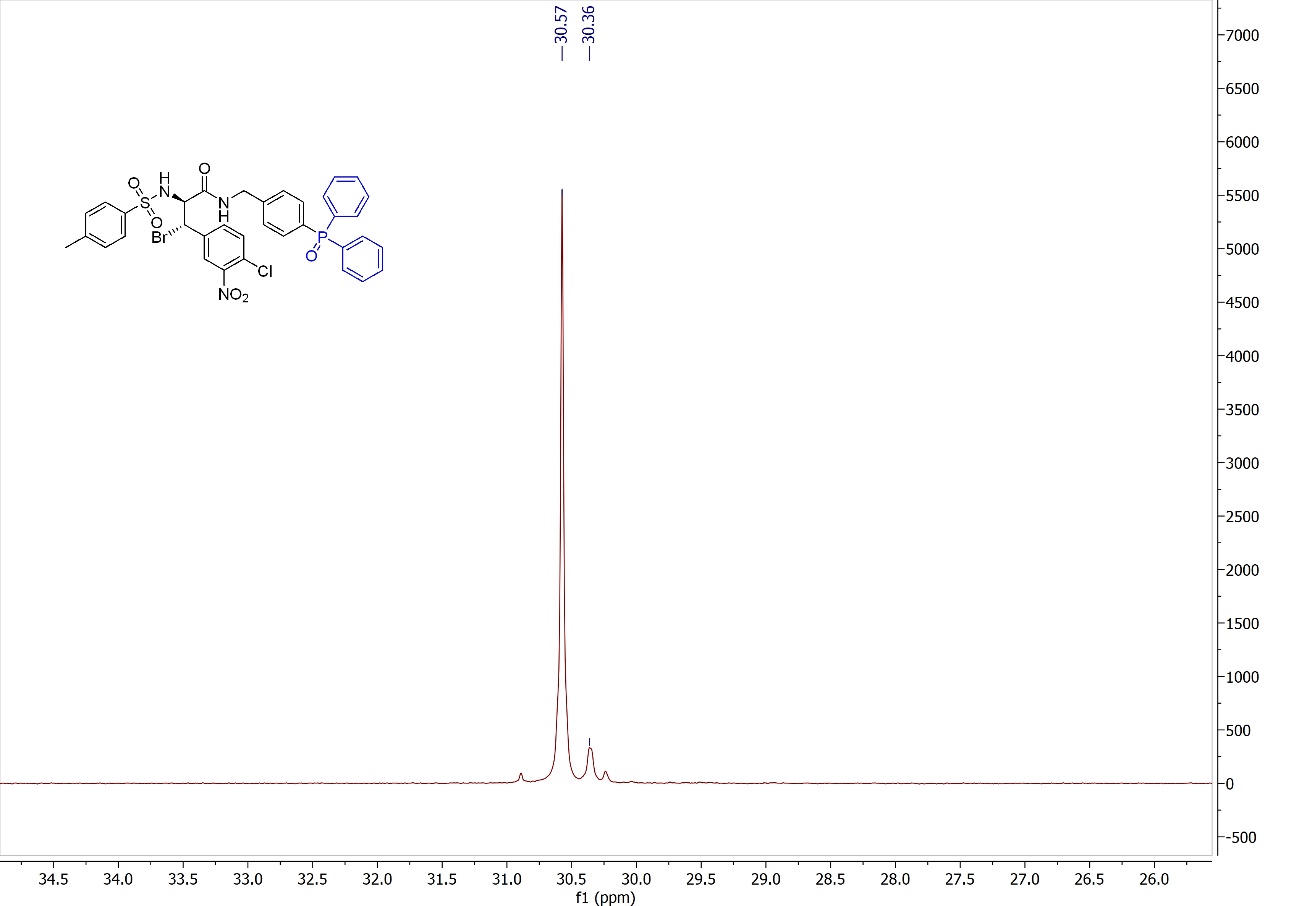


**4j**

**4j**


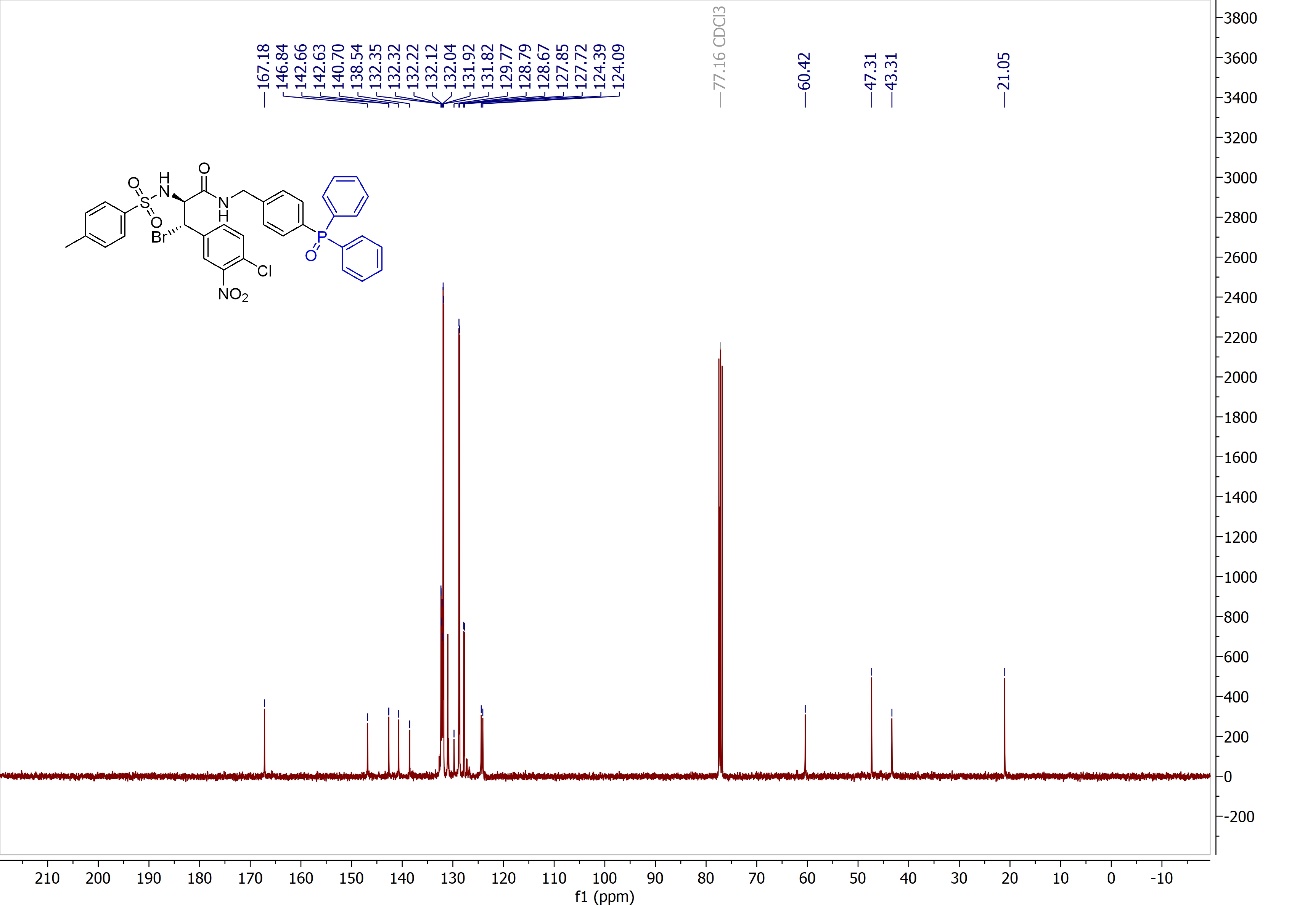


**4j**


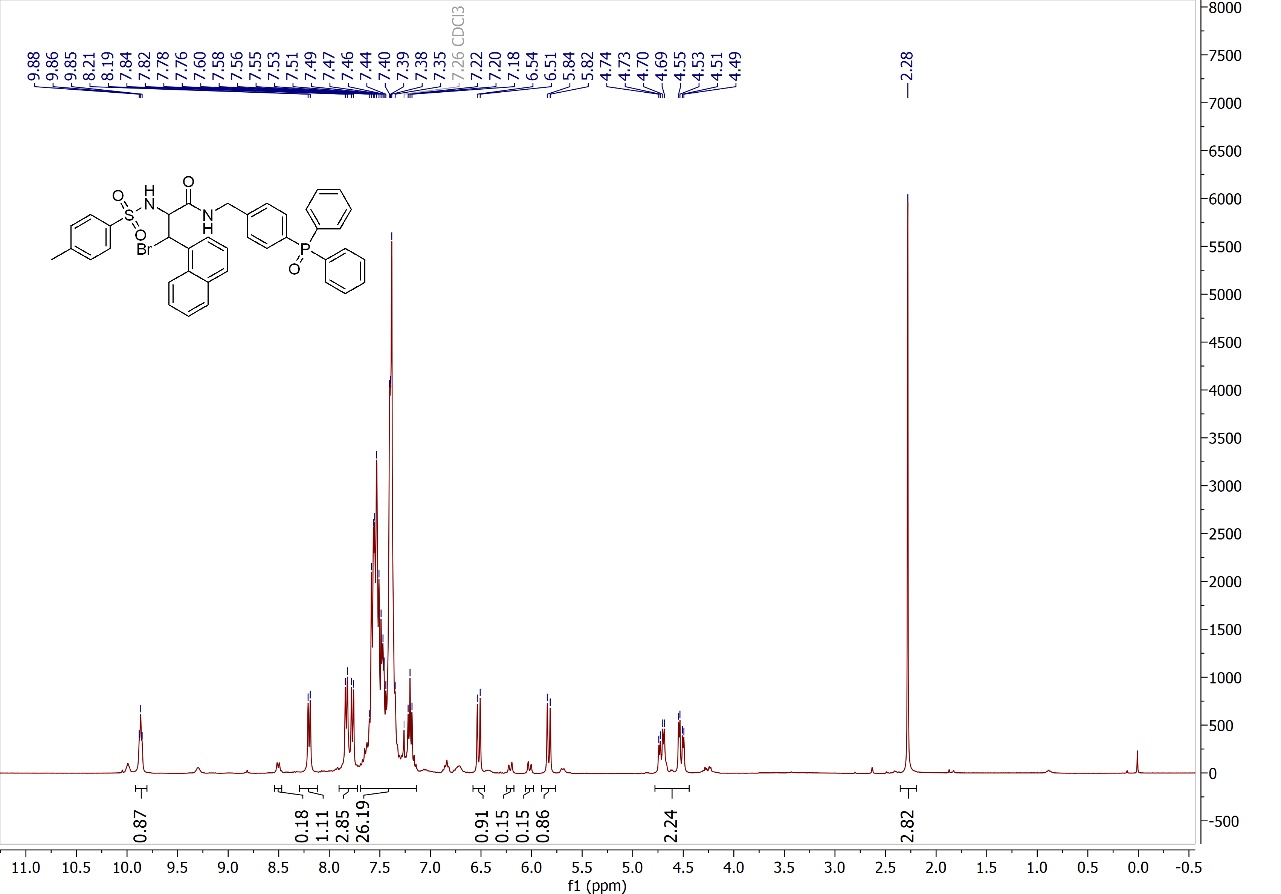

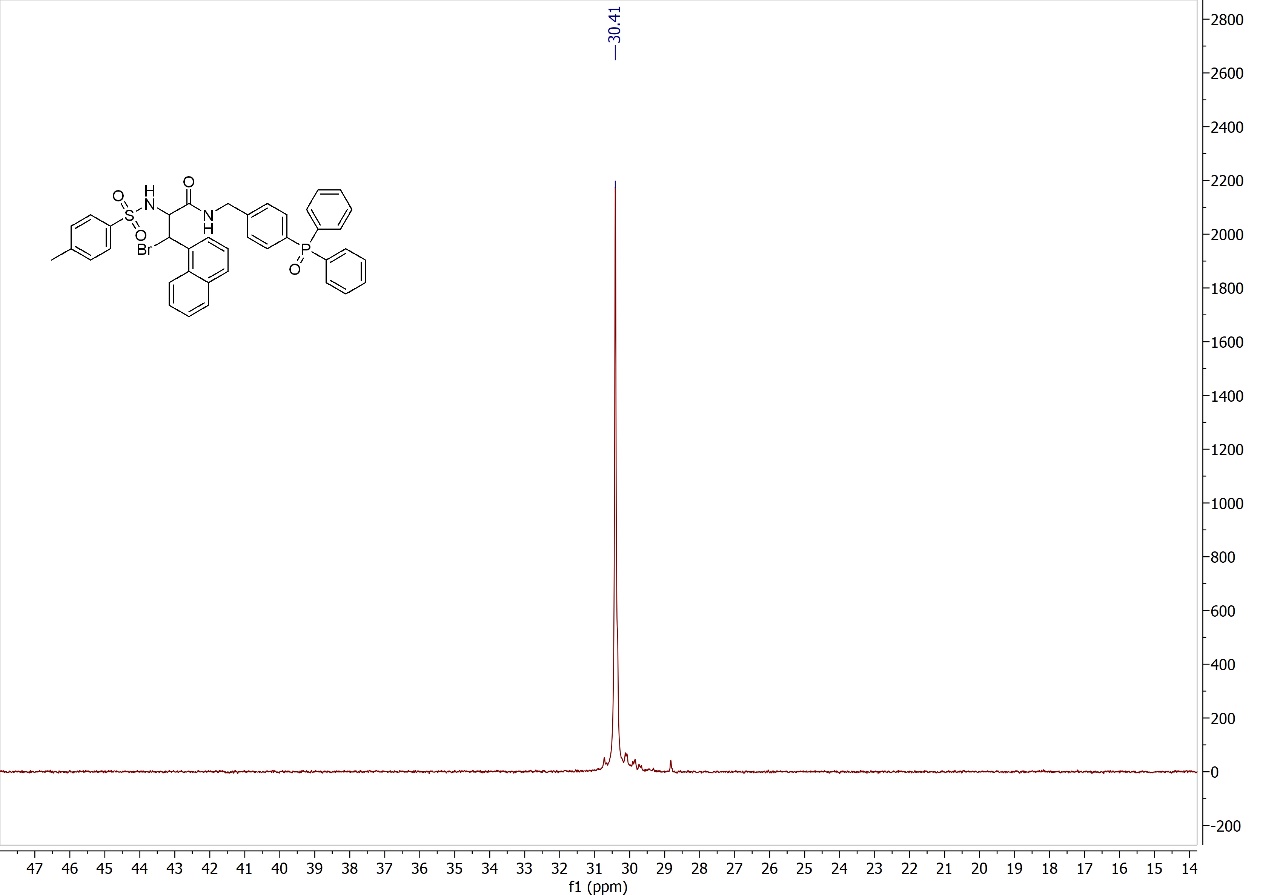


**4k**

**4k**


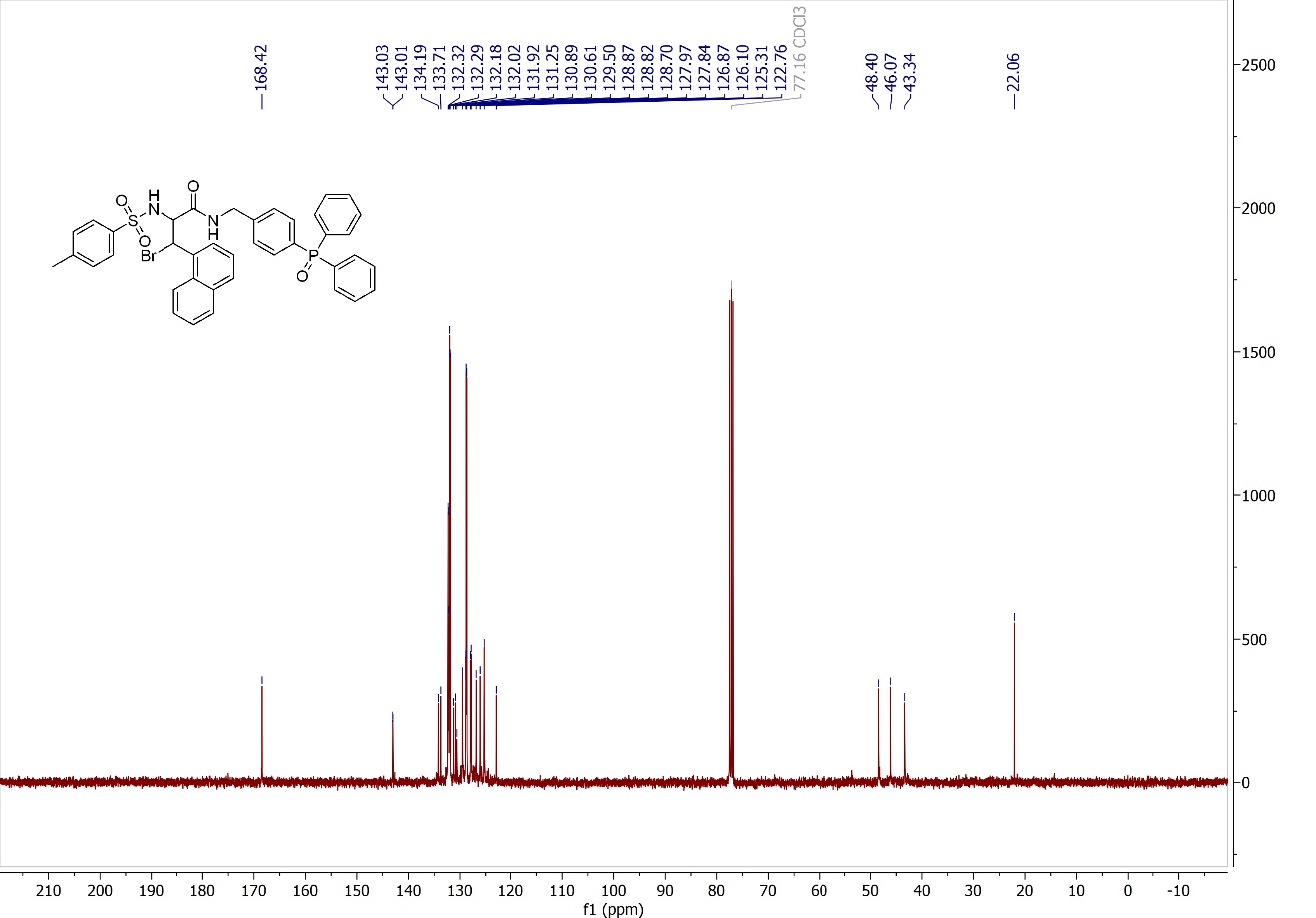


**4k**

# References

Hingst, M., Tepper, M., and Stelzer, O. (1998). Nucleophilic phosphanylation of fluoroaromatic compounds with carboxyl, carboxymethyl, and aminomethyl functionalities − an efficient synthetic route to amphiphilic arylphosphanes. *Eur. J. Org. Chem.* 1998**,** 73-82. doi: 10.1002/(sici)1099-0682(199801)1998:1<73::Aid-ejic73>3.0.Co;2-m

Jablonkai, E., and Keglevich, G. (2015). Catalyst-free P–C coupling reactions of halobenzoic acids and secondary phosphine oxides under microwave irradiation in water. *Tetrahedron Lett.* 56**,** 1638-1640. doi: <https://doi.org/10.1016/j.tetlet.2015.02.015>

Janssen, M., Müller, C., and Vogt, D. (2009). ‘Click’ dendritic phosphines: Design, synthesis, application in Suzuki coupling, and recycling by nanofiltration. *Adv. Synth. Catal.* 351**,** 313-318. doi: 10.1002/adsc.200900058

Seifert, C. W., Paniagua, A., White, G. A., Cai, L., and Li, G. (2016). GAP peptide synthesis via design of new GAP protecting group: An Fmoc/tBu synthesis of thymopentin free from polymers, chromatography and recrystallization. *Eur. J. Org. Chem.* 2016**,** 1714-1719. doi: 10.1002/ejoc.201600026
